# Redox‐ and NIR‐Active Iron(III) Triradicals as Catalysts for Radical Polymerization of Acrylamides and Methacrylates

**DOI:** 10.1002/anie.202507231

**Published:** 2025-07-04

**Authors:** Sujit Das, Amul Jain, Subuhan Ahamed, Bhanendra Sahu, Sonam Suthar, Björn Schwarz, Christel Livia Mascarenhas, Sangita Mondal, Saroj Kumar Kushvaha, Sanjib Banerjee, Herbert W. Roesky, Kartik Chandra Mondal

**Affiliations:** ^1^ Department of Chemistry Indian Institute of Technology Madras Chennai 600036 India; ^2^ Department of Chemistry Indian Institute of Technology Bhilai Durg Chhattisgarh 491001 India; ^3^ Institute for Applied Materials (IAM) Karlsruhe Institute of Technology (KIT), Hermann‐von‐Helmholtz‐Platz 1 Eggenstein‐Leopoldshafen 76344 Germany; ^4^ Universität Göttingen, Institut für Anorganische Chemie, Tammannstrasse 4 Göttingen 37077 Germany

**Keywords:** Block copolymer, Low‐spin‐tri‐radical mediated radical polymerization, Methacrylates and Acrylamides, Reaction kinetics, Reaction mechanism by EPR

## Abstract

Two unprecedented redox‐active, low‐spin Fe(III)‐triradical complexes, [Fe(III)(SS‐NHC═S^•−^)_3_]·NHC═S (**1**·NHC═S; E═S) and [Fe(III)(SS‐NHC═Se^•−^)_3_] (**2**; E═Se) have been synthesized and structurally characterized by SCXRD. They were further characterized spectroscopically using IR, Raman, EPR, and UV–vis‐NIR spectroscopy. The low‐spin electronic configuration of the central Fe(III) ion (**1**) and the nature of the magnetic interaction between the Fe(III) center and the three unpaired electrons in **1** have been investigated by magnetic measurements. In addition, the bonding stability and electron density distribution in **1** were studied by quantum chemical calculations and correlated with experimental results. Finally, a series of well‐defined functional homopolymers were synthesized via catalytic polymerization reactions using [Fe(III)(SS‐NHC═S^•−^)_3_] (**1**) as a catalyst at ambient temperature. These reactions yielded poly(*N*, *N*‐dimethylacrylamide) (PDMA), poly(*N*‐isopropyl acrylamide) (PNIPAM), poly(dimethyl amino ethyl methacrylate) (PDMAEMA), and poly(benzyl methacrylate) (PBzMA) with low dispersities ranging from 1.2 to 1.22. The successful synthesis of various diblock copolymers confirmed excellent chain‐end fidelity of the synthesized homopolymers. These homopolymers and diblock copolymers highlight the versatile catalytic polymerization reactions of these Fe‐radical complexes. Herein, we present a report on the polymerization of various acrylamides and methacrylates using a redox‐active Fe‐dithiolene complex for the first time.

## Graphical abstract

A redox‐active low‐spin Fe(III)‐triradical complex was isolated in good yield and utilized as a catalyst for radical polymerization of acrylamides and methacrylates at ambient conditions.

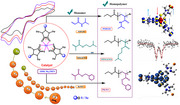

Reversible deactivation radical polymerization (RDRP) techniques, such as atom transfer radical polymerization (ATRP),^[^ ^1^ ^]^ reversible addition fragmentation chain transfer (RAFT) polymerization,^[^ ^2^, ^3^ ^]^ single electron transfer‐living radical polymerization (SET‐LRP),^[^ ^4^, ^5^ ^]^ and organometallic‐mediated radical polymerization,^[^ ^6^, ^7^ ^]^ have enabled the production of sequence‐defined polymers with high molar masses, defined macromolecular architectures, low dispersities, and residual end group functionalities. The Cu(0)‐RDRP and ATRP are multi‐component systems comprised of a metal source (Cu, Fe, Ni, etc.), a monomer (typically acrylates, acrylamides, methacrylates, etc.), an initiator (usually with a halide end group), a solvent, a deactivator, and additional components (e.g., photoactive dyes, salts, reducing agents, etc.).[^[^ ^8^ ^]^ Fe‐based catalysts have been previously attempted to be used in the RDRP reaction. Fe(0)‐mediated RAFT polymerization of methyl methacrylate (MMA) reactions were previously shown to proceed via single electron transfer (SET) from Fe(0) to R‐X, producing R^•^ intermediate.^[^ ^9^, ^10^ ^]^ Another report highlights the ATRP reaction of styrene catalyzed via elemental Fe(0)/Br_2_.^[^ ^11^ ^]^ Recently, Fe(0)‐mediated surface‐initiated ATRP reaction was shown to be enhanced by use of sea‐water, producing poly(oligoethylene glycol)methacrylate polymer brushes on the surface of an iron plate.^[^ ^12^ ^]^ However, the choice of catalyst plays a significant role in determining the rates of polymerization. Thus, to achieve controlled polymerization, a suitable catalyst is indispensable.^[^ ^13^ ^]^ Additionally, the concentration and activity of the ligand play a crucial role in polymerization reaction, with ligands varying between highly active [e.g., tris[2‐(dimethylamino)‐ethyl]amine (Me_6_TREN), tris(2‐pyridylmethyl)amine (TPMA)] and considerably less active [e.g., bipyridine (bpy)].^[^ ^8^ ^]^ Even though the reaction conditions of copper‐mediated ATRP have been judiciously optimized, the polymerization is often stopped with incomplete conversion to achieve high end group fidelity, which necessarily compromises the yield. The macroinitiator must be extensively purified by time‐consuming procedures.^[^ ^8^ ^]^ To achieve controlled functionality and structural properties of the polymers, metal‐mediated polymerization has emerged as a powerful technique. Radical polymerization reactions propagated by transition metals are an effective method for controlling the macromolecular architecture and functions of polymers.^[^ ^13^, ^14^, ^15^ ^]^ Among the transition metals used in RDRP, iron offers unique advantages due to its low toxicity, abundance, and ability to access multiple oxidation states, making Fe‐based systems attractive for developing redox‐active catalysts.^[^ ^9^, ^10^, ^11^, ^12^ ^]^ The metal‐free RAFT polymerization reaction was shown for the first time with an organic molecule containing an R─S─CS─R unit via the formation of R─(S─C═S)‐R radical intermediate.^[^ ^2^ ^]^ The Fe─S containing complexes have not yet been reported to catalyze the radical polymerization of acrylamides and methacrylates reactions.^[^ ^1^, ^2^, ^3^, ^4^, ^5^, ^6^, ^7^, ^8^, ^12^ ^]^ It is worth mentioning that iron–sulfur complexes are essential as cofactors of enzymes in biochemical processes.^[^ ^16^, ^17^ ^]^ Already in 1933,^[^ ^18^ ^]^ a spin‐crossover Fe(III) complex^[^ ^19^ ^]^ with a S_6_‐donor set was synthesized, isolated, and characterized using a monoanionic dithiocarbamate ligand [R_2_N─CS_2_^‐^].^[^ ^18^, ^19^ ^]^ A two‐sulfur donor ligand, called 1,2‐dithiolene (L), which is known as a redox non‐innocent ligand, has attracted the attention of chemists for several decades, leading to the synthesis, isolation, and characterization of several Fe‐dithiolene complexes.^[^ ^20^ ^]^ The elucidation of the electronic structures of these complexes, in particular the oxidation state of the iron center, is challenging due to the redox activity of the 1,2‐dithiolene ligand [L^0^, L,^‐^ and L^2‐^].^[^ ^21^, ^22^, ^23^, ^24^, ^25^, ^26^, ^27^, ^28^, ^29^, ^30^, ^31^, ^32^, ^33^, ^34^, ^35^, ^36^, ^37^, ^38^ ^]^ There is no report on the isolation and characterization of Fe(dithiolene‐radical)_3_ complex to date.^[^ ^20^, ^21^, ^38^ ^]^ Here, we report the synthesis, isolation, characterization, and catalytic activity in polymerization reactions of the redox and NIR‐active low‐spin Fe(III)‐triradical complex **1** with three *N*‐heterocyclic carbene‐derived 1,2‐dithiolene ligand [SS─NHC═S], which was recently developed by Robinson et al.^[^ ^39^, ^40^, ^41^, ^42^, ^43^ ^]^ Chemical bonding and the distribution of spin densities were clarified by DFT calculation, and the polymerization mechanism was supported by in situ EPR.

The synthesis of complexes **1** and **2** was carried out inside a glove box filled with argon. The lithium salt of dithiolene radical anion [Li(THF)_2_SS─NHC═E]/NHC═S^[^ ^39^ ^]^ [E═S, Se] was reacted with anhydrous FeCl_2_ or FeCl_3_ in THF for 6 or 2 h, respectively, at room temperature (rt) to form a dark purple‐brown solution of Fe(III)‐tri‐radical complex [Fe(SS─NHC═E)_3_] (E═S, **1**; E═Se, **2**) (Scheme 1). After removing THF under vacuum, the resulting solid residue was extracted with *n*‐hexane. The volume of the *n*‐hexane solution was reduced to one‐fourth of its initial volume and stored at room temperature for one day to form dark purple–brown rods of complexes **1**·NHC═S or **2,** which were isolated in yields of 75% and 35%, respectively. The yield of **1**·NHC═S was found to be 75% and 85% (based on ligand) when FeCl_2_ and FeCl_3_ were separately reacted with dithiolene radical ligand in 1:3 and 1:3 molar ratios, respectively (see Supporting Information for details). The powders of **1**·NHC═S and **2** are stable above 210 °C. Complex **1** is NIR‐active, exhibiting a broad absorption band at 1000 nm (*ε* = 4625 M^−1^ cm^−1^).^[^ ^26^ ^]^ The UV–vis bands are observed at 366, 554 (*ε* = 6783 M^−1^ cm^−1^), and 608 (*ε* = 3885 M^−1^ cm^−1^) nm. Strong IR stretching frequencies at 1455/1264 and 1090/803 cm^−1^ were assigned to C═C/C═N and C═S,^[^ ^26^ ^]^ respectively (see Supporting Information for **2**).^[^ ^39^ ^]^ Dark plates of **1**·NHC═S are stable in the open air for several minutes, and 50% decomposition takes about 15 h. A THF solution of **1** retains its purple color only for 15 min when exposed to air. The Raman spectrum of complex **1** shows intense bands at 126, 191, 205, 279, and 447 cm⁻^1^, corresponding to S─Fe─S stretching, Fe─S─C stretching, Fe─S asymmetric stretching, and Fe─S symmetric stretching, respectively. For Complex **2**, bands at 205, 234, and 243 cm^−1^ are attributed to the Fe‐S bond stretching frequency.^[^ ^35^, ^36^, ^37^, ^38^ ^]^

**Scheme 1:**
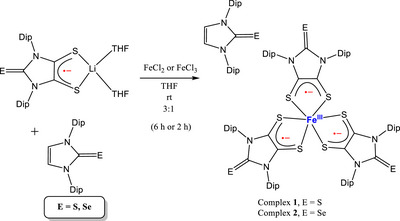
Synthesis of low‐spin‐Fe(III)‐triradical complexes [Fe(SS─NHC═S)_3_]·NHC = S (**1⋅**NHC═S) and [Fe(SS─NHC═Se)_3_] (**2**). Where, Dip = 2,6‐diisopropylphenyl.

Complexes [Fe(SS─NHC═S^•−^)_3_]·NHC═S (**1**·NHC═S) and [Fe(SS─NHC═Se)_3_] (**2**) crystallize from *n*‐hexane as dark rods in the orthorhombic *P*2_1_2_1_2_1_ and monoclinic C*2/c* space groups, respectively (Figure 1). SCXRD and structure refinement showed that **1** and **2** contain one Fe ion and three SS─NHC═E^•−^ ligands (E═S, Se), each of which chelates to the central Fe ion, forming a distorted FeS_6_ octahedron^[^ ^18^, ^44^, ^45^, ^46^, ^47^ ^]^ with three similar sets of Fe─S bond distances [E═S, ∼2.25 and ∼2.30 Å, **1**; E═Se, 2.2687(10)‐2.2903(10), **2**].^[^ ^44^, ^45^, ^46^, ^47^ ^]^

**Figure 1:**
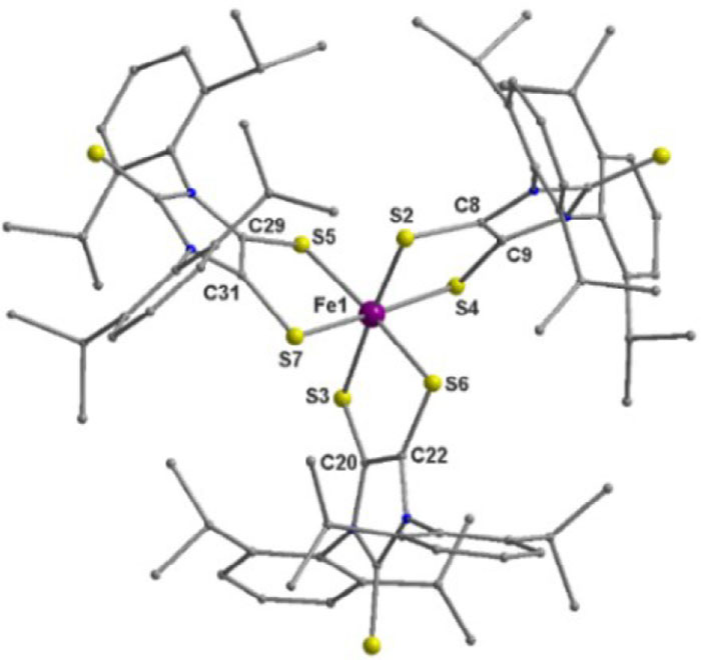
Molecular structure of complex [Fe(SS─NHC═S)_3_]·NHC═S (**1⋅**NHC═S) molecule, and all the H atoms were omitted for clarity. Bond parameters [Å]: Fe1‐S3 2.2498(13), Fe1‐S6 2.3020(13), Fe1‐S2 2.2954(13), Fe1‐S4 2.2658(13), Fe1‐S5 2.2783(13), Fe1‐S7 2.3011(13), C8–C9 1.405(6), C20–C22 1.405(7), C29–C31 1.403(7); S4–Fe1‐S2 92.29(5), S3‐Fe1‐S6 92.47(5), S5‐Fe1‐S7 92.63(4), S3‐Fe1‐S2 172.82(5), S4‐Fe1‐S7 172.40(5), S5‐Fe1‐S6 171.88(5). See SI for details bond parameters of complex **2**.

The bond parameters fall within the previously reported range. Charge balance considerations led to the conclusion that the oxidation state of Fe is +3.^[^ ^44^, ^45^, ^46^, ^47^ ^]^ Very recently, Al(SS─NHC═S)_3_ was reported to have an *S* = 1/2 spin ground state and a low‐lying *S* = 3/2 state. This Al(III)‐triradical complex, containing three dithiolene‐radical ligands, exhibits a quartet ground state (*S* = ½ or 3/2), challenging the traditional focus on transition metals for radical species and highlighting aluminum's potential as an open‐shell species, thereby expanding the possibilities for main‐group radical chemistry.^[^ ^41^ ^]^ The C─C bond lengths [∼1.405(7) Å] of each [SS─NHC═S] ligand in complex **1** are very similar to those previously reported values NHC‐base dithiolene radical anion ligand [1.417(3), 1.394(7)/1.388(4), 1.420(5) Å],^[^ ^39^, ^40^, ^41^, ^42^, ^43^ ^]^ suggesting that complex **1** contains one Fe(III) ion with three anionic radical ligands (SS─NHC═S^•−^).

The temperature‐dependent magnetic susceptibility of **1**·NHC═S was measured in the temperature range of 2–300 K. The value of the *χ*T product of **1** is 1.52 cm^3^ K mol^−1^ at 300 K which is significantly smaller than theoretically calculated value (5.50 cm^3^ K mol^−1^) for three isolated radicals [*g* = 2, *S* = ½; 0.375*3═1.125 cm^3^ K mol^−1^] and a high‐spin Fe(III) ion [*g* = 2, *S* = 5/2; 4.377 cm^3^ K mol^−1^]. However, it is very close to the expected value of three isolated radicals and a low‐spin Fe(III) ion [*g* = 2, *S* = ½; 0.375*4 = 1.50 cm^3^ K mol^−1^].

This confirms that the Fe(III) ion of complex **1** possesses a low‐spin electronic configuration^[^ ^34^ ^]^ with a distorted octahedral coordination geometry (Figure 2). Mössbauer measurement of **1** at 80 K (Figure 2, bottom) produced an isomeric shift of 0.34 mm s^−1^ and quadrupole splittings |Δ*E*_Q_| = 1.23, which are the range of low‐spin Fe(III) ions with *S*_Fe(III)_ = 1/2. These values are typical for low‐spin Fe(III) complexes with a FeS_6_ coordination environment.^[^ ^33^, ^47^ ^]^ A tris‐dithiocarbamate‐iron(III) complex with a S_6_ donor set was shown to display spin‐crossover behavior in 1930s.^[^ ^19^ ^]^ The value of the *χ*T product of **1** remains nearly constant from 300 K down to 50 K, below which it slowly decreases to 0.70 cm^3^ K mol^−1^, close to the calculated value of 0.75 cm^3^ K mol^−1^ for two isolated radical electrons. The decrease of *χ*T product below 50 K is most likely due to the antiferromagnetic coupling of a low‐spin Fe(III) ion with three unpaired electrons on radical anion ligands SS─NHC═S^•−^. The fitting of the *χ*T versus T plot produces *J*_Fe‐radical_ = −2.43(2) cm^−1^ and *g* = 2.122(2) for the central Fe(III) ion. The magnetization of **1** did not saturate even at 7 T, reaching a maximum value of 1.15 μ_B_ at 2 K. This may be due to the weak superexchange interactions between the unpaired electrons via Fe(III) (Figure 2, top).

**Figure 2:**
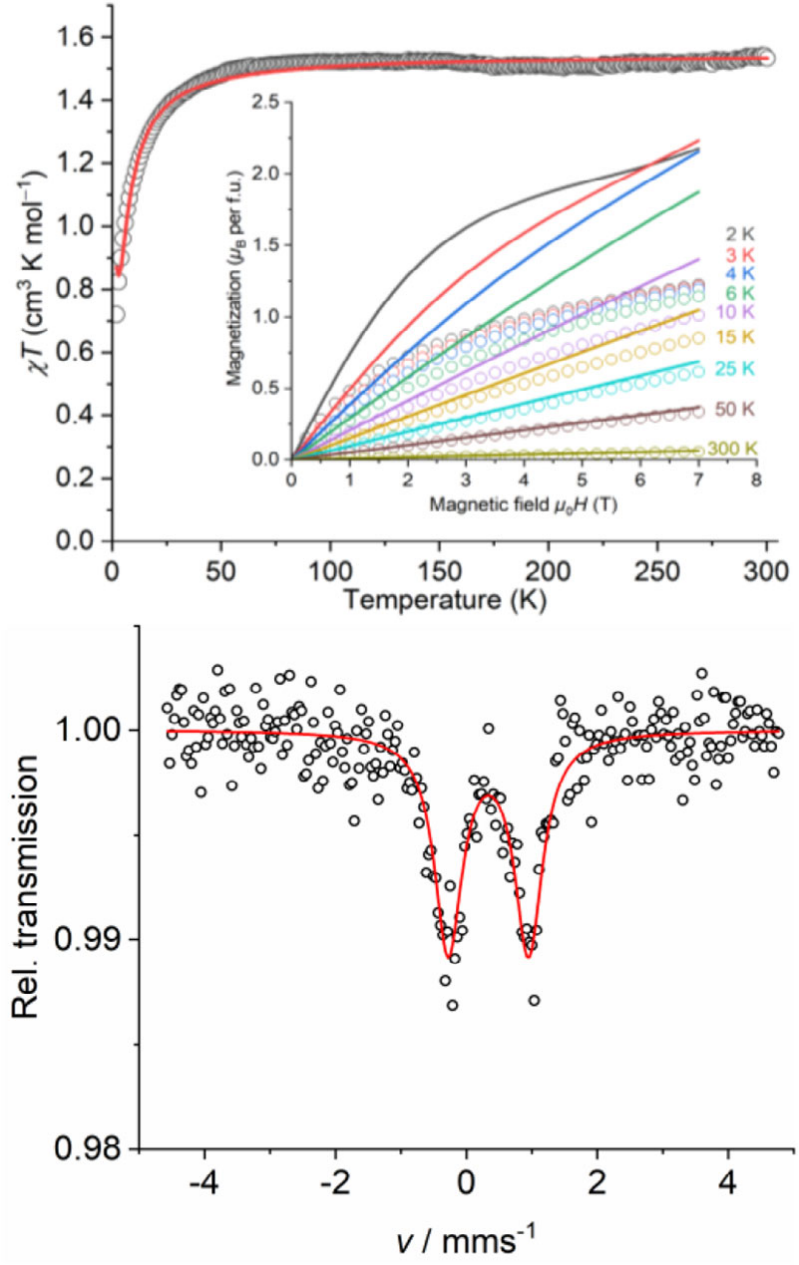
*χ*T versus T, magnetization versus magnetic field plots of complex **1** (top). Open circles represent measured values, lines represent fitting of the quantum mechanical magnetic model. Zero‐field ^57^Fe Mössbauer spectrum of **1** (bottom) at 80 K (open circles) and best fit curve (red line).

Thus, the complex [Fe(SS─NHC═S^•−^)_3_] (**1′**; Dipp units replaced by Me) was optimized using the B3LYP‐D3(BJ)/Def2‐TZVP level of theory in triplet spin ground state, based on information obtained from the magnetic susceptibility measurements. NBO analysis shows that three radical electrons of **1′** occupy the α‐SOMO, α‐SOMO‐1, and α‐SOMO‐2 (Figure 3a–c), while the unpaired electron on Fe(III) is located on β‐LUMO. The distribution of Mulliken spin densities (α‐spin on three ligands, 3 e^−^, and β‐spin on the Fe(III) center, 1 e^−^] is shown in (Figure 3d–e). The values are similar for the two dithiolene radical anions, each of which is chelated to the Fe(III) center from axial and equatorial sites, while another ligand forms two equatorial Fe─S bonds. Three Fe─S bond lengths in **1** are around 2.25 Å, while the other three are around 2.30 Å due to Jahn–Teller distortion, which has been studied by X‐ray data collection at 100 and 298 K. The bond parameters remain nearly the same (Table S2). A similar effect has been observed in previously reported Fe(III)–cyano‐dithiolene complexes.^[^ ^33^ ^]^ This leads to three different sets of radical–Fe(III) interactions, the effects of which were also observed in the EPR spectrum of **1** in solution.

**Figure 3:**
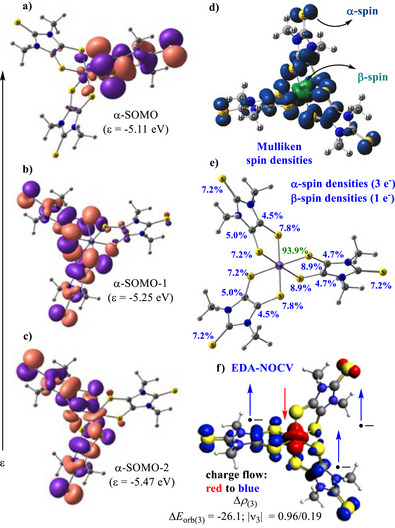
a)–c) NBOs (α‐SOMO, α‐SOMO‐1, α‐SOMO‐2), d)–e) Mulliken spin densities and f) The shape of the deformation densities ∆*ρ*_(3)_ that correspond to ∆*E*_orb(3)_, and the associated molecular orbitals of [Fe(SS─NHC═S)_3_] (**1′**) for L_2_Fe─L bond as L_2_Fe^+^ (D) and L^−^ (D) fragments in electronic doublet states at the B3LYP‐D3(BJ)/def2TZP level of theory [L = SS─NHC═S^•−^].

EDA‐NOCV analysis of **1′,** considering two charged fragments [(SS─NHC═S^•−^)_2_Fe(III)]^+^ and SS─NHC═S^•−^ in doublet states, shows that the major stabilization (∼60%) is due to the electrostatic interaction, with a minor contribution (36%) from orbital interaction energy. The lone pairs of electrons on the two S‐atoms of SS─NHC═S^•−^ form two sets of covalent dative bonds from SS─NHC═S^•−^→Fe(III) (∼58%; 70.8 kcal mol^−1^), while the radical electron on SS─NHC═S^•−^ is donated to HOMO/LUMO of [(SS─NHC═S^•−^)_2_Fe(III)]^+^ fragment, contributing significant stabilization energy (26.1 kcal mol^−1^; ∼21.6%, see Supporting Information). The total stabilizing interaction energy (Δ*E*_int_) between the Fe(III) center and one SS─NHC═S^•−^ is −160.8 kcal mol^−1^.

The EPR spectrum of the NMR‐silent complex **1**, recorded in THF solution at room temperature (rt), showed three sets of multiplets near *g* ≈ 2 [*g*_x_ = 2.01592, *g*_y_ = 2.01113, *g*_z_ = 2.00777], exhibiting rhombic symmetry. Each set of multiplets originates from a radical electron interacting with two ^14^N‐nuclei [*I* = 1, 99.57%; *A*_x_ 2.44–5.34 *A*_y_ 1.39–4.94 *A*_z_ 2.84–5.19 MHz; (Figure 4), with further details in the Supporting Information. The effect of coupling between the radical electron and the Fe(III) center is negligible due to the low abundance of spin‐active iron nucleus [^57^Fe, *I* = −1/2; 2.11%]. The predominant nature of the EPR signal of **1** was further evident from the shape of the EPR spectrum recorded at 77 K, which is due to the well‐known radical–radical dipolar interaction.

**Figure 4:**
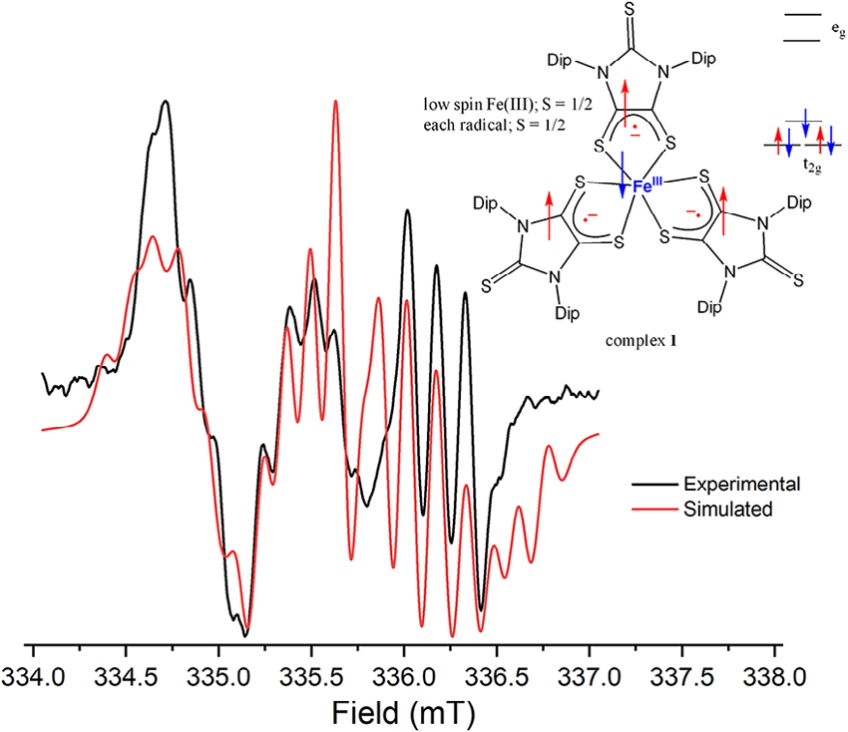
Experimental (black) and simulated (red) EPR spectra of complex **1**. Fitting parameters: Fe(III), low spin; *S*_FeIII_ = 1/2 coupled with three radicals of each *S*_radical_ = 1/2.

CV measurements of **1** in THF solution showed multiple redox events in the potential range −2.0 to ‐0.4 V (Figure 5).

**Figure 5:**
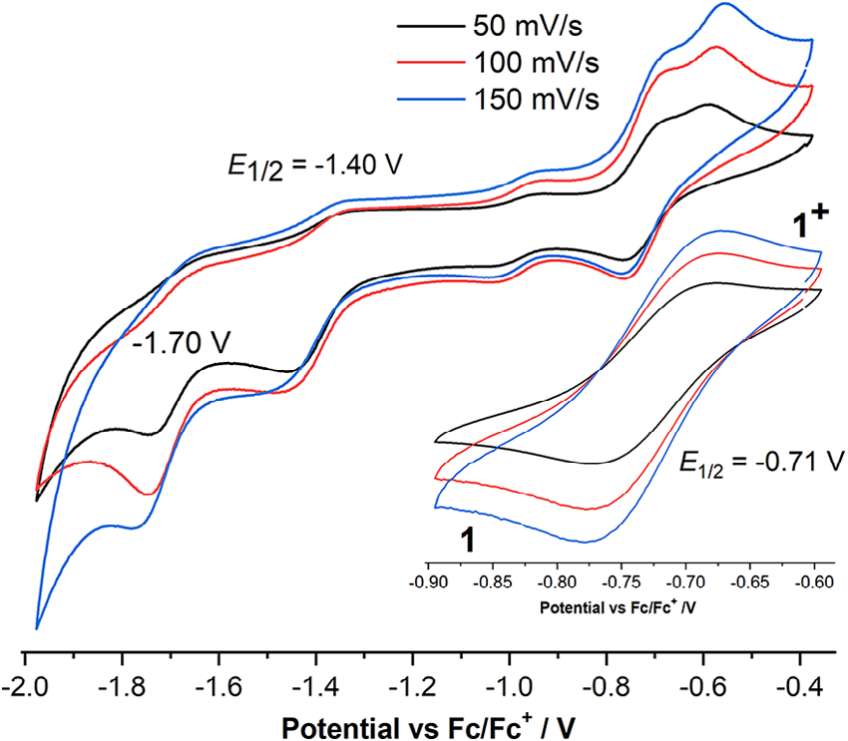
The cyclic voltammogram of complex **1** in THF at rt containing 0.1 M [*n*‐Bu_4_N][PF_6_] as supporting electrolyte, at scan rates of 50/100/150 mV s^−1^. Reduction (left) and oxidation (right). Glassy carbon as WE (working electrode), Platinum wire as CE (counter electrode). Ag Wire as RE (Reference Electrode). The potential (*V*) is plotted against the standard reduction potential of Cp_2_Fe^+^/Cp_2_Fe. The voltage ranges from 0 V to −1.6 V. See Supporting Information for CV of **2**.

A reversible electronic process at *E*_1/2_ = −0.71 V can be assigned to the loss of an electron from one of the three chelated radical anionic SS─NHC═S^•−^ ligands, suggesting the formation of [(SS─NHC═S^•−^)_3_Fe(III)]^+^ (**1^+^**) which is very close to the value (−0.78 V) reported for ligand centered π‐electron of [(THF)_2_Li^+^(SS─NHC═S^•−^].^[^ ^39^, ^40^, ^41^, ^42^, ^43^ ^]^ Two additional ligand‐centered redox events were observed around −1.40 [L^2‐^←L^•−^] and ‐1.70 V [Fe(II)←Fe(III)].^[^ ^26^ ^]^ The *E*_1/2_ values of **2** were shifted toward positive potential (∼0.2 V; see Supporting Information).

Having the redox‐active Fe(III)‐triradical complex **1** [FeL_3_; L═SS─NHC═S^•−^] in hand, we next carried out polymerization of acrylamides and methacrylates at ambient conditions. Initial optimization of the complex **1**‐mediated controlled organometallic‐mediated radical polymerization (OMRP) of dimethyl acrylamide (DMA) in THF (Table S10) revealed that well‐defined PDMA could be produced using EBiB as the initiator and Me_6_TREN as the ligand under ambient reaction conditions (Figure 6a). The synthesis of PDMAs with varying *M*_n_ (up to 15045 g mol^−1^) and low *Đ*_s_ (≤1.20) (Table S11) was studied through SEC measurements. Structural analysis of the produced PDMA_50_‐Br was performed by ^1^H NMR (Figure 6b), which exhibited the typical signals of DMA in the ^1^H NMR (600 MHz, CDCl_3_, δ ppm of P7, Table S11) at 1.5 ppm (–CH_2_C(CH_3_) of DMA), 1.5–2.1 ppm (‐CCH_2_ connecting DMA chains), 2.1–2.5 ppm (‐CH of DMA chains), and 2.6 ppm (–N(CH_3_)_2_ of DMA).^[^ ^48^ ^]^ The ATR‐IR spectrum of the PDMA_50_‐Br (entry P2, Table S11, Figure S28) shows characteristic bands at 2927, 2729, 1610, 1016 cm^−1^, corresponding to ‐NH, ‐CH, ‐CONH, and ‐C─N groups, respectively.^[^ ^48^ ^]^

**Figure 6:**
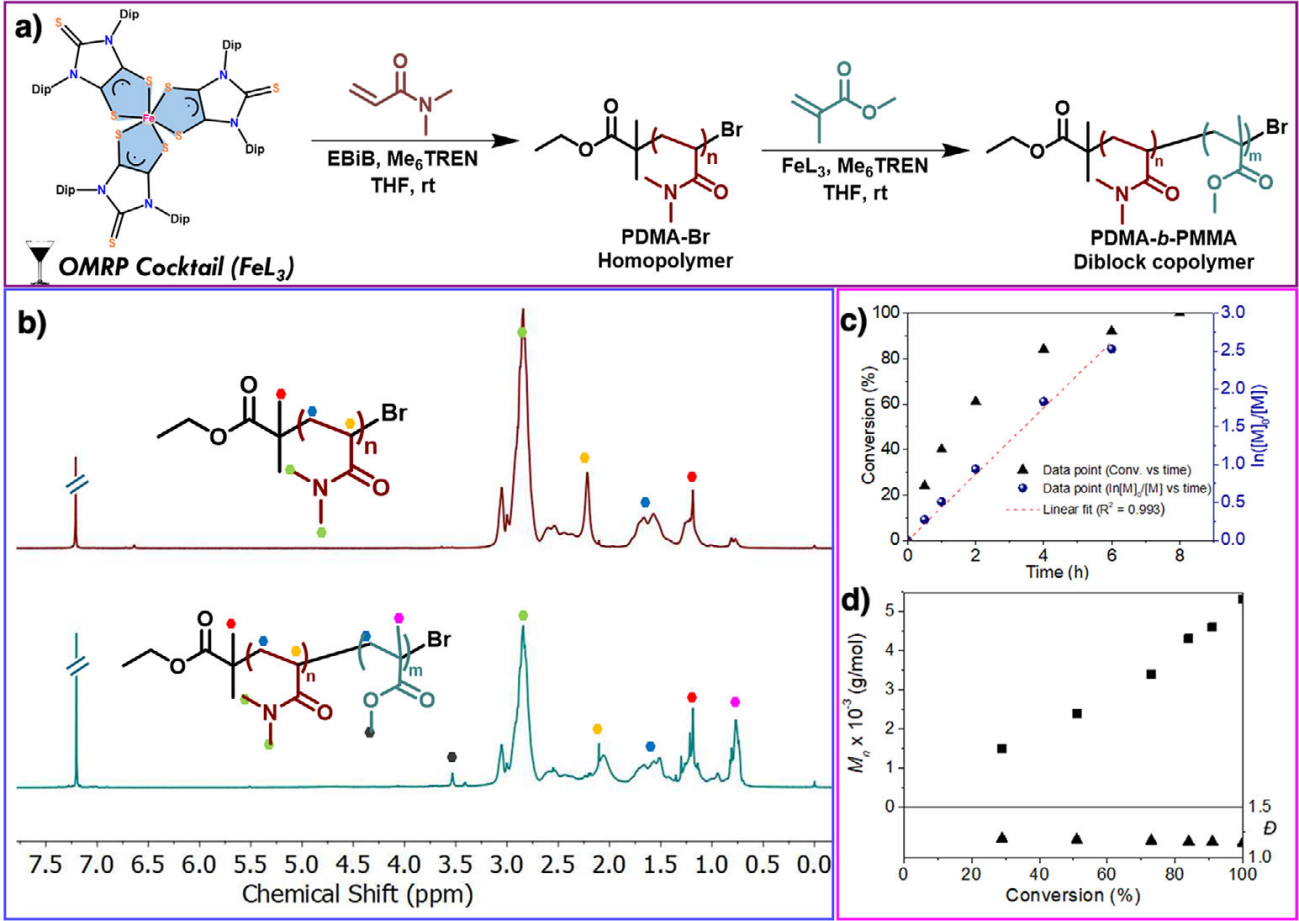
a) Complex **1** catalyzed homopolymerization of DMA and synthesis of PDMA‐*b*‐PMMA diblock copolymer. b) ^1^H NMR spectrum of PDMA_50_‐Br and PDMA_50_‐*b*‐PMMA_30_‐Br diblock copolymer. c) Plot of ln([M]_0_/[M]) and % conversion versus time. d) Evolution of molar mass (*M*_n_) and dispersity (*Đ*) with increasing DMA monomer conversion.

Employing DMA as a model system, control polymerization reactions without an initiator, Me_6_TREN, and complex **1** (P4–P6, Table S10) showed no polymerization. However, the polymerization reaction with all the components led to a well‐defined polymer (P3, Table S10). These studies demonstrated that the presence of each of these components catalyst (complex **1**) and Me_6_TREN is necessary for a successful polymerization reaction. Interestingly, polymerization with the dithiolene radical anion [(THF)_2_Li(SS‐NHC═S)] resulted in no polymerization (P7, Table S10). However, substituting Me_6_TREN with 2,2′‐bipyridine (bpy) or N,N, N′,N″,N″‐pentamethyldiethylenetriamine (PMDETA) led to extremely sluggish polymerization and ill‐defined polymers. (P1–P2, Table S10).

A kinetic investigation of the polymerization of PDMA_50_‐Br was conducted through a series of reactions (range from 0.5 to 8 h, P2–P7, Table S11). The first‐order kinetic plot ln([M]_0_/[M]) versus time (Figure 6c), which is considered “linear,” yields an apparent propagation rate constant {*k*_p(app)_}. A linear increase in *M*_n_ with conversion of monomer (exhibiting low *Đ_s_* values) (Figure 6c) was observed. The *M*_n_ versus conversion plot (Figure 6d) shows low *Đ_s_* values (1.14–1.20) and a monotonic linear growth that is in line with RDRP characteristics.

The combination of Fe‐salt and TEMPO radical has proven to be very fruitful for organic transformation in the past.^[^ ^49^, ^50^, ^51^ ^]^ The mechanism for Fe(III)‐triradical‐mediated RDRP of the acrylamide(s) and methacrylate(s) is hypothesized in Figure 7, based on in situ EPR measurements and EPR simulations. The N_4_‐donor external ligand (Me_6_TREN) slowly knocks out one dithiolene radical anion (free SS─NHC═S^•−^), producing a characteristic low‐intensity EPR signal (five hyperfine lines, ^14^N═MHz)^[^ ^39^ ^]^ at *g* = 2.0065 after 5 min. A relatively higher‐intensity signal at a slightly higher *g* value (2.0112) has been assigned to the Me_6_TREN‐coordinated Fe‐complex [(SS─NHC═S^•−^)_2_Fe(III)(Me_6_TREN)]^+^ (**INT‐2**).^[^ ^39^, ^40^, ^41^, ^42^, ^43^ ^]^ The reaction mechanism is shown in Figure 7. Free SS─NHC═S^•−^ was experimentally found not to give polymerization product, excluding the possibility of the SET mechanism. The combination of radical‐Fe is required as shown in Figure 7 (see Supporting Information for detailed explanation). Very recently, tandem polymerization employing a five‐coordinate Fe(II) complex was reported.^[^ ^52^, ^53^ ^]^

**Figure 7:**
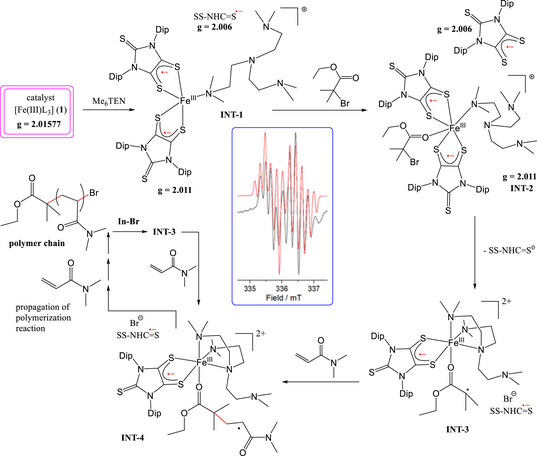
Proposed polymerization reaction mechanism catalyzed by [Fe(SS─NHC═S)_3_] (**1**) employing Me_6_TREN ligand supported by time‐dependent EPR measurement of reaction solution [E═S]. Black and red lines represent experimental and simulated EPR spectra after 65 min of the reaction solution (see Supporting Information).

To establish chain end fidelity, we have further synthesized a sequence of well‐defined diblock copolymers with methyl methacrylate (MMA) (Table S13, Supporting Information), such as PDMA_50_‐*b*‐PMMA_30_‐Br (Figure 6). Typically, the ^1^H NMR spectrum of the PDMA_50_‐*b*‐PMMA_30_‐Br diblock copolymer (Figure 6b) shows the different signals of the PDMA first block and PMMA second block at 1.5 ppm (–CH_2_C(CH_3_) of DMA and MMA), 1.5–2.1 ppm (–CCH_2_ connecting DMA chains and MMA), 2.1–2.5 ppm (‐CH of DMA chains), 2.6 ppm (–N(CH_3_)_2_ of DMA) 3.0–3.5 ppm (‐OCH_3_ of MMA). The ATR‐IR spectrum of the PDMA_50_‐*b*‐PMMA_30_‐Br sample (Figure S31) exhibited the main characteristic peaks of the PDMA and PMMA segments: 1132, 1610, 1733, 2854, and 3286 cm^−1^ corresponding to C─O─C (stretch), ‐CONH, ‐C═O (stretch), ‐C─H (stretch), and −NH (stretch), respectively. Another diblock copolymer, poly(N‐isopropylacrylamide)‐*block*‐poly(N,N‐dimethylacrylamide) PNIPAM‐*b*‐PDMA‐Br, was synthesized and characterized using IR and ^1^H NMR (Table S13, Figures S32–S33). Furthermore, to demonstrate the versatility of the catalyst, we performed the homopolymerization of various monomers including N‐isopropylacrylamide (NIPAM), dimethylaminoethyl methacrylate (DMAEMA) and benzyl methacrylate (BzMA) with complexes **1**, **2,** and **3** (Table S12) having low dispersities ranging between 1.2–1.22 (Figure 8). However, the polymerization catalyzed by complex **2** is slower than that by complex **1**. The catalytic activity of complexes **2** and **3** was similar, irrespective of the groups in the complexes. These homopolymers were characterized using ATR‐IR and ^1^H NMR, respectively (Figures S29–S31).^[^ ^48^, ^52^, ^54^, ^55^, ^56^ ^]^ A kinetic investigation of the polymerization of NIPAM, DMAEMA, and BzMA was conducted through a series of reactions (Figures S32–S34), and polymers with low molecular weight in the range of 5000–10000 g mol^−1^ have been obtained. The first‐order kinetic plot ln([M]_0_/[M]) versus time reactions (Figures S32–S34) and a linear increase in *M*_n_ with conversion of monomer (exhibiting low *Đ*_s_ values) was observed (Figures S32–S34), highlighting its characteristic nature.

**Figure 8:**
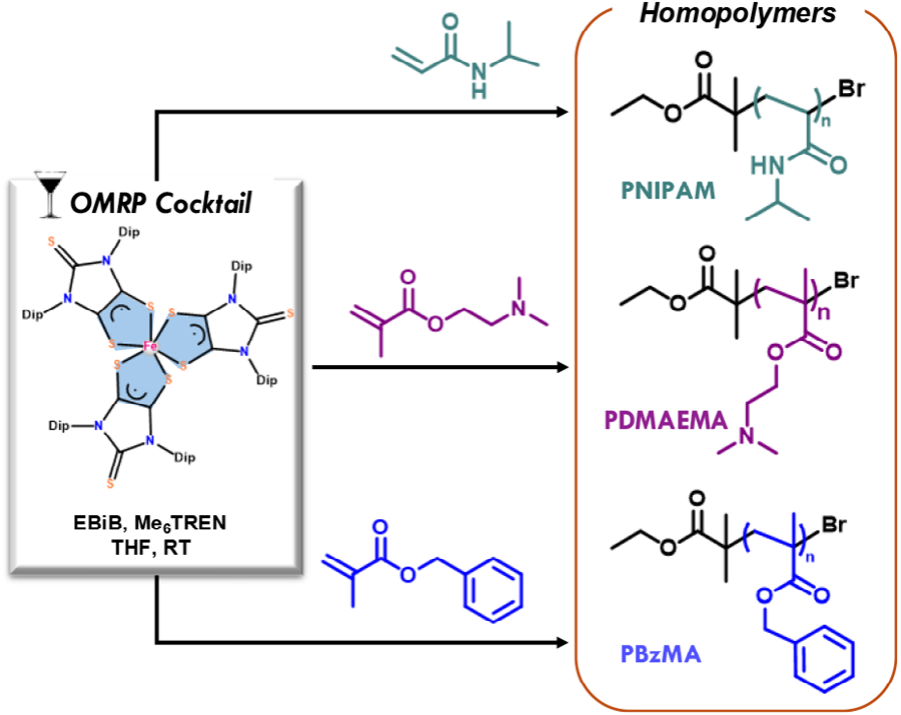
Homopolymerization of representative monomers through FeL_3_‐mediated polymerization [**1**, E═S, **2**, E═Se].

Recent research has investigated various polymerization strategies involving metal‐based catalysts. One approach explored Fe(II)‐complex‐mediated polymerization, which led to uncontrolled polymerization with high dispersity values (*Đ* = 1.39–3.5).^[^ ^52^ ^]^ Similarly, the photopolymerization of acrylates has been studied using Mn‐based Schiff‐base complexes, resulting in high dispersity (*Đ* = 2.0–2.8).^[^ ^57^ ^]^ In another study, radical polymerization reactions initiated by alkyl halides, using a vitamin B12‐derived cobalt complex, were shown to proceed effectively at temperatures between 40 °C and 60 °C.^[^ ^58^ ^]^ These studies highlight the diverse approaches in metal‐catalyzed radical polymerization and the challenges associated with controlling polymer dispersity.

In conclusion, this communication reports two unprecedented iron‐dithiolene‐based Fe(III)‐tri‐radical complexes as a versatile catalyst for the polymerization of various monomers under ambient reaction conditions. They were characterized employing different tools. The stability, bonding, and distribution of electron densities were studied by DFT/EDA‐NOCV calculation. Interestingly, this is the first report on the dithiolene‐based metal‐radical catalyzed radical polymerization with an unrivalled level of control, including high chain end fidelity. Moreover, such complex systems have only been studied computationally, however, we have highlighted their use here as a potential catalyst for the polymerization of various monomers and compared the catalytic activity of these catalysts. The catalyst is highly versatile, as methacrylates and acrylamides can be easily polymerized, and block copolymers can also be produced. Due to tunable chain lengths, these synthesized block copolymers could be used in various applications, such as self‐assemblies, micelles, and stimuli‐responsive materials.

## Supporting Information

It contains synthesis, characterization, CV, EPR, NMR plots, Figures, Tables, and optimized geometry/coordinates. The authors have cited additional references within the Supporting Information. CCDC number [2345820(**1**), 2404919(**2**)].

## Conflict of Interests

The authors declare no conflict of interest.

## Supporting information



Supporting Information

Supporting Information

## Data Availability

The data that support the findings of this study are available in the Supporting Information of this article.
