# Supplementary material for: Redox‐ and NIR‐Active Iron(III) Triradicals as Catalysts for Radical Polymerization of Acrylamides and Methacrylates
Source: Angew Chem Int Ed Engl. 2025 Jul 4;64(34):e202507231. doi: 10.1002/anie.202507231 (PMC12363635; doi:10.1002/anie.202507231)
Supplement: Supplementary file 1 — Supporting Information [file ANIE-64-e202507231-s002.pdf]

Redox- and NIR-active Iron(III) Triradicals as Catalysts for Radical Polymerization of Acrylamides and Methacrylates

Sujit Das,[a]† Amul Jain,[b]† Subuhan Ahamed,[a] Bhanendra Sahu,[b] Sonam Suthar,[a] Björn Schwarz, [c] Christel Livia Mascarenhas,[a] Sangita Mondal,[a] Saroj Kumar Kushvaha,[d] Sanjib Banerjee,[b]* Herbert W. Roesky,[d]* and Kartik Chandra Mondal[a]*

[a] M.Sc. Sujit Das, M.Sc. Subuhan Ahamed, M.Sc. Sonam Suthar, M.Sc. Sangita Mondal and Dr. Kartik Chandra Mondal*, Department of Chemistry, Indian Institute of Technology Madras. Chennai, 600036, India. E-mail: csdkartik@iitm.ac.in

[b] M.Sc. Amul Jain, M.Sc. Bhanendra Sahu and Dr. Sanjib Banerjee*, Department of Chemistry, Indian Institute of Technology Bhilai, Durg 491001, Chhattisgarh, India. Email: sanjib.banerjee@iitbhilai.ac.in

[c] Dr. B. Schwarz, Institute for Applied Materials (IAM), Karlsruhe Institute of Technology (KIT), Hermann-von-Helmholtz-Platz 1, 76344 Eggenstein-Leopoldshafen, Germany.

[d] Dr. S. K. Kushvaha, Prof. H. W. Roesky*, Institute of Inorganic Chemistry, Georg-August University, Goettingen, Germany. Email: hroesky@gwdg.de

†These authors contributed equally to this manuscript

Content:

1. General Synthesis

2. Synthesis

3. Magnetic properties calculations

4. Single crystal X-ray diffraction

5. EPR measurements of complex 1

6. IR spectra

7. UV-VIS-NIR measurements

8. CV measurements

9. EPR studies for catalysis reaction

10. Computational Methods

11. A. Experimental procedures for polymerization reaction

12. Mössbauer Measurements

13. EPR and DFT studies of complex 2

14. Raman Spectra

15. References

1 General Synthesis:

All the organic solvents (THF, *n*-hexane, *n*-pentane, toluene, Diethyl ether, C_6D_6) were soaked with 3 Å molecular sieves to remove water, followed by distillation with Na metal and NaK alloy three times under the flow of high-purity argon gas. All the manipulations were performed inside the glove box running with argon gas below the H_2O/O_2 level of 10 ppm. The dithiolene radical anion [(THF)$_2$Li(SS-NHC=Se)] was prepared by following a similar synthetic method, which led to the synthesis of [(THF)$_2$Li(SS-NHC=S)], reported by Robinson et al.[15-17] X-ray single crystal mounted using paratone oil under argon gas flow. Data was collected in Bruker D8 VENTURE model (machine) at 100 K. Data was refined using Apex-4 package. CV was measured in alloyed distilled THF in a potentiometer (Mterohm). NMR was recorded in C_6D_6 in a 500 MHz Bruker instrument at IIT Madras. EPR was simulated using the EASY-SPIN package.

2 Synthesis:

Synthesis of complex [Fe(SS-NHC=S)$_3$]·NHC=S (**1**) [E = S]:

The reaction was carried out inside the glove box filled with argon gas. The dark purple solid of lithium salt of dithiolene radical anion [Li(THF)$_2$SS-NHC=S]/NHC=S (759 mg; 1.2 mmol) was placed in 100 mL Schlenk flask to which THF (20 mL) was added at room temperature (rt) to obtain a dark purple solution after 10 min of stirring. Anhydrous $FeCl_2$ (52 mg, 0.4 mmol) was added to the resultant dark purple solution of [Li(THF)$_2$SS-NHC=S], and the reaction mixture was stirred for 6 h at rt to form a dark purple-brown solution of complex [Fe(SS-NHC=S)$_3$] (**1**). The THF was removed under vacuum, and dry mass was extracted with *n*-hexane (15 mL), and the volume of solution was reduced to around 3-4 mL. Dark purple-brown rods of complex **1**·NHC=S were formed after one day of storing the solution at rt. Yield = 75%. Decomposed to brown color liquid above 210 °C. UV-vis bands: 366, 554, 608, 1000 nm. IR (cm^{-1}): 2955, 1455, 1355, 1264, 1185, 1090, 1026, 973, 803, 708, 591. EPR active giving three sets of EPR signals near g ≈ 2. Complex **1** is NMR silent. Elemental analysis for $C_{108}H_{138}FeN_8S_{10}$ [calculated] (%): C 67.45 [67.39], H 7.18 [7.23], N 5.78 [5.82], matching with [Fe(SS-NHC=S)$_3$]·NHC=S. All measurements were carried out after isolation pure single crystals.

Alternative Synthetic Route: Synthesis of Complex **1** via Iron(III)-Induced Coordination of Dithiolene Radical Anion

The lithium salt of the dithiolene radical anion, $[Li(THF)_2SS\text{-}NHC{=}S]/NHC{=}S$ (380.4 mg, 0.6 mmol), was dissolved in 12 mL of dry tetrahydrofuran (THF) under an inert atmosphere at room temperature (rt). After stirring for 10 minutes, a vivid dark purple solution formed, indicating the presence of the free radical anion. Separately, anhydrous $FeCl_3$ (32.5 mg, 0.2 mmol) was dissolved in 5 mL of dry THF. This solution was added dropwise to the dark purple dithiolene solution. Upon addition, the reaction mixture immediately turned brownish purple, consistent with the coordination of the radical anions to Fe(III).

The mixture was stirred for an additional 2 hours to ensure complete reaction. The solvent was then removed under reduced pressure, and the resulting crude solid was extracted with 15 mL of *n*-hexane. After extraction, the hexane solution was concentrated under reduced pressure to reduce the volume to around 5 mL and then left undisturbed for crystallization. Remarkably, dark purple-brown needles formed within 1-2 hours. The isolated crystals were spectroscopically and morphologically identical to complex **1** previously obtained via a less efficient route. The overall yield of this improved method was approximately 85%**,** significantly higher than that of the earlier synthesis.

Note: Compared to the original method, this route results in high yield and requires a significantly shorter crystallization period (1-2 hours vs. 1 day), though it avoids the use of $FeCl_2$ and prolonged stirring time.

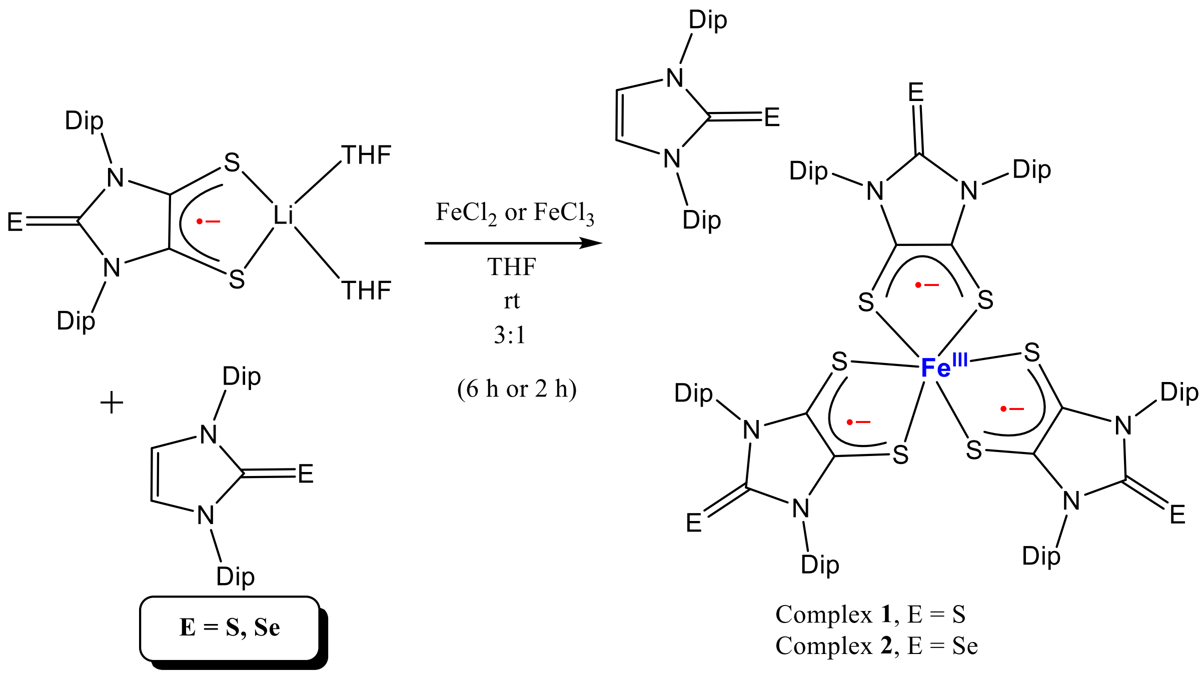

Scheme S1. Synthesis of complex [Fe(SS-NHC=S)$_3$]·NHC=S (**1**·NHC=S), where Dip = 2,6-diisopropylphenyl.

Synthesis of complex [(SS-NHC=Se)$_3$Fe] (**2**) [E = Se]:

The synthesis begins with the preparation of a striking dark blue solid, the lithium salt of the dithiolene radical anion [(THF)$_2$Li(SS-NHC=Se)], weighing 270 mg (0.4 mmol), which is dissolved in 10 mL dry tetrahydrofuran (THF) to form a vivid blue solution. In a separate flask, anhydrous iron(II)dichloride (FeCl$_2$, 50.71 mg, 0.4 mmol) is dissolved in 8 mL THF under an inert atmosphere, and potassium hydroxide (KOH, 45 mg, 0.8 mmol) is added at -40°C, inducing a light brown color change. After stirring for 30 minutes, the radical anion solution is introduced, causing the solution to shift from dark blue to dark purple, indicating the formation of the complex [(SS-NHC=Se)$_3$Fe] (**2**). The mixture is then stirred overnight, with the color deepening to a brownish-purple, indicating completion. Following solvent removal under vacuum, *n*-hexane is used to extract complex **2**. The resultant solution was filtered and allowed to stand undisturbed for several days; dark brownish purple crystals of complex **2** formed, which were isolated in 35% yield. IR (KBr, cm^{-1}): 2968, 2919, 2866, 1765, 1630, 1463, 1363, 1259, 1226, 1189, 1104, 969, and 797. UV-Vis bands at 409, 488, 680, 788, 975 nm. Pure colored single crystals were isolated (after removal of mother liquor using a syringe; washed with cold *n*-hexane), which were ground to form powders required for all other

characterizations (EPR, UV, and CV). Elemental analysis for $C_{81}H_{102}FeN_6S_6Se_3$ [calculated] (%): C 59.21 [59.15], H 6.23 [6.25], N 5.01 [5.11], matching with [Fe(SS-NHC=Se)$_3$].

***A similar reaction, which produced complex **1**, did not lead to the isolation of complex **2** when [(THF)$_2$Li(SS-NHC=Se)] was employed as a ligand. The exact role of KOH for the synthesis of **2** is not known.

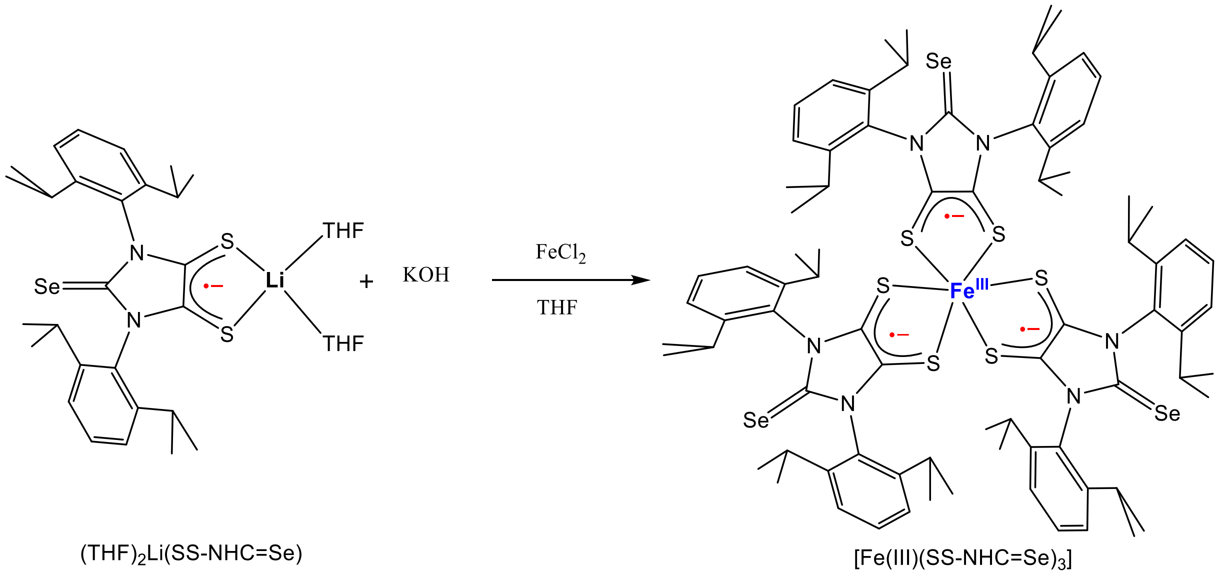

Scheme S2. Synthesis of complex [Fe(III)(SS-NHC=Se)$_3$] (**2**).

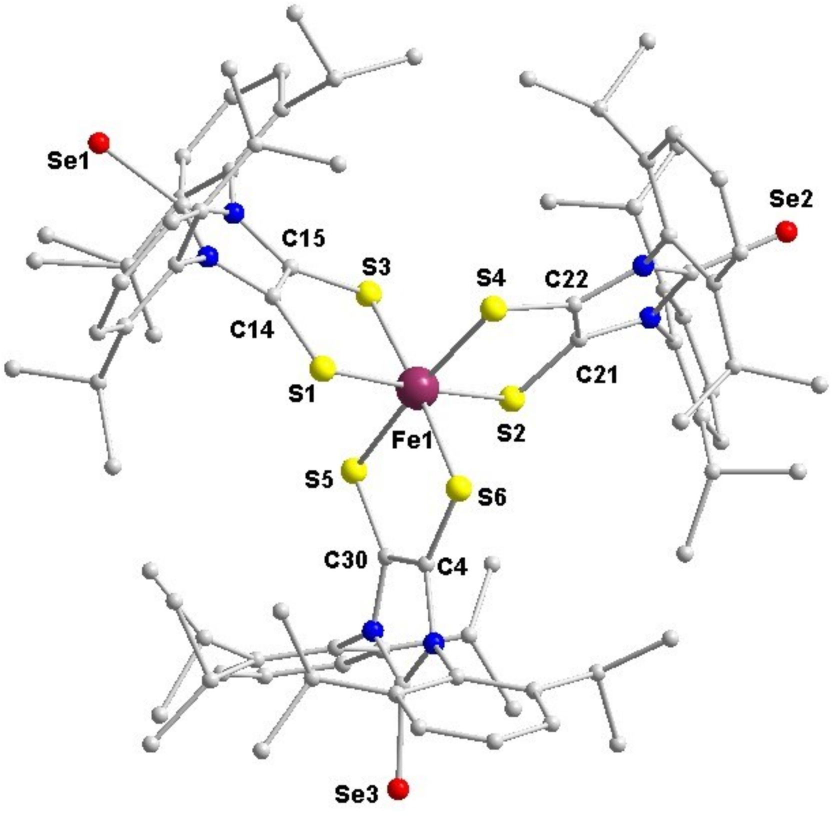

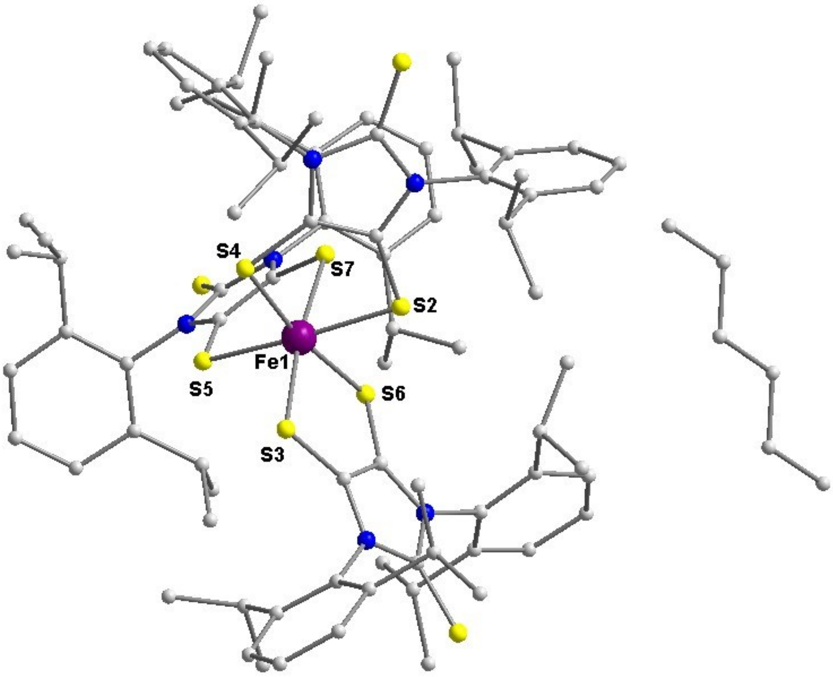

Figure S1. Molecular structure of complex [Fe(III)(SS-NHC=Se)$_3$] (**2**) (top) and [Fe(III)(SS-NHC=S)$_3$]·0.25*n*-hexane (bottom). All the H atoms were omitted for clarity. Red, Se; blue, N; off white C.

Alternating Synthetic routes of complex **1**: [Fe(III)(SS-NHC=S)$_3$]· 0.25*n*-hexane:

The synthesis was started with the lithium salt of the dithiolene radical anion, [(THF)$_2$Li(SS-NHC=S)], which was isolated as a dark purple solid (253 mg, 0.4 mmol). This solid was dissolved in 10 mL of dry tetrahydrofuran (THF) in a Schlenk flask, resulting in a vivid dark purple solution after 10 min of stirring. Separately, 51.5 mg (0.4 mmol) of anhydrous $FeCl_2$ was dissolved in 5 mL of THF. The $FeCl_2$ solution was then added to the dark purple solution, causing a rapid color change to brown, indicating the initiation of the reaction. The mixture was stirred for 30 min to ensure complete reaction. Next, 45 mg (0.8 mmol) of KOH was added at low temperature, and the reaction was stirred overnight. The color changed from brown to red-purple. Afterward, the solvent was removed, and the product was extracted with 12 mL of *n*-hexane, followed by crystallization. Needle-like crystals of [Fe(III)(SS-NHC=S)$_3$]·*n*-hexane (**1**·0.25*n*-hexane) were obtained in a 45% yield. Pure single crystals were isolated by removing the mother liquor with a syringe and washing with cold *n*-hexane. Alternatively, the same product can be obtained by replacing KOH with KC_8. The reaction proceeds similarly, with 30 mg (0.2 mmol) KC_8 acting as the reducing agent (yield 42%).

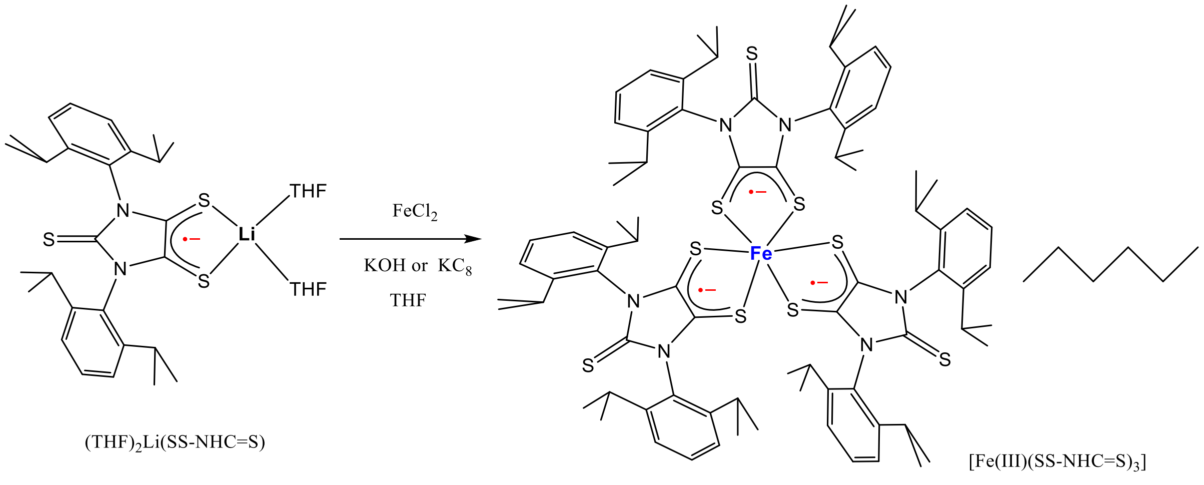

Scheme S3. Synthesis of complex [Fe(III)(SS-NHC=S)$_3$]·0.25*n*-hexane.

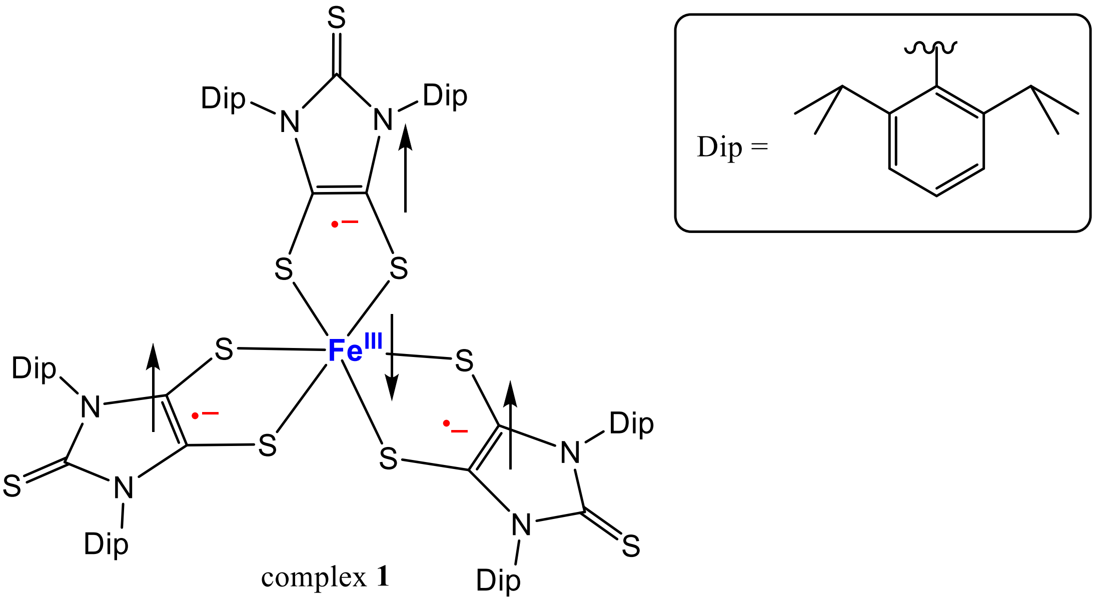

low spin Fe(III); S = 1/2
each radical; S = 1/2

S = (1/2 + 1/2 + 1/2) - 1/2 = 1

S = 1

Figure S2. Spin structure of complex **1**.

3 Magnetic properties calculations

A free radical electron with spin quantum number $S = {}^{1}\!/_{2}$ has a paramagnetic moment of $\mu_{1/2} = g_s \cdot \sqrt{S(S+1)}\mu_B = 2 \cdot \sqrt{1/2 \cdot 3/2}\mu_B \approx 1.73\ \mu_B$, with the electron g-factor $g_s \cong 2$ and the Bohr magneton μ_B. A free Fe^{3+} ion with 5 *d*-electrons in high-spin (HS) configuration $(t_g)^3(e_g)^2$ with spin quantum number $S = {}^{5}\!/_{2}$ has a paramagnetic moment of $\mu_{5/2} = g_s \cdot \sqrt{S(S+1)}\mu_B = 2 \cdot \sqrt{5/2 \cdot 7/2}\mu_B \approx 5.92\ \mu_B$, and a Fe^{3+} ion with 5 *d*-electrons in low-spin (LS) configuration $(t_g)^5(e_g)^0$ with spin quantum number $S = {}^{1}\!/_{2}$ has a paramagnetic moment of $\mu_{1/2} = g_s \cdot \sqrt{S(S+1)}\mu_B = 2 \cdot \sqrt{1/2 \cdot 3/2}\mu_B \approx 1.73\ \mu_B$. The correspondend molar Curie-constant C_c can be calculated from the 8aramagnetic moments according to: $C_C = \mu_{tot}^2 \cdot N_A/(3k_B)$. Three free radical electrons together with a free HS Fe^{3+} would result in a total Curie constant of $C_C =$

$\sqrt{3\mu_{1/2}^2 + 1\mu_{5/2}^2 \cdot \frac{N_A}{3k_B}}$ = 5.50 cm^3 K mol^{-1} and that of three free radical electrons together with a free LS Fe^{3+} in $C_C' = \sqrt{4\mu_{1/2}^2 \cdot \frac{N_A}{3k_B}}$ = 1.50 cm^3 K mol^{-1}.

Hamiltonian used for PHI fit to experimental DC magnetometry data:

$$\hat{H} = \hat{H}_{\mathrm{SO}} + \hat{H}_{\mathrm{CF}} + +\hat{H}_{\mathrm{EX}} + \hat{H}_{\mathrm{ZEE}} \qquad \text{(S 1)}$$

with Hamilton operator for spin-orbit coupling (SO), crystal-field interaction (CF), exchange interaction (EX), and Zeeman effect (ZEE).

$$\hat{H}_{\mathrm{SO}} = \sum_{i=1}^{N} \lambda_i \left(\sigma_{\mathrm{SO},i} \vec{\hat{L}}_i \cdot \vec{\hat{S}}_i\right)$$

with spin-orbit coupling constants λ_i, orbital-reduction parameters $\sigma_{SO,i}$, vector operator of total orbital momentum $\vec{\hat{L}}_i$, vector operator of total spin orbital momentum $\vec{\hat{S}}_i$.

$$\hat{H}_{\mathrm{CF}} = \sum_{i=1}^{N} \sum_{k=2,4,6} \sum_{q=-k}^{k} \sigma_i^k B_{ki}^q \theta_k \, \hat{O}_{ki}^q$$

with crystal-field parameters B_{ki}^q ($A_{ki}^q \langle r^k \rangle_i$ in Steven's notation), operator equivalent factors θ_k and operator equivalents $\hat{O}_{ki}^q$. In this work, the operator equivalent factors are included into the CFPs by setting $B_{ki}^q = B'^q_{ki} \theta_k$.

$$\hat{H}_{\mathrm{EX}} = -2 \sum_{i \neq j}^{i,j \in N} \vec{\hat{S}}_i \cdot J_{iso} \cdot \vec{\hat{S}}_j$$

with scalar isotropic exchange parameter J_{iso}.

$$\hat{H}_{\mathrm{ZEE}} = \mu_{\mathrm{B}} \sum_{i=1}^{N} \left(\sigma_i \vec{\hat{L}}_i \cdot \bar{\bar{I}} + \vec{\hat{S}}_i \cdot \bar{\bar{g}}_i\right) \cdot \vec{B}$$

with Bohr magneton μ_B, identity matrix $\bar{\bar{I}}$, g-tensor $\bar{\bar{g}}_i$ and magnetic induction $\vec{B}$.

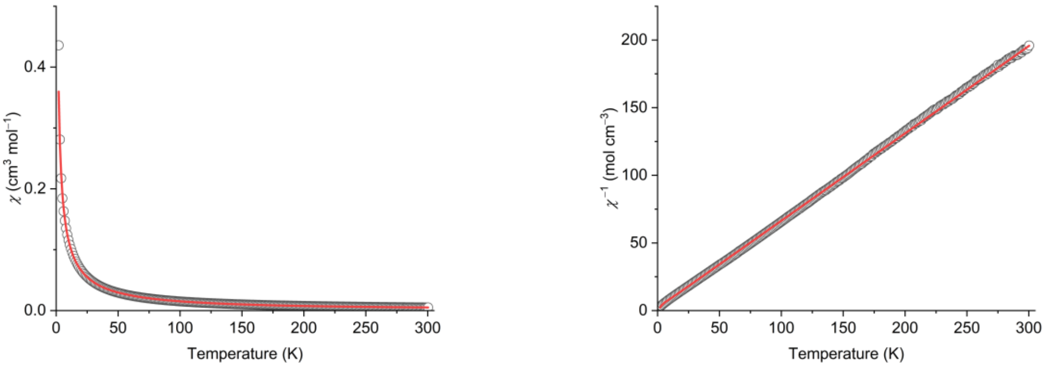

Figure S3. χ vs T (left), and $1/\chi$ vs T plots (right) of complex **1**. Open circles represent measured values and red lines the simulation of the magnetic model as outlined in the main text.

4 Single crystal X-ray diffraction:

Table S1. Crystallographic data for the complex [Fe(SS-NHC=S)$_3$]·NHC=S (**1**·NHC=S) at 100 K (left), 298 K (middle, where the crystal structure was obtained from one equivalent of $FeCl_3$ and three equivalents of the radical ligand), and [Fe(III)(SS-NHC=S)$_3$]·0.25n-hexane (1·0.25n-hexane) at room temperature (right)

**CCDC Deposit**	**2345820**	**2451970**	**2402302**
**complex**	**1**·NHC=S	**1**·NHC=S	**1**·0.25*n*-hexane
Empirical formula	$C_{108}H_{137.71}FeN_8S_{10}$	$C_{108}H_{138}FeN_8S_{10}$	$C_{165}H_{211}Fe_2N_{12}S_{18}$
Formula weight	1924.42	1924.71	3051.23
Temperature (K)	**100(2)**	**298(2)**	106(2)
Wavelength (Å)	0.71073	0.71073	0.71073
Crystal system	Orthorhombic	Orthorhombic	Triclinic
Space group	$P2_12_12_1$	$P\ 2_12_12_1$	P -1
Unit cell dimensions	a = 17.398(2) Å<br>b = 17.604(2) Å<br>c = 35.031(5) Å<br>α = 90°<br>β = 90°	a = 17.5779(6) Å<br>b = 17.7124(6) Å<br>c = 35.7819(12) Å<br>α = 90°	a = 15.457(2) Å<br>b = 22.945(3) Å<br>c = 26.659(3) Å<br>α = 101.015(5)°

	γ = 90°	β = 90°<br>γ = 90°	β = 91.369(5)°<br>γ = 92.541(6)°
Volume (Å^3)	10729(2)	11140.6(7)	9267(2)
Z	4	4	2
Density (Mg/m^3)	1.191	1.148	1.094
Absorption coefficient (mm^{-1})	0.384	0.37	0.406
F (000)	4111	4112	3250
Crystal size (mm^3)	0.11x0.15x0.07	0.324 x 0.049 x 0.041	0.226 × 0.201 × 0.104
Theta range for data collection (°)	2.907 to 25.027°	3.98 to 53.46	1.510 to 25.027
Index ranges	-20<=h<=20,<br>-20<=k<=20,<br>-41<=l<=41	-20<=h<=22,<br>-22<=k<=22,<br>-45<=l<=45	-18<=h<=18,<br>-27<=k<=27,<br>-31<=l<=31
R (int)	0.0980	0.163	0.2679
Reflections collected	235215	144578	334914
Independent reflections	18931	23027	32747
Completeness to theta = 25.027°	99.7 %	96.4 %	100.0 %
Max. and min. transmission	Full-matrix least-squares on F^2	0.7170 and 0.5965	0.7454 and 0.6405
Number of Restraints	36	216	12
Number of Parameters	1197	1352	1834
Refinement method	R_1 = 0.0481,<br>wR_2 =0.1155	Full-matrix least-squares on F^2	Full-matrix least-squares on F^2
Final R indices [I>2sigma(I)]	R_1 = 0.0548,	R_1 = 0.0711,	R_1 = 0.0736,

	wR_2 = 0.1198	wR_2 = 0.1319	wR_2 = 0.1975
R indices (all data)	1.132	R_1 = 0.2162,<br>wR_2 = 0.1820	R_1 = 0.1773,<br>wR_2 = 0.2964
GooF	0.479 and -0.398	0.898	0.841
Largest diff. peak and hole [e·Å^{-3}]	0.384	0.40 and -0.28	0.537 and -0.860

Table S2. Selected bond lengths [Å] bond angles [°] of complex [Fe(SS-NHC=S)$_3$]·NHC=S (**1**·NHC=S) at 100 K and 298 K.

Bond length	At 100 K	At 298 K
Fe1—S3	2.2498 (13)	2.251(3)
Fe1—S4	2.2658 (13)	2.266(3)
Fe1—S5	2.2783 (13)	2.272(3)
Fe1—S2	2.2954 (13)	2.300(3)
Fe1—S7	2.3011 (13)	2.306(3)
Fe1—S6	2.3020 (13)	2.307(3)
S1—C7	1.641 (5)	
S2—C8	1.683 (5)	
S3—C20	1.684 (5)	
S4—C9	1.685 (5)	
S5—C29	1.690 (5)	
S6—C22	1.694 (5)	
S7—C31	1.686 (5)	
S8—C21	1.641 (5)	
S9—C30	1.647 (5)	
S10—C90	1.669 (5)	
N1—C9	1.380 (6)	
N1—C7	1.394 (6)	
N1—C1	1.447 (6)	
N2—C7	1.380 (6)	
N2—C8	1.380 (6)	
N2—C14	1.452 (6)	
N3—C20	1.378 (6)	
N3—C21	1.390 (6)	
N3—C32	1.449 (6)	
N4—C22	1.367 (6)	
N4—C21	1.390 (7)	
N4—C38	1.439 (6)	
N5—C29	1.377 (6)	
N5—C30	1.378 (6)	

N5—C46	1.442 (6)	
N6—C31	1.377 (6)	
N6—C30	1.386 (6)	
N6—C52	1.440 (6)	
N7—C90	1.377 (6)	
N7—C88	1.386 (7)	
N7—C82	1.447 (7)	
N8—C90	1.373 (7)	
N8—C89	1.386 (7)	
N8—C95	1.437 (7)	
C1—C6	1.395 (7)	
C1—C2	1.403 (7)	
C2—C3	1.379 (8)	
C2—C10	1.529 (8)	
C3—C4	1.381 (8)	
C4—C5	1.387 (8)	
C5—C6	1.398 (7)	
C6—C12	1.516 (7)	
C8—C9	1.405 (6)	
C10—C11'	1.38 (4)	
C10—C23	1.513 (8)	
C10—C11	1.63 (2)	
C12—C24	1.525 (7)	
C12—C13	1.541 (7)	
C14—C15	1.380 (8)	
C14—C19	1.406 (7)	
C15—C16	1.407 (8)	
C15—C25	1.526 (8)	
C16—C17	1.386 (9)	
C17—C18	1.377 (9)	
C18—C19	1.399 (7)	
C19—C27	1.520 (8)	

C20—C22	1.405 (7)	
C25—C26	1.522 (9)	
C25—C44	1.532 (9)	
C27—C28	1.525 (8)	
C27—C45	1.530 (7)	
C29—C31	1.403 (7)	
C32—C37	1.392 (7)	
C32—C33	1.398 (7)	
C33—C34	1.389 (8)	
S3—Fe1—S4	90.79 (5)	
S3—Fe1—S5	90.18 (5)	
S4—Fe1—S5	90.88 (5)	
S3—Fe1—S2	172.82 (5)	
S3—Fe1—S4	90.79 (5)	
S4—Fe1—S2	92.29 (5)	
S5—Fe1—S2	96.25 (5)	
S3—Fe1—S7	95.93 (5)	
S4—Fe1—S7	172.40 (5)	
S5—Fe1—S7	92.63 (4)	
S2—Fe1—S7	80.63 (4)	
S3—Fe1—S6	92.47 (5)	
S4—Fe1—S6	96.76 (5)	
S5—Fe1—S6	171.88 (5)	
S2—Fe1—S6	80.74 (5)	
S7—Fe1—S6	79.46 (4)	

Table S3. Selected bond lengths [Å] for the complex [Fe(III)(SS-NHC=S)$_3$]·0.25*n*-hexane:

Fe(1)-S(3)	2.2611(19)
Fe(1)-S(5)	2.2643(19)
Fe(1)-S(4)	2.285(2)
Fe(1)-S(2)	2.2858(19)
Fe(1)-S(6)	2.294(2)
Fe(1)-S(7)	2.3058(19)
Fe(2)-S(12)	2.262(2)
Fe(2)-S(13)	2.268(2)
Fe(2)-S(11)	2.271(2)
Fe(2)-S(14)	2.2730(19)
Fe(2)-S(15)	2.278(2)
Fe(2)-S(16)	2.291(2)
S(1)-C(9)	1.637(7)
S(2)-C(10)	1.682(7)
S(3)-C(2)	1.680(7)
S(4)-C(1)	1.685(7)
S(5)-C(29)	1.697(7)
S(6)-C(22)	1.683(7)
S(7)-C(31)	1.677(7)
S(8)-C(21)	1.639(7)
S(9)-C(30)	1.635(7)
S(10)-C(88)	1.628(7)

S(11)-C(89)	1.674(7)
S(12)-C(101)	1.685(7)
S(13)-C(90)	1.685(7)
S(14)-C(110)	1.682(7)
S(15)-C(112)	1.674(7)
S(16)-C(103)	1.665(7)
S(17)-C(102)	1.628(8)
S(18)-C(111)	1.631(7)
C(2)-C(22)	1.404(9)
C(1)-C(10)	1.388(9)
C(29)-C(31)	1.384(10)
C(89)-C(90)	1.407(9)
C(101)-C(103)	1.404(10)
C(110)-C(112)	1.407(9)
S(3)-Fe(1)-S(5)	90.24(7)
S(3)-Fe(1)-S(4)	94.32(7)
S(5)-Fe(1)-S(4)	91.70(7)
S(3)-Fe(1)-S(2)	97.21(7)
S(5)-Fe(1)-S(2)	171.46(8)
S(4)-Fe(1)-S(2)	91.89(7)
S(3)-Fe(1)-S(6)	91.76(7)
S(5)-Fe(1)-S(6)	94.93(7)
S(4)-Fe(1)-S(6)	170.98(7)

S(2)-Fe(1)-S(6)	80.73(7)
S(3)-Fe(1)-S(7)	170.71(8)
S(5)-Fe(1)-S(7)	92.01(7)
S(4)-Fe(1)-S(7)	94.62(7)
S(2)-Fe(1)-S(7)	79.98(7)
S(6)-Fe(1)-S(7)	79.07(7)
S(12)-Fe(2)-S(13)	87.50(7)
S(12)-Fe(2)-S(11)	174.88(8)
S(13)-Fe(2)-S(11)	92.83(7)
S(12)-Fe(2)-S(14)	88.02(7)
S(13)-Fe(2)-S(14)	87.28(7)
S(11)-Fe(2)-S(14)	97.10(7)
S(12)-Fe(2)-S(15)	96.90(7)
S(13)-Fe(2)-S(15)	175.60(8)
S(11)-Fe(2)-S(15)	82.82(7)
S(14)-Fe(2)-S(15)	92.53(7)
S(12)-Fe(2)-S(16)	92.58(7)
S(13)-Fe(2)-S(16)	98.22(7)
S(11)-Fe(2)-S(16)	82.31(7)
S(14)-Fe(2)-S(16)	174.49(8)
S(15)-Fe(2)-S(16)	81.95(7)
C(10)-S(2)-Fe(1)	97.2(2)
C(2)-S(3)-Fe(1)	99.5(2)

C(1)-S(4)-Fe(1)	97.8(2)
C(29)-S(5)-Fe(1)	98.1(2)
C(22)-S(6)-Fe(1)	99.3(2)
C(31)-S(7)-Fe(1)	97.4(2)
C(89)-S(11)-Fe(2)	99.4(2)
C(101)-S(12)-Fe(2)	98.8(2)
C(90)-S(13)-Fe(2)	99.4(3)
C(110)-S(14)-Fe(2)	99.4(2)
C(112)-S(15)-Fe(2)	99.0(3)
C(103)-S(16)-Fe(2)	98.8(3)

Table S4. Crystallographic details of the complex [Fe(III)(SS-NHC=Se)$_3$] (**2**) [E = Se].

**CCDC Deposit**	**2404919**
Empirical formula	$C_{81}H_{102}FeN_6S_6Se_3$
Formula weight	1644.77
Temperature (K)	200 (2)
Wavelength (Å)	0.71073
Crystal system	Monoclinic
Space group	C*2/c*
Unit cell dimensions	*a* = 16.5140(11) Å<br>*b* = 25.9476(16) Å<br>*c* = 44.204(3) Å<br>*α* = 90°<br>*β* = 98.831(3)°

	γ = 90°
Volume (Å^3)	18717(2)
Z	8
Density (Mg/m^3)	1.167
Absorption coefficient (mm^{-1})	1.501
F (000)	6832
Crystal size (mm^3)	0.327 x 0.212 x 0.120
Theta range for data collection (°)	2.103 to 26.400
Index ranges	-20<=h<=20,<br>-32<=k<=32,<br>-55<=l<=55
R (int)	0.0726
Reflections collected	143845
Independent reflections	19144
Completeness to theta = 25.242°	99.9 %
Max. and min. transmission	0.7454 and 0.6421
Number of Restraints	36
Number of Parameters	938
Refinement method	Full-matrix least-squares on F^2
Final *R* indices [*I*>2sigma(*I*)]	R_1 = 0.0565,<br>wR_2 = 0.1655
R indices (all data)	R_1 = 0.0771,<br>wR_2 = 0.1759
GooF	1.204
Largest diff. peak and hole [e·Å^{-3}]	0.926 and -0.544

Table S5. Selected bond lengths [Å] of the complex (**2**):

Fe(1)-S(1)	2.2687(10)	S(1)-C(14)	1.686(3)
Fe(1)-S(2)	2.2736(10)	S(2)-C(21)	1.683(3)
Fe(1)-S(5)	2.2794(10)	S(3)-C(15)	1.679(4)
Fe(1)-S(3)	2.2808(10)	S(4)-C(22)	1.687(3)
Fe(1)-S(6)	2.2895(10)	S(5)-C(30)	1.694(3)
Fe(1)-S(4)	2.2903(10)	S(6)-C(4)	1.686(3)
Se(1)-C(16)	1.799(4)	C(4)-C(30)	1.392(5)
Se(2)-C(1)	1.807(4)	C(14)-C(15)	1.393(5)
Se(3)-C(5)	1.809(3)	C(21)-C(22)	1.391(5)

Table S6. Selected bond angles [°] of the complex (**2**):

S(1)-Fe(1)-S(2)	176.11(4)	S(2)-Fe(1)-S(4)	92.28(3)
S(1)-Fe(1)-S(5)	99.38(4)	S(5)-Fe(1)-S(4)	174.05(4)
S(2)-Fe(1)-S(5)	82.09(3)	S(3)-Fe(1)-S(4)	97.52(4)
S(1)-Fe(1)-S(3)	92.12(4)	S(6)-Fe(1)-S(4)	86.58(4)
S(2)-Fe(1)-S(3)	84.45(4)	C(14)-S(1)-Fe(1)	99.98(13)
S(5)-Fe(1)-S(3)	83.93(4)	C(21)-S(2)-Fe(1)	99.24(12)
S(1)-Fe(1)-S(6)	86.58(4)	C(15)-S(3)-Fe(1)	99.77(13)
S(2)-Fe(1)-S(6)	96.97(4)	C(22)-S(4)-Fe(1)	99.66(12)
S(5)-Fe(1)-S(6)	92.15(3)	C(30)-S(5)-Fe(1)	99.20(12)
S(3)-Fe(1)-S(6)	175.62(4)	C(4)-S(6)-Fe(1)	99.46(12)
S(1)-Fe(1)-S(4)	86.35(3)		

5. EPR measurements of complex 1

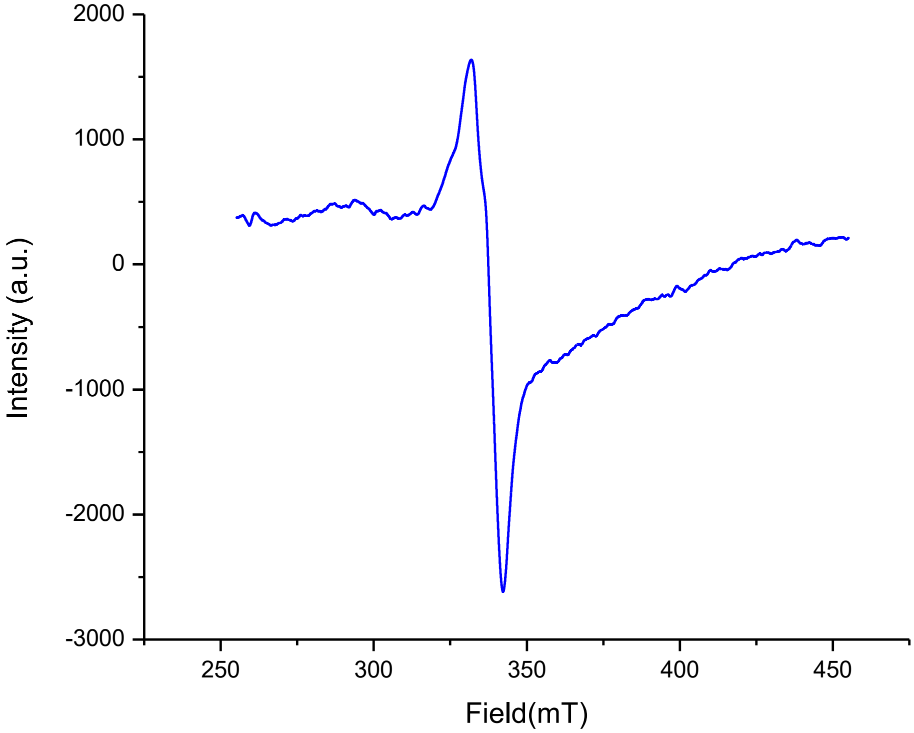

Figure S4. X-band EPR spectrum of complex [Fe(SS-NHC=S)$_3$]·NHC=S (**1**·NHC=S) in solid-state at rt.

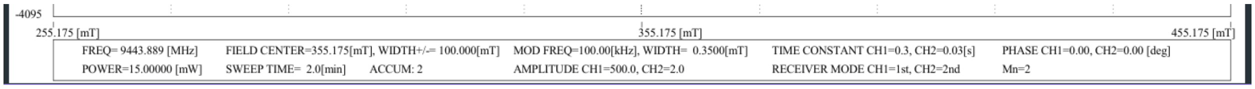

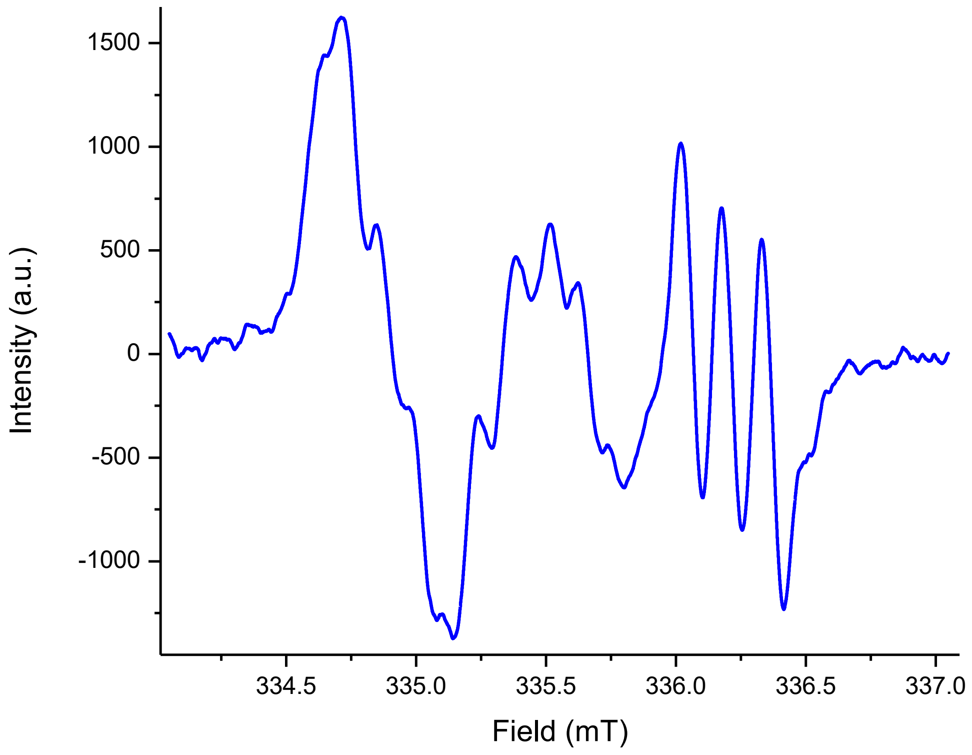

Figure S5. X-band EPR spectrum of complex [Fe(SS-NHC=S)$_3$]·NHC=S (**1**·NHC=S) in THF solution at room temperature. Frequency = 9.4486 GHz, Centre of Field = 3355.48 G, Mod frequency = 100 KHz, Width = 0.5 G. g = 2.01577.

FREQ= 9448.623 [MHz]	FIELD CENTER=335.548[mT], WIDTH+/-= 1.500[mT]	MOD FREQ=100.00[kHz], WIDTH= 0.0500[mT]	TIME CONSTANT CH1=1, CH2=0.03[s]	PHASE CH1=0.00, CH2=0.00 [deg]
POWER=25.00000 [mW]	SWEEP TIME= 4.0[min] ACCUM: 3	AMPLITUDE CH1=5000.0, CH2=2.0	RECEIVER MODE CH1=1st, CH2=2nd	Mn=15

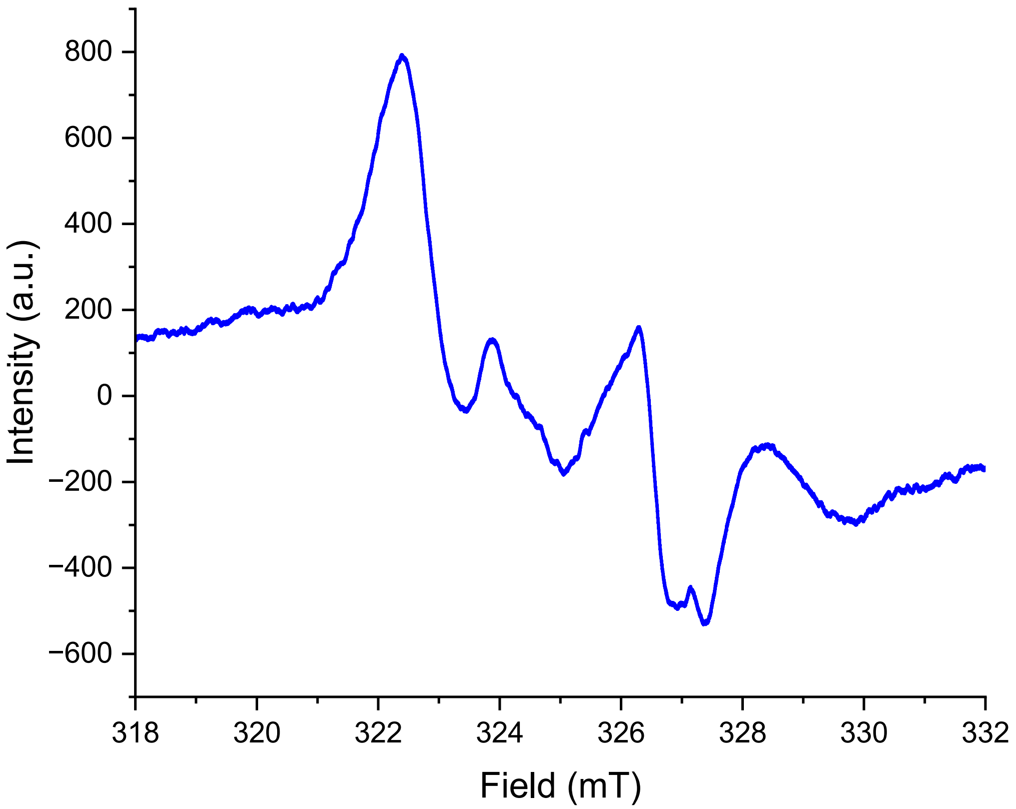

Figure S6. X-band EPR spectrum of complex [Fe(SS-NHC=S)$_3$]·NHC=S (**1**·NHC=S) in frozen THF at 77 K. Frequency = 9.1719 GHz, Centre of Field = 3256.12 G, Mod frequency = 100 KHz, Width = 0.5 G.

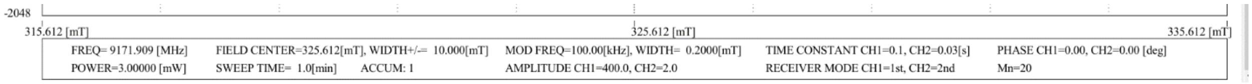

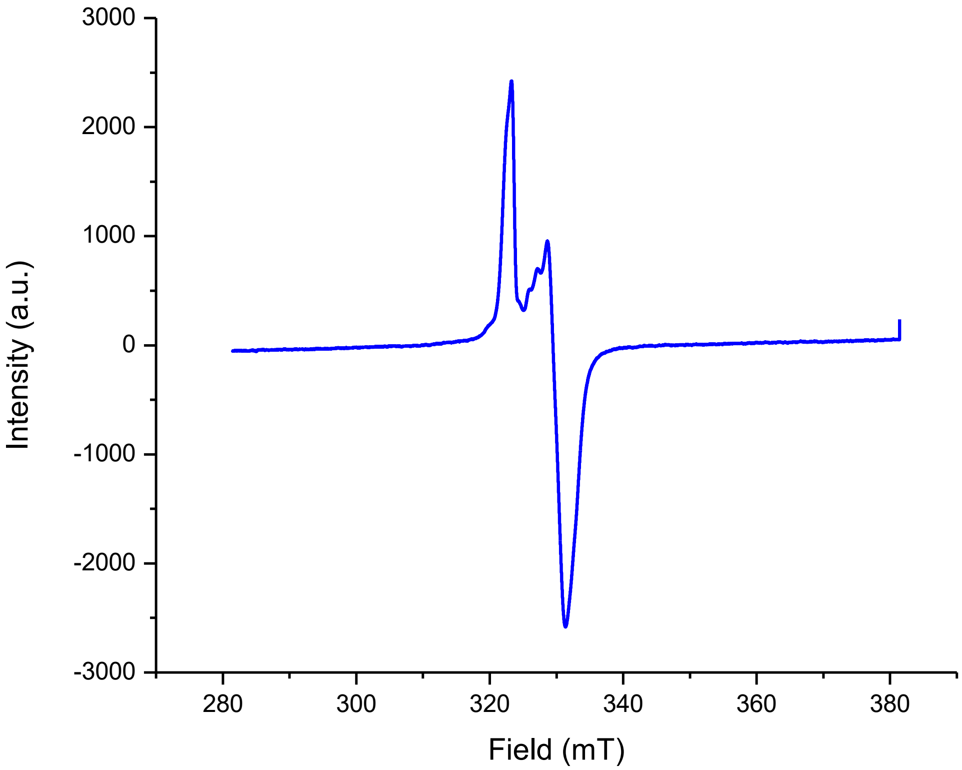

Figure S7. Experimental EPR spectrum of complex [Fe(SS-NHC=S)$_3$]·NHC=S (**1**·NHC=S) in solid-state at 77 K.

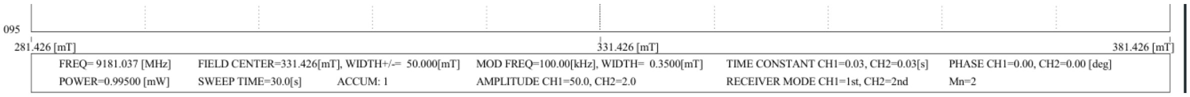

6. IR spectra

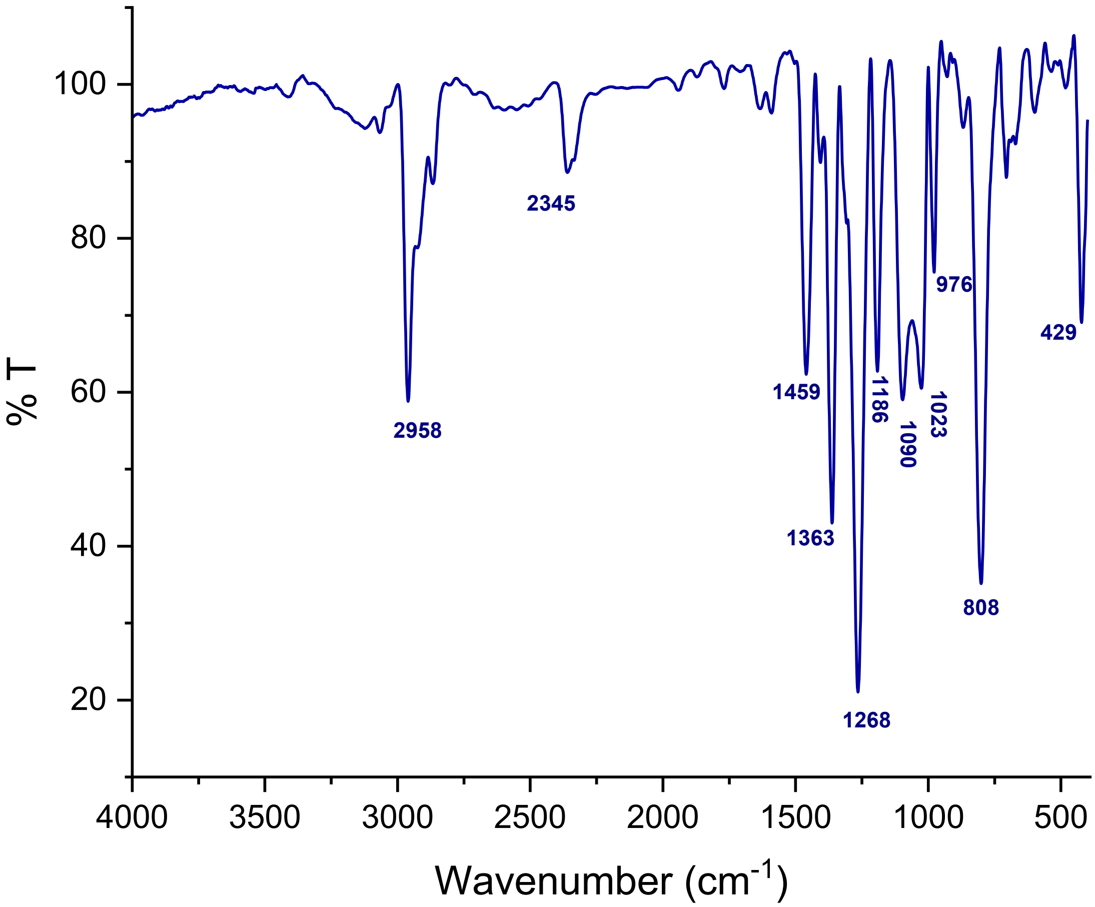

100
80
60
40
20
% T
2958
2345
1459
1363
1268
1186
1090
1023
976
808
429
4000
3500
3000
2500
2000
1500
1000
500
Wavenumber (cm-1)

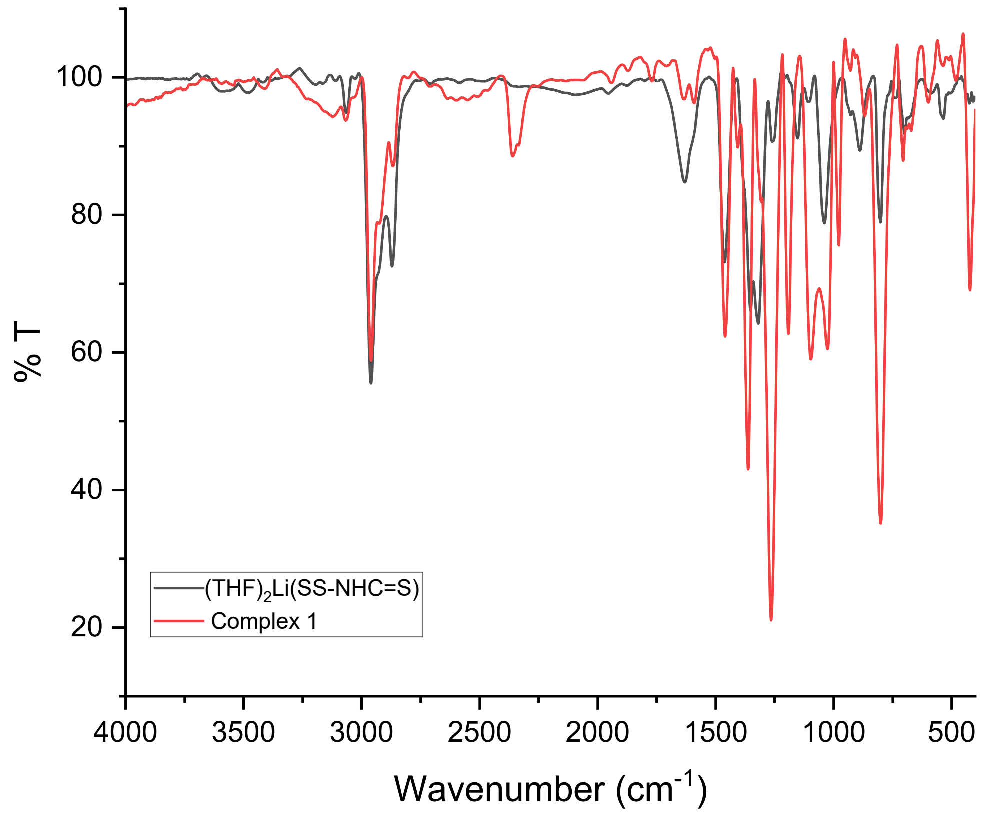

Figure S8. IR spectrum of complex [Fe(SS-NHC=S)$_3$]·NHC=S (**1**·NHC=S) in KBr (top). IR spectra of [Fe(SS-NHC=S)$_3$]·NHC=S (**1**·NHC=S) and (THF)$_2$Li(SS-NHC=S) in KBr (bottom). Sample was prepared inside the glove box. Water was removed from KBr by heating at 250 ºC under vacuum prior sample preparation.

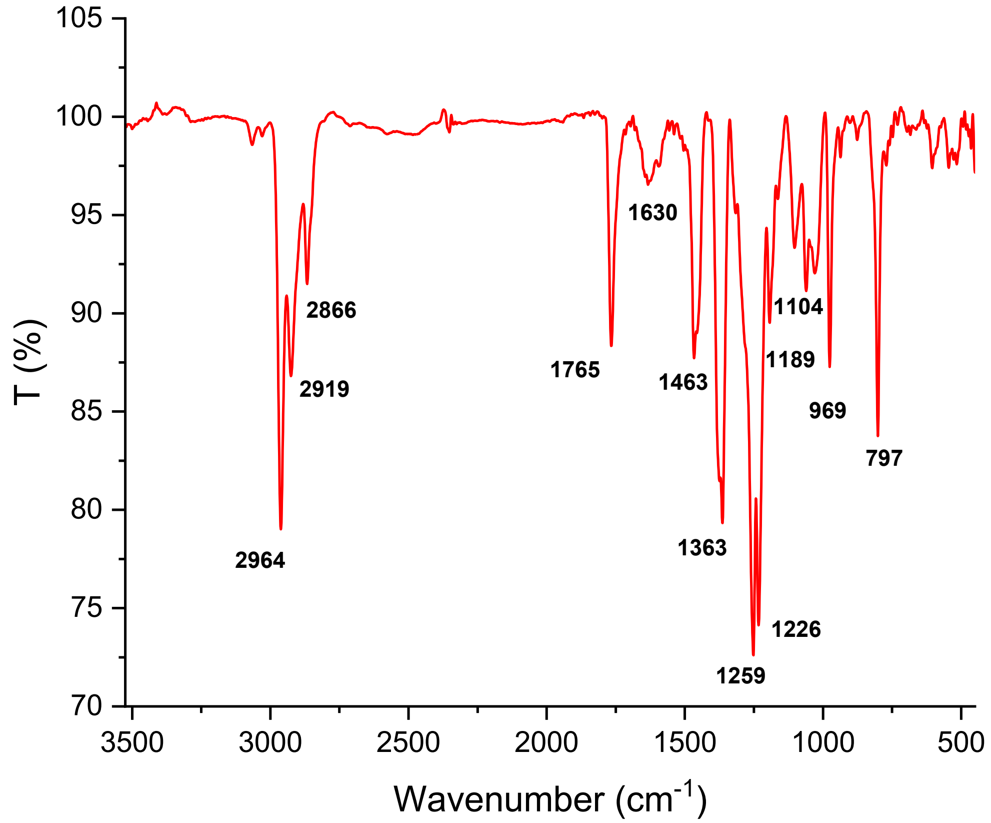

Figure S9. FT-IR spectrum of [Fe(III)(SS-NHC=Se)$_3$] (**2**) with dry KBr. Sample was prepared inside the glove box. Water was removed from KBr by heating at 250 ºC under vacuum prior sample preparation.

7. UV-VIS-NIR measurements

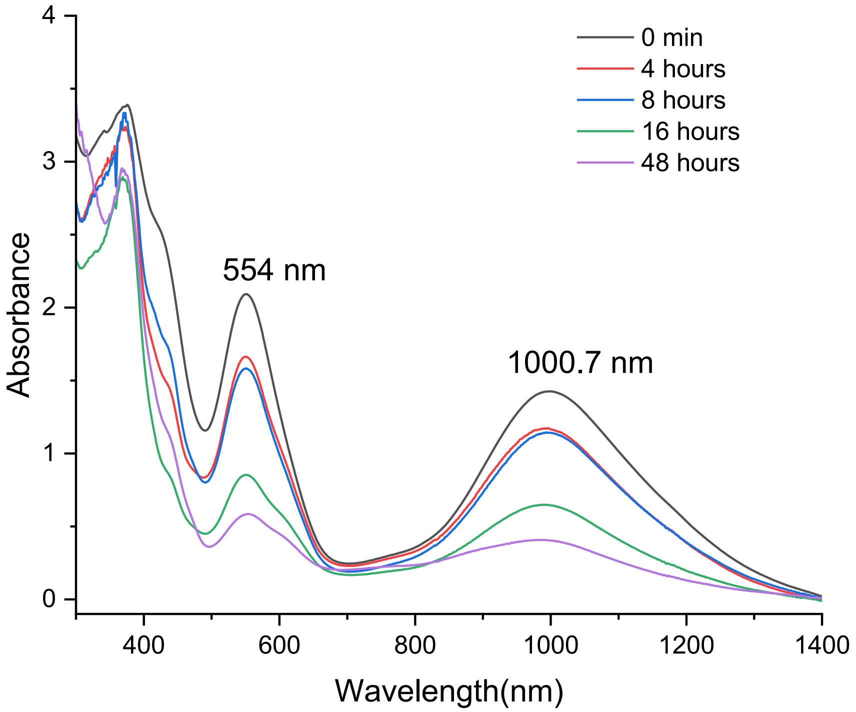

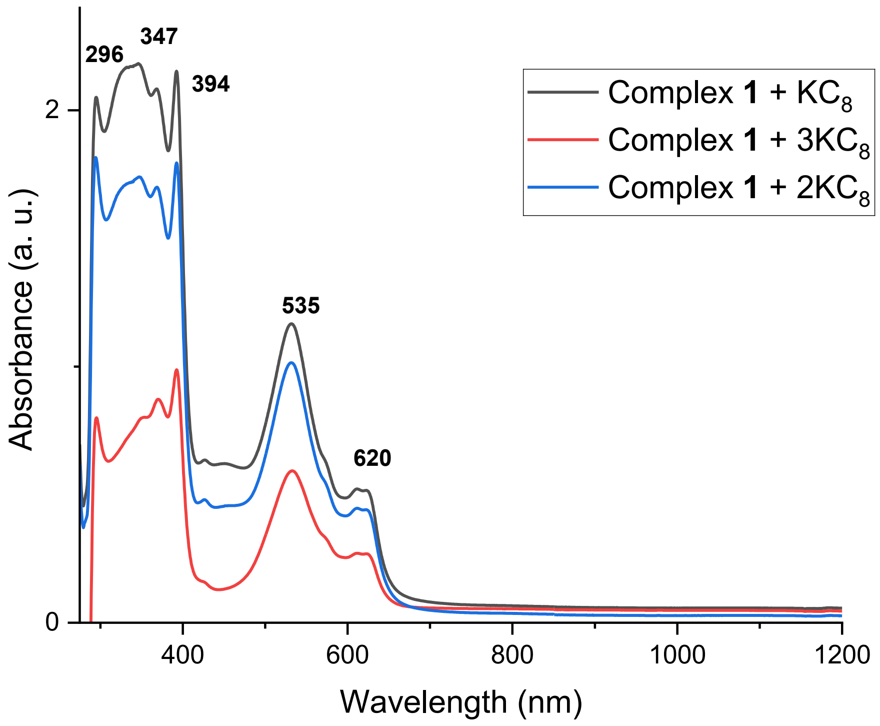

Figure S10. UV-vis-NIR spectrum of the complex (**1**·NHC=S) in THF solution. Crystals of (**1**·NHC=S) were exposed to air for the indicated times and then transferred back into an inert atmosphere box for analysis. Spectra were recorded to monitor decomposition over time. Approximately 50% decomposition occurs after ~15 hours of air exposure (top). Bottom: UV-vis-NIR spectra of the THF solution of 1·NHC=S after treatment with 1, 2, and 3 equivalents of KC_8, showing spectral changes upon reduction.

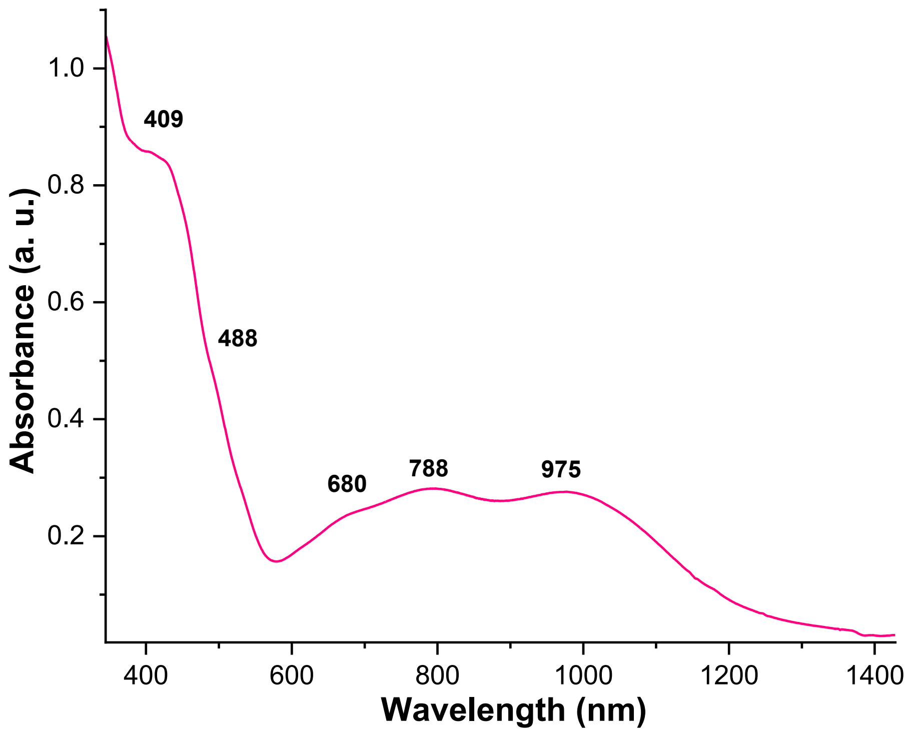

Figure S11. UV-VIS spectrum of complex [Fe(III)(SS-NHC=Se)$_3$] (**2**) in THF solution.

8. CV measurements

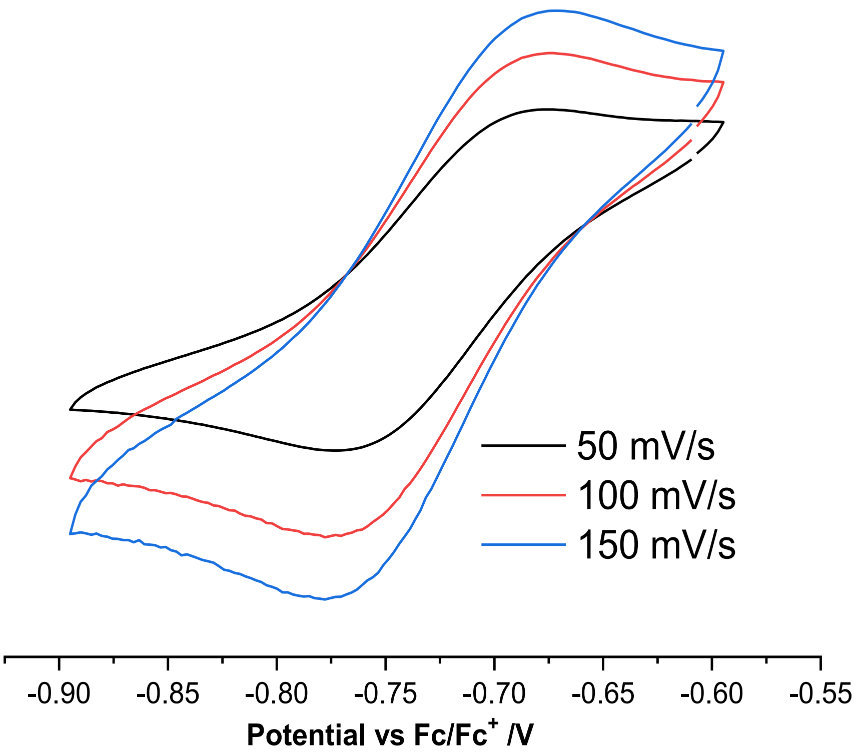

Figure S12. The cyclic voltammogram of complex [Fe(SS-NHC=S)$_3$]·NHC=S (**1**·NHC=S) in THF at rt containing 0.1 M [*n*-Bu$_4$N][PF$_6$] as supporting electrolyte, at scan rates of 50/100/150 mV s^{-1}. Reduction (left) and oxidation (right). Glassy Carbon as WE (Working electrode), Platinum Wire as CE (Counter Electrode). Ag Wire as RE (Reference Electrode). The potential (V) is plotted again the standard reduction potential of Cp_2Fe^+/Cp_2Fe. The voltage ranges from -0.22 V to -0.52 V.

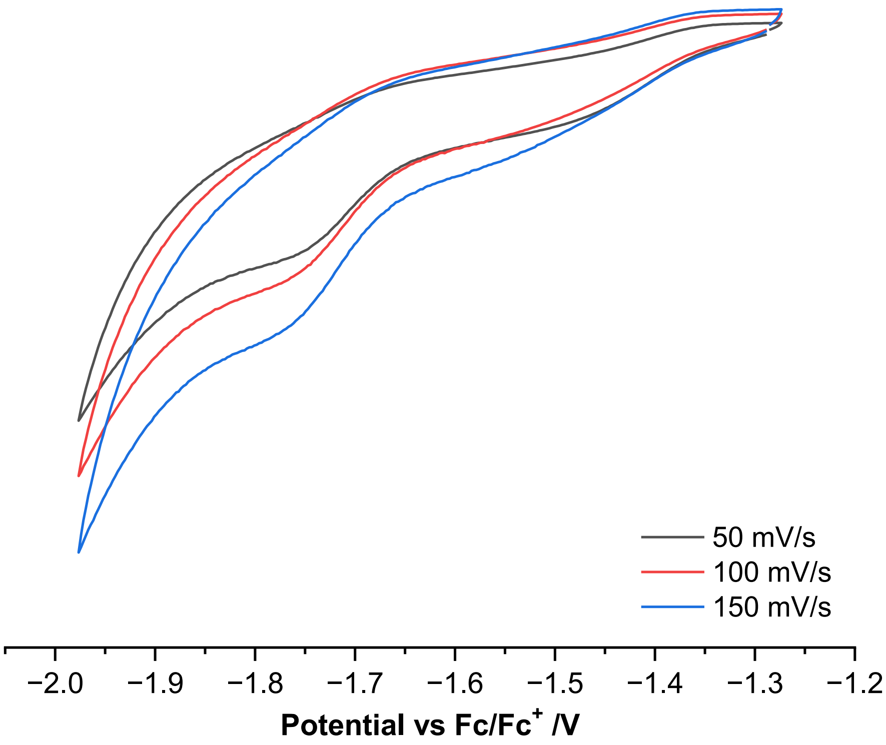

Figure S13. The cyclic voltammogram of complex $[Fe(SS\text{-}NHC{=}S)_3]\cdot NHC{=}S$ (**1**·NHC=S) in THF at rt containing 0.1 M $[n\text{-}Bu_4N][PF_6]$ as supporting electrolyte, at scan rates of 50/100/150 mV s^{-1}. Reduction (left) and oxidation (right). Glassy Carbon as WE (Working electrode), Platinum Wire as CE (Counter Electrode). Ag Wire as RE (Reference Electrode). The potential (V) is plotted again the standard reduction potential of Cp_2Fe^{+}/Cp_2Fe. The voltage ranges from -0.9 V to -1.60 V.

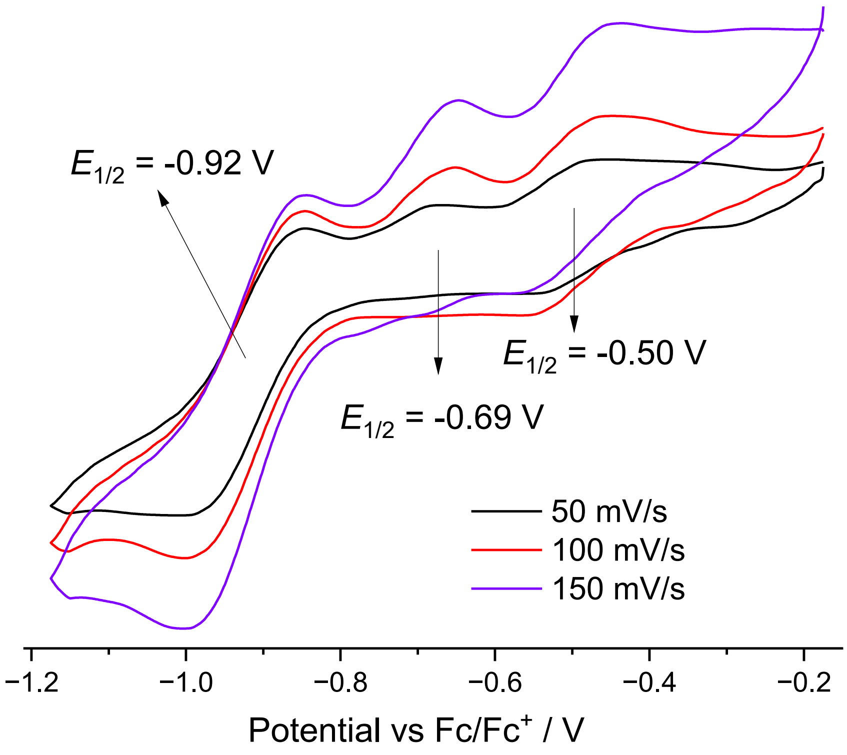

Figure S14. Cyclic voltammogram of complex [Fe(III)(SS-NHC=Se)$_3$] (**2**) in THF solution of 0.1 M [n-Bu_4N]PF_6 with RE: Ag, WE: GC, and CE: Pt.

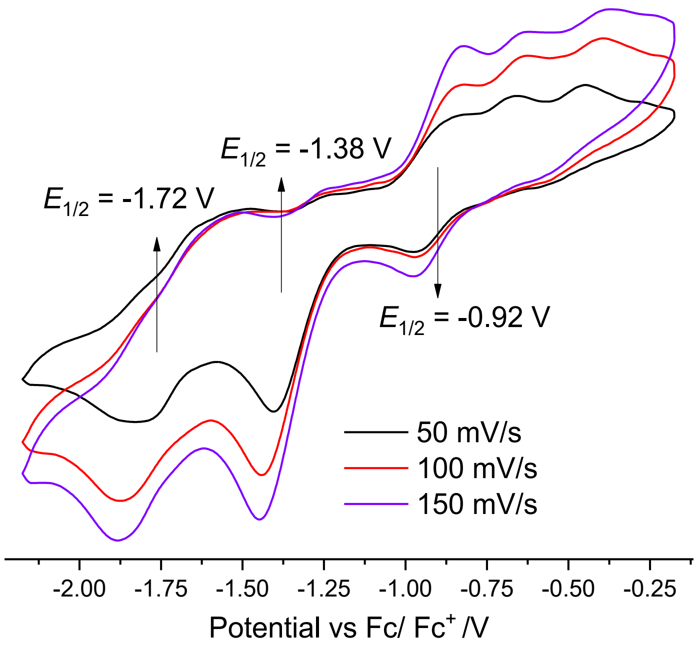

Figure S15. Cyclic voltammogram of complex [Fe(III)(SS-NHC=Se)$_3$] (**2**) in THF solution of 0.1 M [n-Bu$_4$N]PF$_6$ with RE: Ag, WE: GC, and CE: Pt.

…………………………………………………………………………………..

9. EPR studies for catalysis reaction:

Previously, Robinson et al have reported that SS-NHC=$S^{\bullet-}$ radical anion produces five hyperfine lines near g ≈ 2.006 due to the coupling of the radical electron with two ^{14}N nuclei of the C_3N_2 ring of the NHC-backbone.[15-17]

The EPR spectrum of complex **1** (catalyst) shows three sets of hyperfine lines, which are slightly merged. We have explained this feature (Jahn-Teller distortion; three different sets of Fe-S bond length; ~2.25 and ~2.30 Å with each SS-NHC=S ligand). We have recollected the x-ray single crystal data at room temperature and found that the bond lengths remain similar values (see **Table S2**). The effect of Jahn-Teller distortion on bond length is expected to be less severe since this Fe(III) ion of **1** is low-spin [e_g^5 t_{2g}^0]. The EPR spectra of the catalytic reaction solution were recorded at different time intervals (**Figures S16-S25**). It is worth mentioning that complex **1** and four N-donor chelating ligand Me_6TREN were employed in a 1:1 molar ratio. We observed five hyperfine lines at g ≈ 2.006 with very similar coupling constants after 5 min of the reaction. The intensity of which slowly grows over time (Figures S16-25). It suggests that one of the SS-NHC=$S^{\bullet-}$ radical anions has been slowly knocked out from the Fe(III) centre. Another set of hyperfine lines was observed at g ≈ 2.011, which has been assigned to SS-NHC=$S^{\bullet-}$ radical anions, which are still bonded to Fe(III). The g value is higher since it is connected to Fe(III) as expected. The curvature of these EPR lines slightly changes over time, and hence, they were suggested to be for INT-1 and INT-2. Kindly, notice that we have slightly modified our suggested reaction mechanism of the polymerization reaction since free SS-NHC=$S^{\bullet-}$ radical anion was separately observed not to catalyze this polymerization reaction via single electron transfer (SET). Thus, we further proposed that **In**-Br will be coordinated to Fe(III), ion, and then **In**$^{\bullet}$ and Br^- anion will be formed.

The intensity of the EPR signal at g = 2.0112 slowly decreases, while that of the EPR signal at g = 2.0065 increases over 1 hour, which does not change significantly for another hour, accompanied by a change in the color of the solution from purple to green.

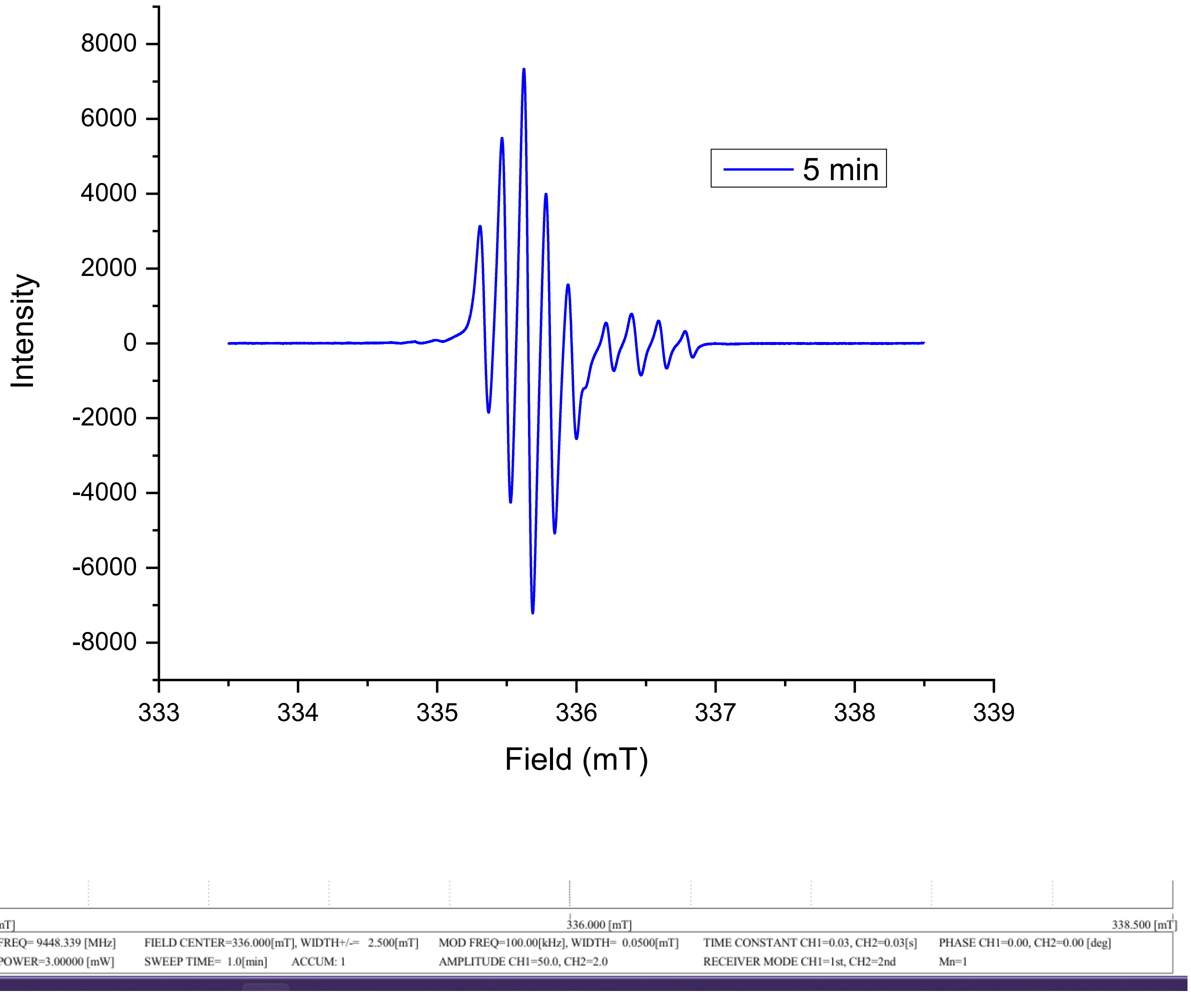

Figure S16. X-band EPR spectrum of the polymerization reaction mixture after 5 minutes.

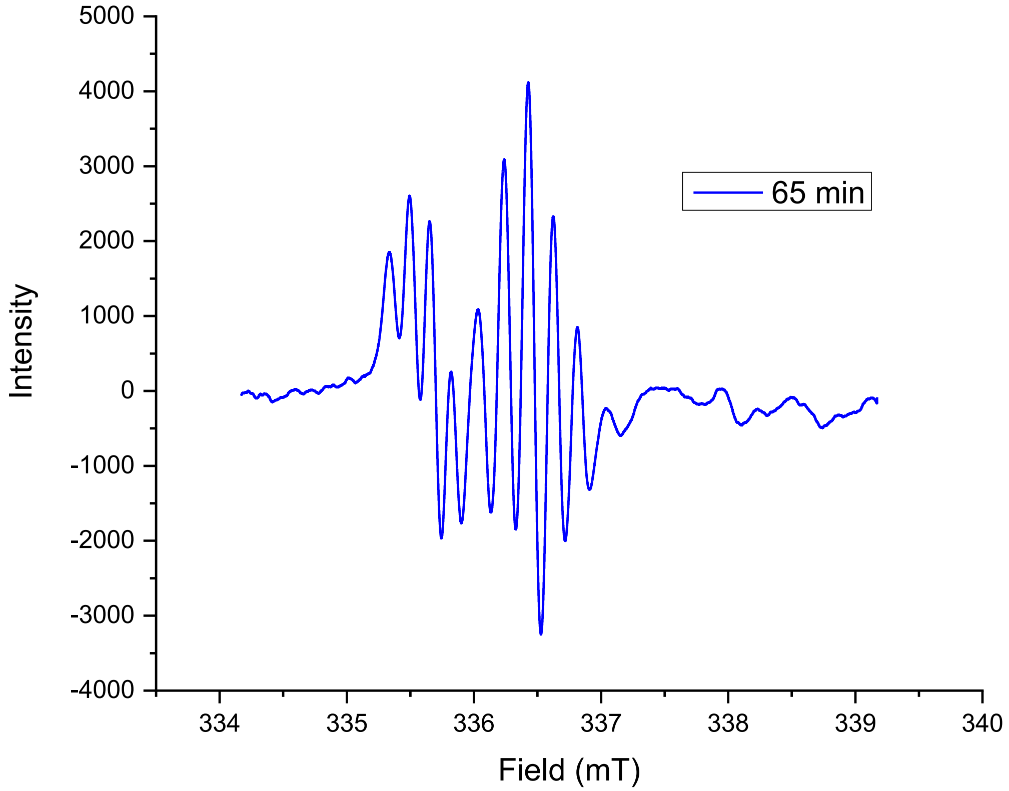

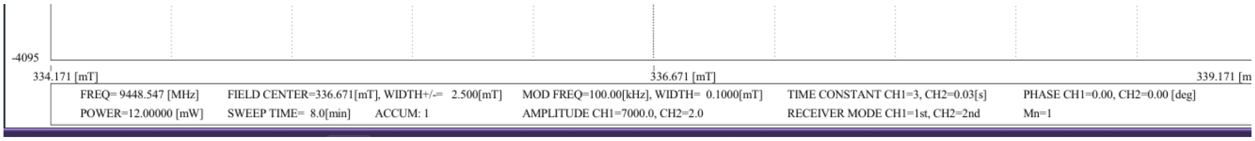

Figure S17. X-band EPR spectrum of the polymerization reaction mixture after 65 minutes.

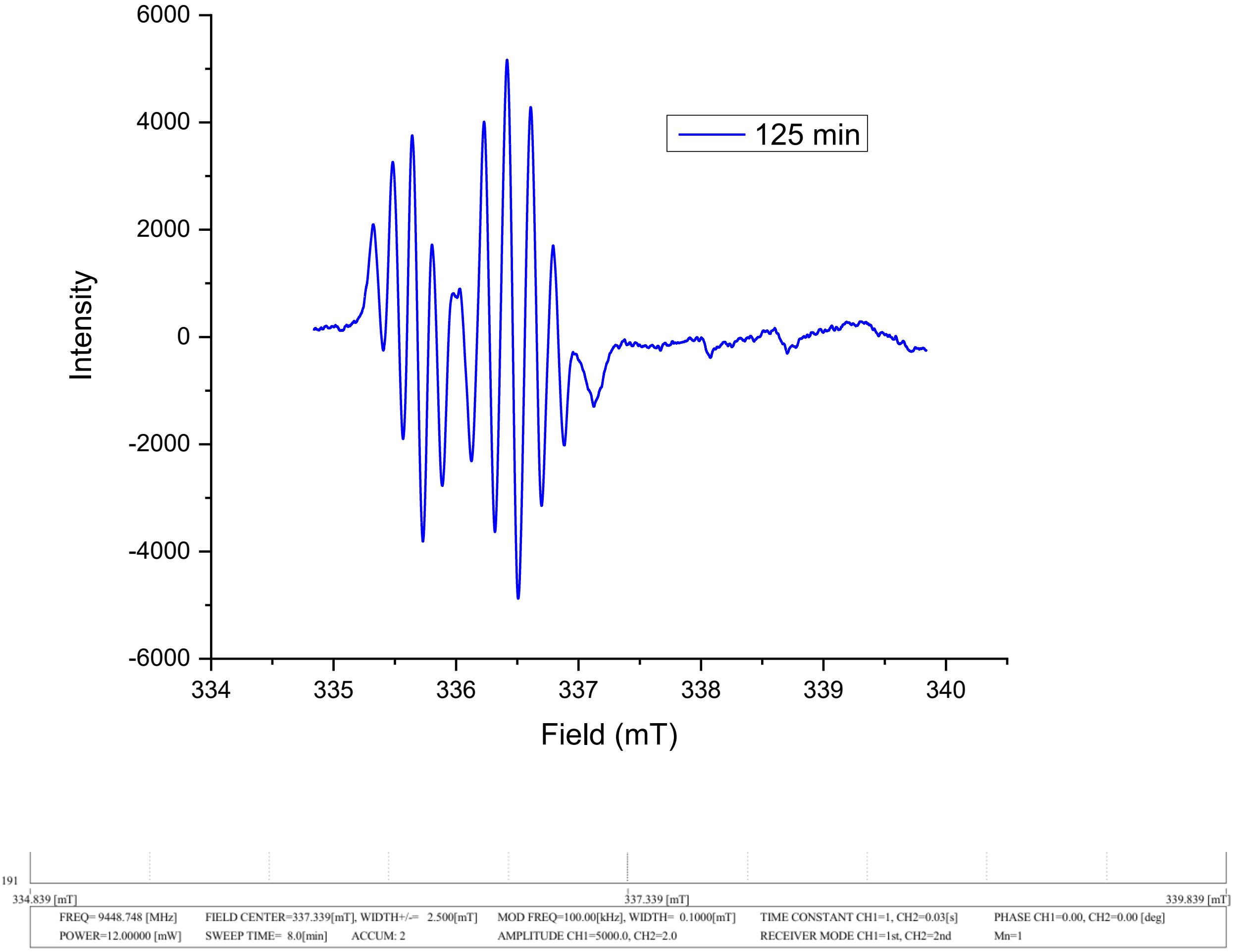

Figure S18. X-band EPR spectrum of the polymerization reaction mixture after 125 minutes.

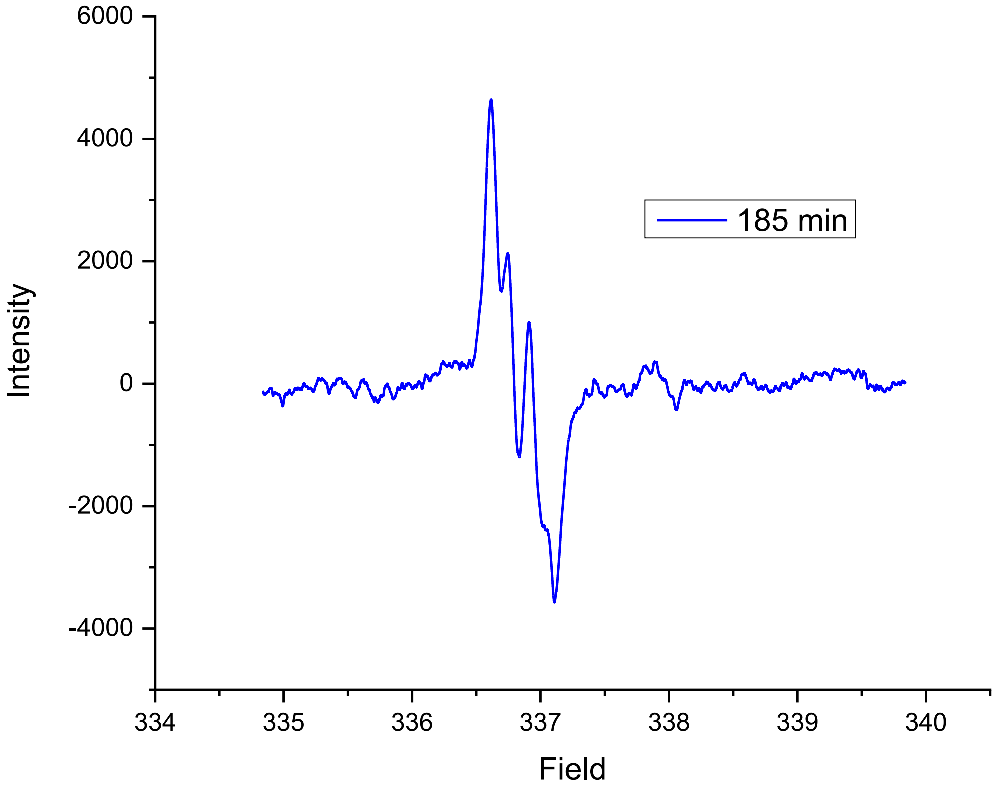

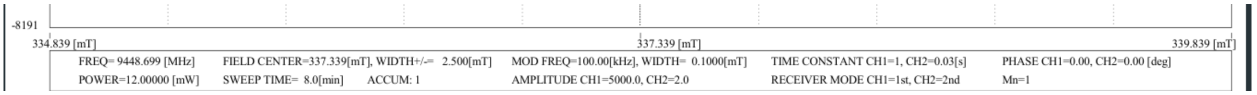

Figure S19. X-band EPR spectrum of the polymerization reaction mixture after 185 minutes.

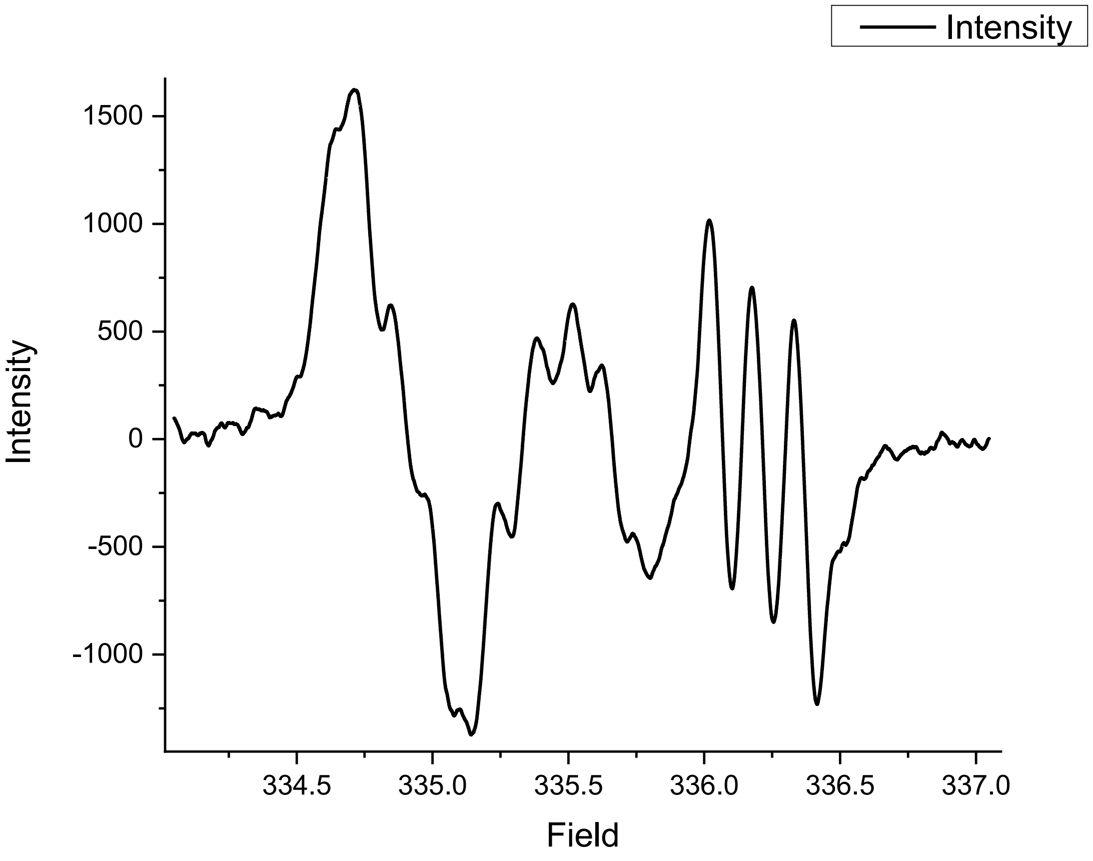

Figure S20. X-band EPR spectrum of catalyst $[Fe(SS\text{-}NHC{=}S)_3]\cdot NHC{=}S$ (**1**·NHC=S).

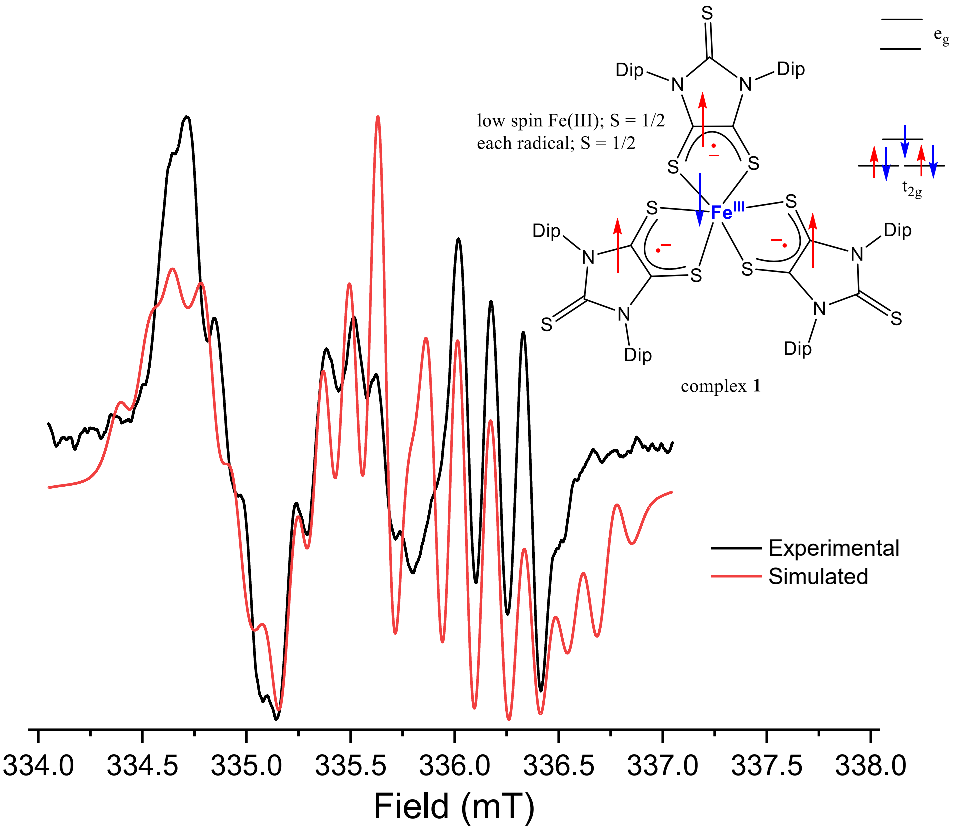

Figure S21. X-band EPR spectrum of catalyst [Fe(SS-NHC=S)$_3$]·NHC=S (**1**·NHC=S). Black and red lines represent experimental and simulated EPR spectra.

X-band EPR spectrum (black) of the unpaired electron in the **octahedral [(SS-NHC=S)Fe(III)(Me$_6$TREN)]$^{2+}$ complex** (left segment - **O$_h$**) after **5 minutes** of the reaction progress. Red and black lines represent the simulated and the experimental spectra of **[(SS-NHC=S)Fe(III)(Me$_6$TREN)]$^{2+}$** complex using the EasySpin program. [**g$_{iso}$(O$_h$)** = 2.01122, LWPP(Gaussian broadening) = 0.0315274 mT, LWPP (Lorentzian broadening) = 0.0129895 mT, **A$_x$(Tbp-N1)** = 4.4889 MHz, **A$_y$(Tbp-N1)** = 4.44428 MHz, **A$_z$(Tbp-N1)** = 4.24699 MHz, **A$_x$(Tbp-N2)** = 4.92802 MHz, **A$_y$(Tbp-N2)** = 4.14565 MHz, **A$_z$(Tbp-N2)** = 4.34421 MHz, X-band experimental frequency = 9.448339 GHz].

```
% Define the spin system for the radical electron (Or Fe3+ LS)
Sys.S = [1/2; 1/2; 1/2]; % 3 types of unpaired electrons
Sys.g = [2.01592; 2.01113; 2.00777]; % Isotropic g-tensor components
Sys.lwpp = [0.0812336 0.0336691]; % Initial guess for Gaussian and Lorentzian linewidth (mT)
```

Sys.Nucs = '14N, 14N'; %
Sys.A = [2.43889 1.38653 2.83904; 3.07108 4.14881 5.10962; 5.34452 4.944 5.19249; 3.98224 4.56721 3.40337; 3.3457 4.08562 5.02318; 3.47036 3.59681 4.7576]; % 2 electrons and 2 nuclei.
Sys.J = [6.84946 4.84894 7.30979];
Sys.dip12 = 9.98743;
Sys.dip13 = 7.1524;
Sys.dip23 = 6.21718;
%Exp.mwFreq = 9.448623; % Microwave frequency (GHz)

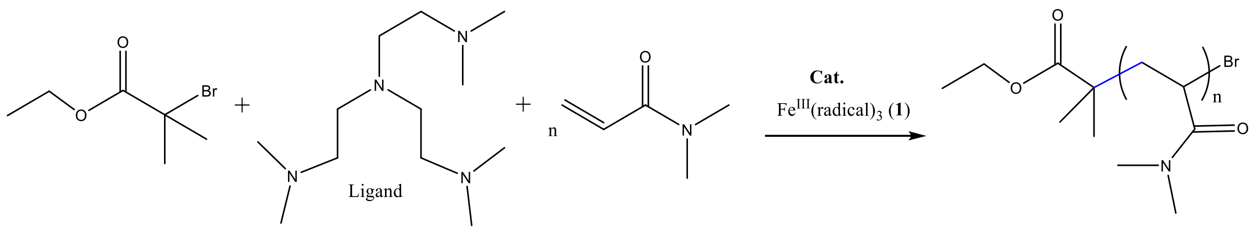

Scheme S4. Catalytic polymerization reaction of complex **1**.

For simplicity, we have split the total EPR spectrum (Figure S16) of polymerization reaction solution into Figure S22 and Figure S23.

5 Min (2.011)

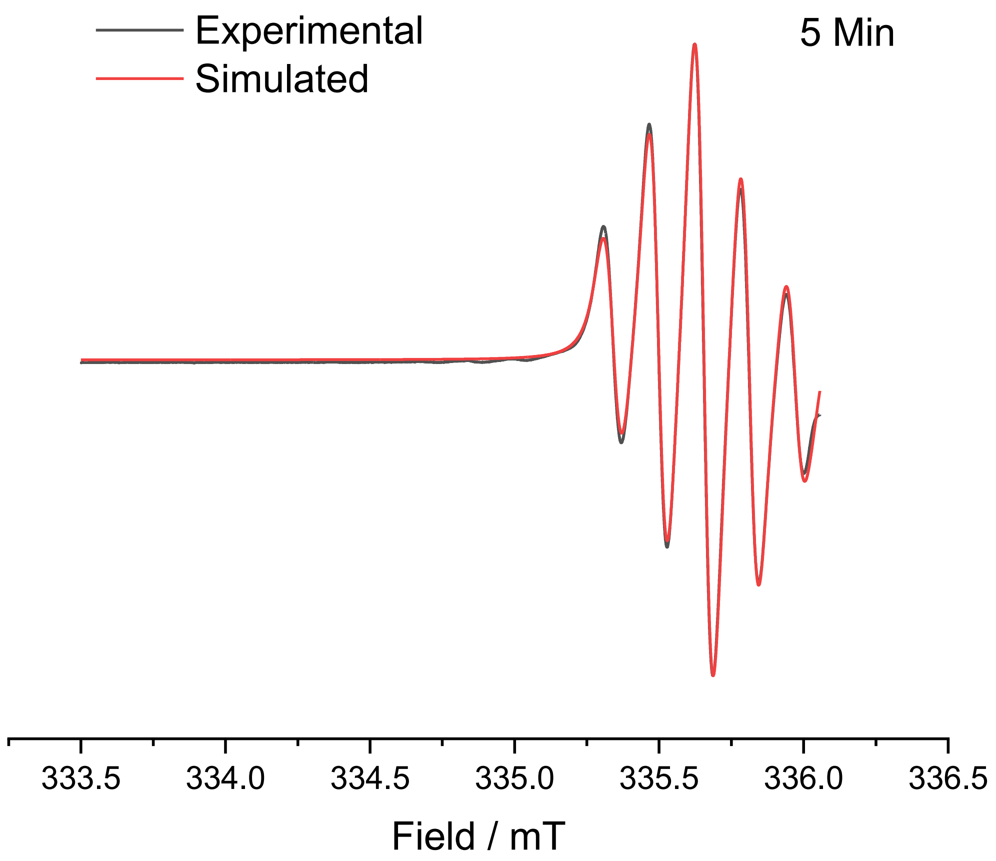

Figure S22. X-band EPR spectrum (black) of the unpaired electron in the **octahedral [(SS-NHC=S)Fe(III)(Me$_6$TREN)]$^{2+}$ complex** (left segment - **Oh**) after **5 minutes** of the reaction progress. Red and black lines represent the simulated and the experimental spectra of **[(SS-NHC=S)Fe(III)(Me$_6$TREN)]$^{2+}$** complex using the EasySpin program. [**g$_{iso}$(Oh)** = 2.01122, LWPP(Gaussian broadening) = 0.0315274 mT, LWPP (Lorentzian broadening) = 0.0129895 mT, **A$_x$(Tbp-N1)** = 4.4889 MHz, **A$_y$(Tbp-N1)** = 4.44428 MHz, **A$_z$(Tbp-N1)** = 4.24699 MHz, **A$_x$(Tbp-N2)** = 4.92802 MHz, **A$_y$(Tbp-N2)** = 4.14565 MHz, **A$_z$(Tbp-N2)** = 4.34421 MHz, X-band experimental frequency = 9.448339 GHz].

5 Min (2.006)

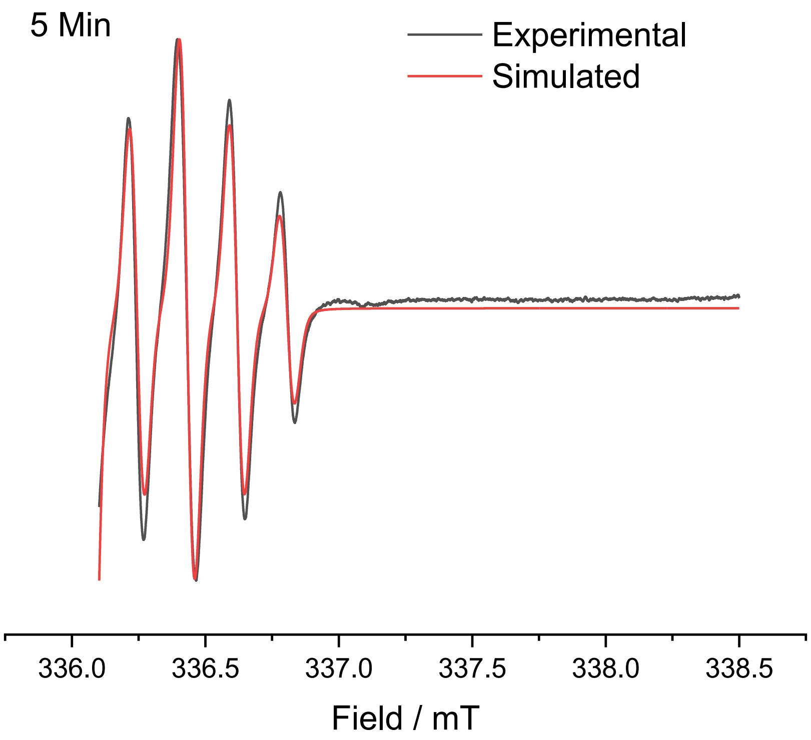

Figure S23. X-band EPR spectrum (black) of the unpaired electron in the liberated **(SS-NHC=S) radical ligand** (right segment - **Rad**) after **5 minutes** of the reaction progress. Red and black lines represent the simulated and the experimental spectra of **the** liberated **(SS-NHC=S) radical ligand** using the EasySpin program. [$\mathbf{g_{iso}}$**(Rad)** = 2.00658, LWPP(Gaussian broadening) = 0.0489864 mT, LWPP (Lorentzian broadening) = 0.0127196 mT, $\mathbf{A_x}$**(Rad-N1)** = 5.26465 MHz, $\mathbf{A_y}$**(Rad-N1)** = 5.12467 MHz, $\mathbf{A_z}$**(Rad-N1)** = 5.11123 MHz, $\mathbf{A_x}$**(Rad-N2)** = 5.23597 MHz, $\mathbf{A_y}$**(Rad-N2)** = 5.36305 MHz, $\mathbf{A_z}$**(Rad-N2)** = 5.44154 MHz, X-band experimental frequency = 9.448339 GHz].

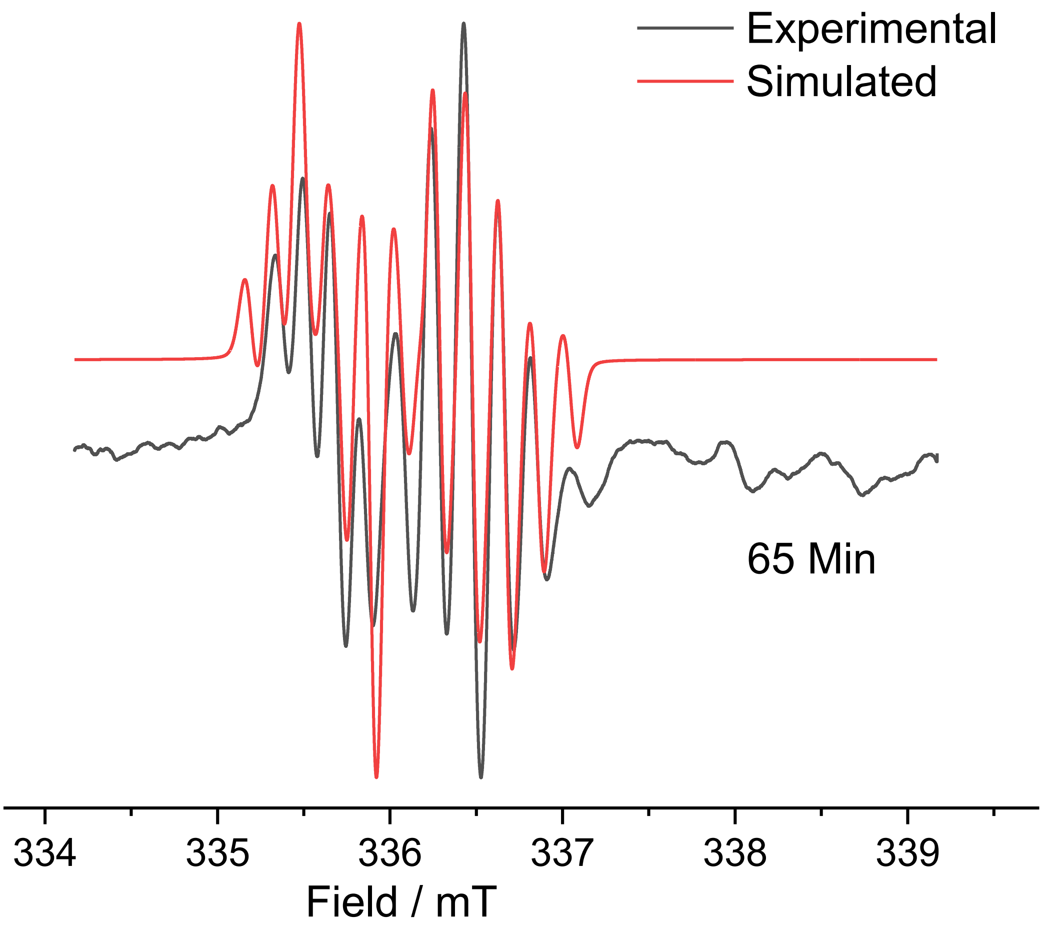

Figure S24. X-band EPR spectrum (black) of the unpaired electron in the **Trigonal bipyramidal** **$[(SS\text{-}NHC{=}S)Fe(III)(Me_6TREN)]^{2+}$ complex** (left segment - **Tbp**) and that of the unpaired electron in the liberated **(SS-NHC=S) radical ligand** (right segment - **Rad**) after **65 minutes** of the reaction progress. Red and black lines represent the simulated and the experimental spectra of **$[(SS\text{-}NHC{=}S)Fe(III)(Me_6TREN)]^{2+}$** complex and **(SS-NHC=S)** radical ligand using the EasySpin program. [$\mathbf{g_{iso}}$**(Rad)** = 2.00638, $\mathbf{g_{iso}}$**(Tbp)** = 2.01095, LWPP(Gaussian broadening) = 0.0723338 mT, LWPP (Lorentzian broadening) = 0.0129895 mT, $\mathbf{A_{x1}}$**(Rad-N1)** = 5.54239 MHz, $\mathbf{A_{y1}}$**(Rad-N1)** = 5.24976 MHz, $\mathbf{A_{z1}}$**(Rad-N1)** = 4.25342 MHz, $\mathbf{A_{x2}}$**(Tbp-N1)** = 4.32174 MHz, $\mathbf{A_{y2}}$**(Tbp-N1)** = 4.47809 MHz, $\mathbf{A_{z2}}$**(Tbp-N1)** = 3.1201 MHz, $\mathbf{A_{x1}}$**(Rad-N2)** = 5.22617 MHz, $\mathbf{A_{y1}}$**(Rad-N2)** = 5.69269 MHz, $\mathbf{A_{z1}}$**(Rad-N2)** = 5.26393 MHz, $\mathbf{A_{x2}}$**(Tbp-N2)** = 4.22454 MHz, $\mathbf{A_{y2}}$**(Tbp-N2)** = 4.36039 MHz, $\mathbf{A_{z2}}$**(Tbp-N2)** = 2.48353 MHz, Exchange coupling **J** = 6.50013 MHz, Dipolar coupling **dip** = 4.96413 MHz, X-band experimental frequency = 9.448547 GHz].

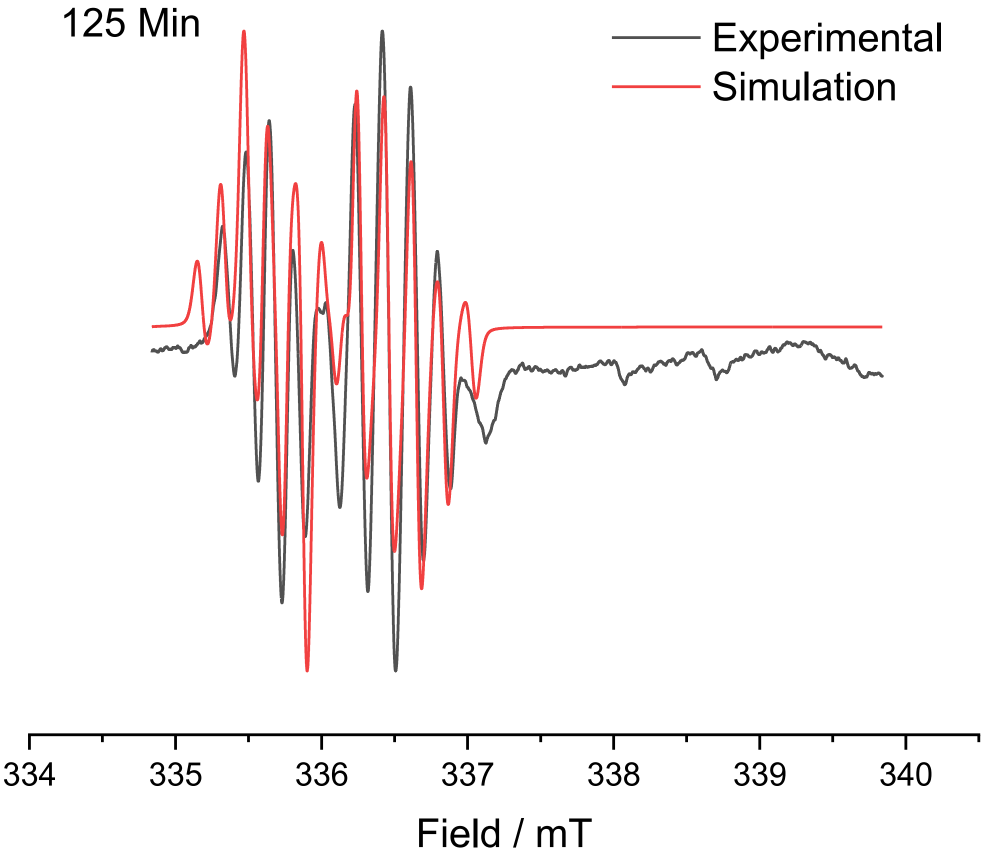

Figure S25. X-band EPR spectrum (black) of the unpaired electron in the **Trigonal bipyramidal [(SS-NHC=S)Fe(III)(Me_6TREN)]$^{2+}$ complex** (left segment - **Tbp**) and that of the unpaired electron in the liberated **(SS-NHC=S) radical ligand** (right segment - **Rad**) after **125 minutes** of the reaction progress. Red and black lines represent the simulated and the experimental spectra of **[(SS-NHC=S)Fe(III)(Me_6TREN)]$^{2+}$** complex and **(SS-NHC=S)** radical ligand using the EasySpin program. [**g_{iso}(Rad)** = 2.00651, **g_{iso}(Tbp)** = 2.01107, LWPP(Gaussian broadening) = 0.056598 mT, LWPP (Lorentzian broadening) = 0.0153911 mT, **A_{x1}(Rad-N1)** = 5.6145, **A_{y1}(Rad-N1)** = 5.10215, **A_{z1}(Rad-N1)** = 4.14654, **A_{x2}(Tbp-N1)** = 4.38373, **A_{y2}(Tbp-N2)** = 4.50208, **A_{z2}(Tbp-N1)** = 3.18864 MHz, **A_{x1}(Rad-N2)** = 5.29131, **A_{y1}(Rad-N2)** = 5.57582, **A_{z1}(Rad-N2)** = 5.04584, **A_{x2}(Tbp-N2)** = 4.33546, **A_{y2}(Tbp-N2)** = 4.48263, **A_{z2}(Tbp-N2)** = 2.56135 MHz,

Exchange coupling **J** = 6.43233 MHz, Dipolar coupling **dip** = 4.72734 MHz, X-band experimental frequency = 9.448748 GHz].

………………………………………………………………………………………………………

10. Computational Methods

Using Density Functional Theory (DFT), the Fe(III)-triradical complex [Fe(III)(SS-NHC=$E^{\bullet -}$)$_3$] (E = S(**1**) and E = Se (**2**)) in triplet spin ground state is optimized with the hybrid B3LYP [S1-3] functional, including a dispersion correction term (D3(BJ))[S4-5]. The Ahlrichs triple-ζ-quality basis set, def2-TZVP[S6], is employed for accuracy, with the Gaussian16[S7] software package facilitating calculations in the gaseous phase. The optimization confirms minima on the potential energy surface, with no negative frequencies. For simplification, the Dipp group (in **1** and **2**) is replaced by a methyl group (**1′ and 2′**). The NBO 6.0 program[S8] is used for Natural Bond Orbital (NBO)[S9] analysis and calculating spin densities at the B3LYP-D3(BJ)/Def2-TZVP level. Energy decomposition analysis (EDA)[S10] is performed alongside natural orbital for chemical valence (NOCV)[S11-S12] analysis to investigate electron bonding behaviour in this Fe-complex analogue using the ADF 2020 software[S13]. This method provides a detailed understanding of bond formation between fragments, offering flexibility in exploring different charge and electron configurations. The previously optimized geometries are used for the EDA-NOCV[S14-15] calculations at the B3LYP-D3(BJ)/Def2-TZP level of theory. ADF employs the Morokuma-type energy decomposition method, splitting the total energy into two components: ΔE_{prep} (the energy required to deform the fragments into the desired geometry) and ΔE_{int} (the interaction energy between the fragments forming the complex). ΔE_{int} is further split into four physical components in the ADF output file as,

$$\Delta E_{\mathrm{int}} = \Delta E_{\mathrm{elstat}} + \Delta E_{\mathrm{Pauli}} + \Delta E_{\mathrm{orb}} + \Delta E_{\mathrm{disp}}$$

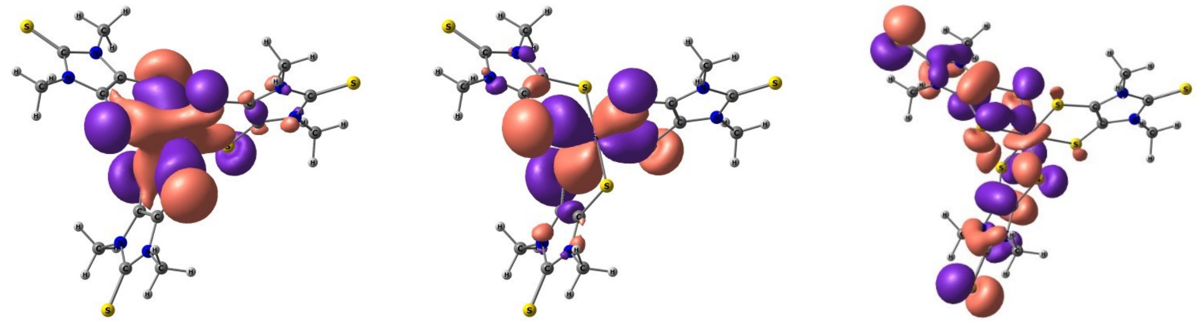

α-SOMO-18 (ε = -8.60 eV) α-SOMO-17 (ε = -8.51 eV) α-SOMO-2 (ε = -5.47 eV)

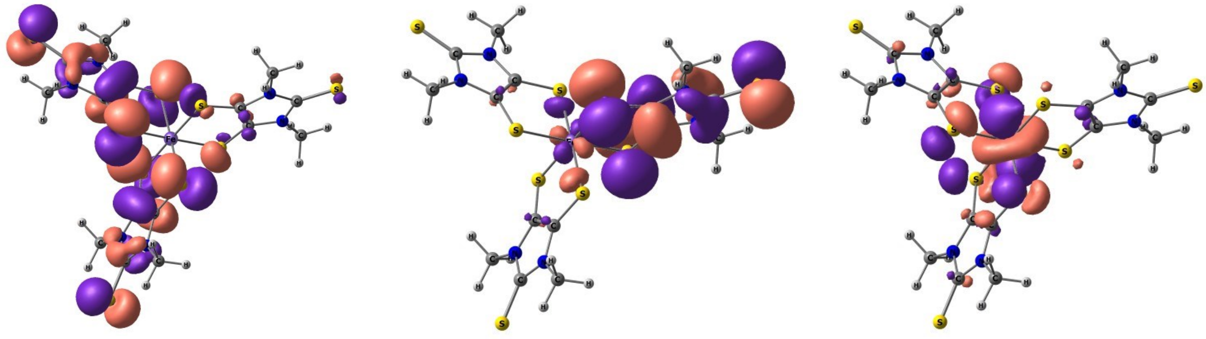

α-SOMO-1 (ε = -5.25 eV) α-SOMO (ε = -5.11 eV) α-LUMO (ε = -3.03 eV)

Figure S26. The molecular orbitals of Fe(III)-triradical complex [Fe(III)(SS-NHC=$S^{\bullet-}$)$_3$] (**1′**) as calculated at B3LYP-D3(BJ)/def2TZVP level of theory.

α-SOMO-18 and α-SOMO-17 represent the filled bonding orbitals between the Ligand and Fe atom, while α-SOMO-2, α-SOMO-1 and α-SOMO represent the singly occupied radicals over the ligand system. The energy gap between α-SOMO and β-LUMO is -1.42 eV.

Table S7. The EDA-NOCV results for Fe(III)-triradical complex [Fe(III)(SS-NHC=$S^{\bullet-}$)$_3$] (**1′**) at the B3LYP-D3(BJ)/def2TZP level of theory of L_2Fe–L bond as L_2Fe^{+1} (D) and L^{-1} (D) fragment for different interacting dative (D) possibility. Energies are in kcal/mol. L = SS-NHC=$S^{\bullet-}$.

Complex	Bond Type	Fragments	ΔE_{int}	ΔE_{pauli}	ΔE_{elec}[a]	ΔE_{dis}[a]	ΔE_{orb}[a]

[Fe(III)(SS-NHC=$S^{\bullet-}$)$_3$] (**1**)	D	FeL_2^{+1} (Q) + L^{-1} (D)	-160.7	210.5	-217.8	-14.5	-138.9
	D′	FeL_2^{+1} (Q) + L^{-1} (D)	-166.1	210.3	-217.8	-14.5	-144.1
	D″	FeL_2^{+1} (D) + L^{-1} (D)	-160.8	173.3	-198.6	-14.5	-121.1

Table S8. The EDA-NOCV results for [Fe(III)(SS-NHC=$S^{\bullet-}$)$_3$] (**1′**) at the B3LYP-D3(BJ)/def2TZP level of theory of $L_2Fe{-}L$ bond as L_2Fe^+ (D) and L^- (D) fragment electronic doublet state as interacting fragments. Energies are in kcal/mol. [a]The values in the parentheses show the contribution to the total attractive interaction ΔE_{elec} + ΔE_{orb} +ΔE_{disp}[a], and [b] value in the parentheses shows the contribution to the total orbital interaction. L = SS-NHC=$S^{\bullet-}$.

Energy	Interaction	L_2Fe^+ (D) + L^- (D) Overall (T)
ΔE_{int}		-160.8
ΔE_{Pauli}		173.3
ΔE_{disp}[a]		-14.5 (4.3%)
ΔE_{elstat}[a]		-198.6 (59.4%)
ΔE_{orb}[a]		-121.1 (36.2%)
$\Delta E_{orb(1)}$[b]	L_2Fe^+ (D) ← L^- (D) π e^- donation	-43.2 (35.6%)
$\Delta E_{orb(2)}$[b]	L_2Fe^+ (D) ← L^- (D) σ e^- donation	-27.6 (22.8%)
$\Delta E_{orb(3)}$[b]	L_2Fe^+ (D) ← L^- (D)	-26.1 (21.6%)
$\Delta E_{orb(rest)}$[b]		-24.2 (20.0%)

$[Fe(III)(SS\text{-}NHC{=}S^{\bullet-})_3]$ (**1′**)	L_2Fe^{+} (D)	$SS\text{-}NHC{=}S^{\bullet-}$ (D)
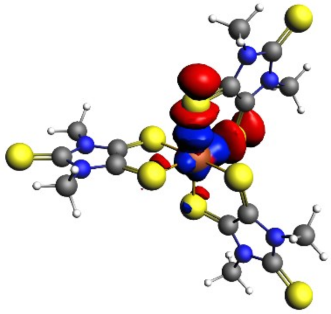 $\Delta\rho_{(1)}$ $\Delta E_{orb(1)}$ = -43.2; $\lvert\nu_1\rvert$ = 0.86	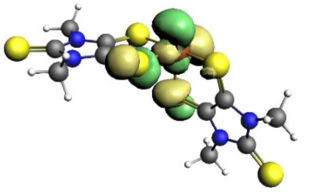 LUMO+1 (ε = -6.01 eV)	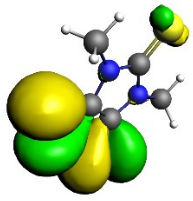 HOMO (ε = -1.52 eV)
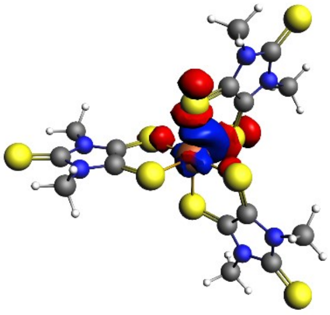 $\Delta\rho_{(2)}$ $\Delta E_{orb(2)}$ = -27.6; $\lvert\nu_2\rvert$ = 0.61	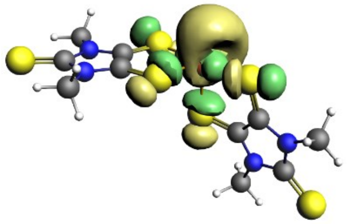 LUMO+2 (ε = -5.62 eV)	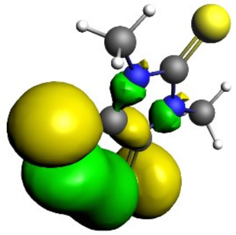 HOMO-1 (ε = -2.26 eV)
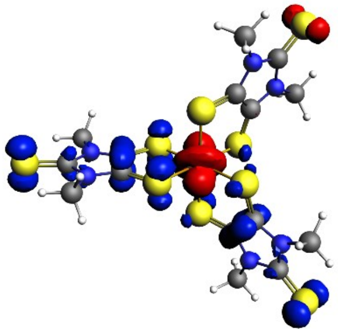 $\Delta\rho_{(3)}$ $\Delta E_{orb(3)}$ = -26.1; $\lvert\nu_3\rvert$ = 0.96/0.19	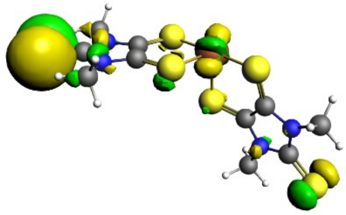 HOMO (ε = -9.74 eV) 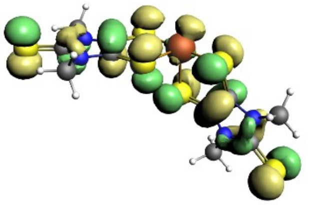 LUMO (ε = -7.51 eV)	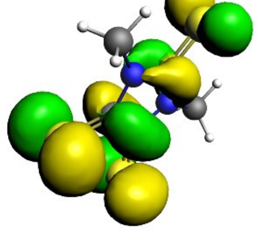 SOMO (ε = +0.092 eV)

Figure S27. The shape of the deformation densities $\Delta\rho_{(1)-(3)}$ that correspond to $\Delta E_{orb(1)-(3)}$, and the associated molecular orbitals of Fe-complex (**1′**) for L_2Fe–L bond as L_2Fe^+ (D) and L^1 (D) fragment in electronic doublet state as interacting fragments. at the B3LYP-D3(BJ)/def2TZP level of theory. Energies are in kcal/mol. The isosurface value is 0.002 au for $\Delta\rho_{(1-3)}$. The eigenvalues $|\nu_n|$ give the size of the charge migration in e. The charge flow direction of the deformation densities is from red→blue. D = doublet state. L = SS-NHC=$S^{\bullet-}$.

Table S9. The Optimized Coordinates of **1′** at B3LYP-D3(BJ)/def2TZVP level of theory.

1′ (Triplet State)

E = -5759.349936 hf

Fe	-0.177166000	0.000175000	0.000043000
S	-7.014076000	0.001434000	-0.000021000
S	-1.823339000	0.711028000	-1.520248000
S	1.444516000	-0.765035000	1.471057000
S	-1.823604000	-0.709638000	1.520422000
S	-0.160712000	2.001438000	1.182892000
S	-0.162016000	-2.001003000	-1.182876000
S	1.444861000	0.764417000	-1.471093000
S	3.913089000	-5.412841000	0.040576000
S	3.916648000	5.410539000	-0.040679000
N	-4.518058000	-0.467057000	0.993794000
N	-4.517882000	0.469133000	-0.993773000
N	2.881332000	-3.060500000	0.952657000
N	1.825558000	-3.872918000	-0.796972000
N	1.828110000	3.872020000	0.796940000
N	2.883278000	3.058892000	-0.952726000
C	-5.360708000	0.001176000	0.000001000
C	-3.204301000	0.299782000	-0.635463000
C	-3.204416000	-0.298086000	0.635536000
C	1.869085000	-2.183953000	0.659730000

C	2.879430000	-4.124661000	0.065709000
C	1.191874000	-2.704344000	-0.460762000
C	1.193641000	2.703868000	0.460762000
C	2.882129000	4.123054000	-0.065774000
C	1.870461000	2.183022000	-0.659755000
C	1.431030000	-4.727367000	-1.899298000
H	1.390289000	-4.139819000	-2.815928000
H	2.167687000	-5.520125000	-1.988757000
H	0.446334000	-5.153703000	-1.706425000
C	3.814932000	-2.893448000	2.048625000
H	4.550363000	-3.689944000	1.986016000
H	4.299957000	-1.921079000	1.967524000
H	3.285431000	-2.948078000	2.999980000
C	3.816742000	2.891206000	-2.048713000
H	4.552716000	3.687201000	-1.986120000
H	4.301106000	1.918507000	-1.967623000
H	3.287261000	2.946196000	-3.000058000
C	1.434183000	4.726737000	1.899274000
H	1.393411000	4.139308000	2.815979000
H	2.171192000	5.519198000	1.988490000
H	0.449618000	5.153450000	1.706573000
C	-4.953437000	1.058007000	-2.244187000
H	-4.571030000	2.075466000	-2.324770000
H	-4.574994000	0.469400000	-3.079833000
H	-6.039370000	1.063505000	-2.250029000
C	-4.953836000	-1.055796000	2.244194000
H	-6.039768000	-1.061919000	2.249551000
H	-4.570838000	-2.072992000	2.325229000

H -4.576146000 -0.466729000 3.079863000

……………………………………………………………

11. A. Experimental procedures for polymerization reaction:

A.1 Materials.

All materials were purchased from Sigma Aldrich or Fischer Scientific and used as received unless otherwise stated. The monomers, N, N-Dimethylacrylamide (DMA, 98%, Sigma-Aldrich), N-isopropylacrylamide (NIPAM, 98%, Sigma-Aldrich), 2-(Dimethylamino)ethyl Methacrylate (DMAEMA, 98%, Sigma-Aldrich), Benzyl Methacrylate (BzMA, 98%, Sigma-Aldrich), and Methyl methacrylate (MMA, 98%, Sigma-Aldrich), were freed from inhibitors by passing them through a column of basic alumina. and ethyl α-bromoisobutyrate (EBiB, 98%, Aldrich). Tris [2-(dimethylamino)ethyl]-amine (Me_6TREN, >98%, TCI Chemicals). Methanol (Merck, India), Tetrahydrofuran (THF, 99%, Merck), Chloroform ($CHCl_3$, Merck, India), Diethyl ether (thermofischer), n-hexane (thermofischer) and acetone were used as received for all the experiments. Deuterated chloroform ($CDCl_3$, purity > 99.8%) used for NMR spectroscopy was purchased from Aldrich.

A.2 Homopolymerization of Different class of monomers

The procedure of complex **1** mediated OMRP of DMA. Complex **1**-mediated RDRP of DMA (P2, Table S5), a glass vial (5 mL) charged with complex **1** (2 mg, 0.123 mmol), EBiB (18 μL, 0.123 mmol), Me_6TREN (33 μL, 0.123 mmol), Anhydrous THF (200 μL, 3.99 mmol) and degassed DMA (200 μL, 12.31 mmol) is sealed with a silicone rubber septum inside glove box. The reaction mixture was then stirred for 8 hrs. At the end of the reaction, the crude product was dissolved in THF, the excess solvent was evaporated by rotary evaporator and polymer was isolated by precipitation into prechilled diethyl ether (×3 S4 times). Finally the product was dried under

vacuum (10^{-3} bar, 60 °C) for 12 h and characterized by NMR spectroscopy and SEC. ^{1}H NMR (400 MHz, $CDCl_3$, δ ppm of P2, Table S5, Figure S7(A)) at 1.5 ppm (–$CH_2C(C\underline{H}_3)$ of DMA), 1.5–2.1 ppm (CCH_2 connecting DMA chains), 2.1–2.5 ppm (CH of DMA chains), 2.6 (–$N(C\underline{H}_3)_2$ of DMA)[1]. Following the similar procedure, we performed the homopolymerization of the other monomers including N,N-dimethyl isopropoyl acrylamide (NIPAM), N,N-dimethyl aminoethyl methacrylate (DMAEMA) and Benzyl methacrylate (BzMA). A similar procedure was carried out with complex **2**.

A.3 Kinetics Studies of OMRP of Homopolymers by Complex 1

A series of experiments were carried out for the OMRP of DMA mediated by Complex **1** and they were stopped at different polymerization times (0.5 h to 8 h) to monitor (i) the conversion of DMA by gravimetry and (ii) the evolution of the molar masses with time by means of SEC in DMF. The data is summarized in Table S5.

A.8 Syntheses of Various polymer with varying $[M]_0/[EBiB]_0$ via OMRP mediated by Complex 1

Synthesis of polymer by changing the $[DMA]_0/[EBiB]_0$ feed ratio while keeping the other reaction parameters constant, PDMA of various molar masses (DP = 25-150) were synthesized. The M_n data is summarized in Table S11.

A.9 Syntheses of Diblock Copolymers.

OMRP of homopolymers by complex **1** at ambient temperature was set up as mentioned above. After the completion of reaction, the obtained homopolymers were dissolved in the THF. The

homopolymers were used as a macroinitiator and catalyst, and ligands and other monomers were added to the reaction mixture inside the glove box. In this case, $PDMA_{50}$-Br is used as the macroinitiator, and MMA is used as the second monomer for chain extension. The reaction mixture was set for another 24 hours at ambient conditions. After which, the reaction mixture was quenched by exposing to air, and then the crude was precipitated in diethyl ether. The obtained product was centrifuged and dried for 6 hrs in vacuum oven. The composition of the block copolymer was determined by the functional group were identified through ATR IR spectra (Figure S32) and ^{1}H NMR spectroscopies in $CDCl_3$ (Figure S33).

B. Characterization.

Nuclear Magnetic Resonance (NMR) Spectroscopy. ^{1}H spectroscopy was used to identify the compositions and structures. ^{1}H NMR spectra were recorded at 25 °C on a Bruker 400 MHz s using $CDCl_3$ as a solvent.

IR Spectra. IR Spectra were taken on a Perkin Elmer Spectrum 100 instrument in ATR mode.

Size Exclusion Chromatography (SEC) Measurements. Molar masses (M_{ns}) and dispersities ($Đ_s$) of the synthesized polymers were assessed with triple-detection GPC (from Agilent Technologies), DMF (containing 0.1 wt % of LiCl) was used as the eluent and narrow linear poly(methyl methacrylate) standards were used to calibrate the instrument.

Glove Box. The polymerization reactions were set up using the LABPRO 1800 series of glove box under nitrogen condition.

UV Vis spectra. The UV-vis spectra of polymer were measured on a Shimadzu UV-2600 spectrophotometer.

Table S10. Control experiments for the OMRP of DMA

Entry	Reaction condition[a]	Time (h)	Conv.[b] (%)	$M_{n,theo.}$[c] (g mol^{-1})	$M_{n,SEC}$[d] (g mol^{-1})	Đ[d]
P1	DMA/EBiB/complex **1**/bpy	24	23	1300	1900	1.91
P2	DMA/EBiB/complex **1**/PMDETA	24	31	1700	2400	1.63
P3	DMA/EBiB/complex **1**/Me_6TREN	24	100	5200	5300	1.15
P4	DMA/complex **1**/Me_6TREN	24	<1	-	-	-
P5	DMA/EBiB/complex **1**	24	<1	-	-	-
P6	DMA/EBiB/Me_6TREN	24	<1	-	-	-
P7	DMA/EBiB/Me_6TREN/[$(THF)_2$Li(SS-NHC=S)]	24	<1	-	-	-

[a]Reaction Conditions: $[DMA]_0/[EBiB]_0/[complex\ \mathbf{1}]_0/[Ligand]_0$ = 50/1/1/1. reaction temperature = 25 °C. [b]Determined gravimetrically based on monomer feed. [c]Calculated using yield as conversion and the following equation: $M_{n,theo}$ = ($[DMA]_0/[EBiB]_0$ × monomer conversion × DMA) + MEBiB, where DMA (= 99.13 g mol^{-1}) and MEBiB (= 195.05 g mol^{-1}) are the molecular weight of DMA and EBiB, respectively. [d]Obtained from SEC measurements, narrow linear poly(methyl methacrylate) standards were used to calibrate the SEC instrument.

Table S11. Reaction conditions and results for the OMRP of DMA catalyzed by complex **1**

Entry	Initiator [I]	Monomer [M]	$[M_o]/[I_o]$	Time (h)	Conv.[b] (%)	$M_{n,theo.}$[c] (g mol^{-1})	$M_{n,SEC}$[d] (g mol^{-1})	*Đ*[d]
P1	EBiB	DMA	25	8	-	2700	2900	1.14
P2	EBiB	DMA	50	8		5200	5300	1.15
P3	EBiB	DMA	75	8		7600	7850	1.15
P4	EBiB	DMA	100	8		10100	10000	1.15
P5	EBiB	DMA	150	8		15050	14900	1.17

[a]Reaction Conditions: $[DMA]_0/[EBiB]_0/[complex\ \mathbf{1}]_0/[Ligand]_0$ = 50/1/1/1. reaction temperature = 25 °C. [b]Determined gravimetrically based on monomer feed. [c]Calculated using yield as conversion and the following equation: $M_{n,theo}$ = ($[DMA]_0/[EBiB]_0$ × monomer conversion × DMA) + MEBiB, where DMA (= 99.13 g mol^{-1}) and MEBiB (= 195.05 g mol^{-1}) are the molecular

weight of DMA and EBiB, respectively. [d]Obtained from SEC measurements, narrow linear poly(methyl methacrylate) standards were used to calibrate the SEC instrument.

Complex **1**·0.25*n*-hexane was dried under vacuum to obtain complex **1** free of lattice ligand or lattice solvent.

Table S12. Reaction condition and results for the OMRP of monomers with complex **(1)**, **(2)** and **(3)**

Entry	Monomer [M]	Complex	Time (h)	Conv. (%)	$M_{n,theo.}$[b] (g.mol^{-1})	$M_{n,SEC}$[c] (g.mol^{-1})	*Đ*[d]
P1	NIPAM	**1·NHC=S**	8	100	5850	5500	1.18
P2	DMAEMA	**1·NHC=S**	8	100	8050	7300	1.19
P3	BzMA	**1·NHC=S**	24	100	9000	9250	1.17
P4	DMA	**2**	24	100	5200	5300	1.16
P5	NIPAM	**2**	24	100	5850	5700	1.19
P6	DMAEMA	**2**	24	100	8050	7600	1.18
P7	BzMA	**2**	24	100	9000	8900	1.16
P8	DMA	**1**	24	100	5200	4900	1.14
P9	NIPAM	**1**	24	100	5850	6000	1.17
P10	DMAEMA	**1**	24	100	8050	7800	1.19
P11	BzMA	**1**	24	100	9000	9100	1.17

[a]Reaction Conditions: $[M]_0/[EBiB]_0/[Complex]_0/[Ligand]_0 = 50/1/1/1$. reaction temperature = 25 °C. [b]Determined gravimetrically based on monomer feed. [c]Calculated using yield as conversion and the following equation: $M_{n,theo} = ([M]_0/[EBiB]_0 \times \text{monomer conversion} \times M) + M_{EBiB}$, where M (NIPAM = 113.16, DMAEMA= 157.21 and BzMA = 176.21 g mol^{-1} respectively) and M_{EBiB} (= 195.05 g mol^{-1}) are the molecular weight. [d]Obtained from SEC measurements, narrow linear poly(methyl methacrylate) standards were used to calibrate the SEC instrument.

Table S13. Reaction condition and results of diblock copolymers.[a]

Entry	initiator	Second Monomer	copolymer	time (h)	$M_{n,SEC.}$[c] (g/mol)	*Đ*[c]

P1	$PDMA_{50}$-Br	MMA	$PDMA_{50}$-*b*-$PMMA_{30}$	24	10600	1.20
P2	$PNIPAM_{50}$-Br	DMA	$PNIPAM_{50}$-*b*-$PDMA_{30}$	24	10200	1.19

[a]First blocks were synthesized by OMRP of M (=DMA or NIPAM) at 25 °C.

[a]Reaction Conditions: $[Initiator]_0/[Complex\ \mathbf{1}]_0/[Me6TREN]_0$ = 1/1/1. Reaction temperature = 25 °C, Polymerization time = 24 h for $PDMA_{50}$-*b*-$PMMA_{30}$ and $PNIPAM_{50}$-*b*-$PDMA_{30}$ respectively. [b]Obtained from theoretical calculations. [c]Obtained from SEC measurements, narrow linear PMMA standards were used to calibrate the SEC instrument.

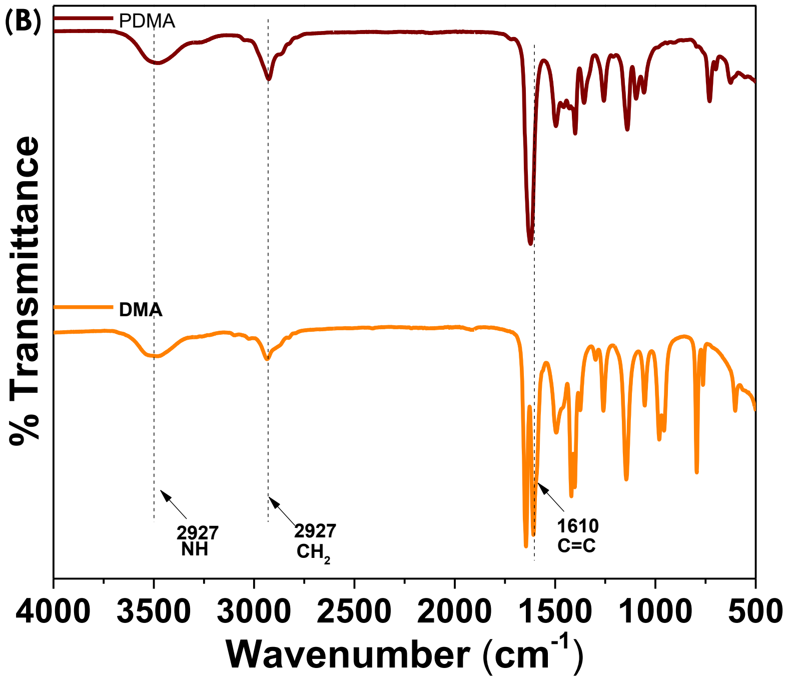

Figure S28. ATR-IR spectra of $PDMA_{50}$-Br (P2, Table S11).

The IR peaks (Figure S30(B)) at 2927, 2927, 1610, 1016 cm^{-1} corresponds to -NH, $-CH_3$, -C-O-NH and -C-N groups respectively.

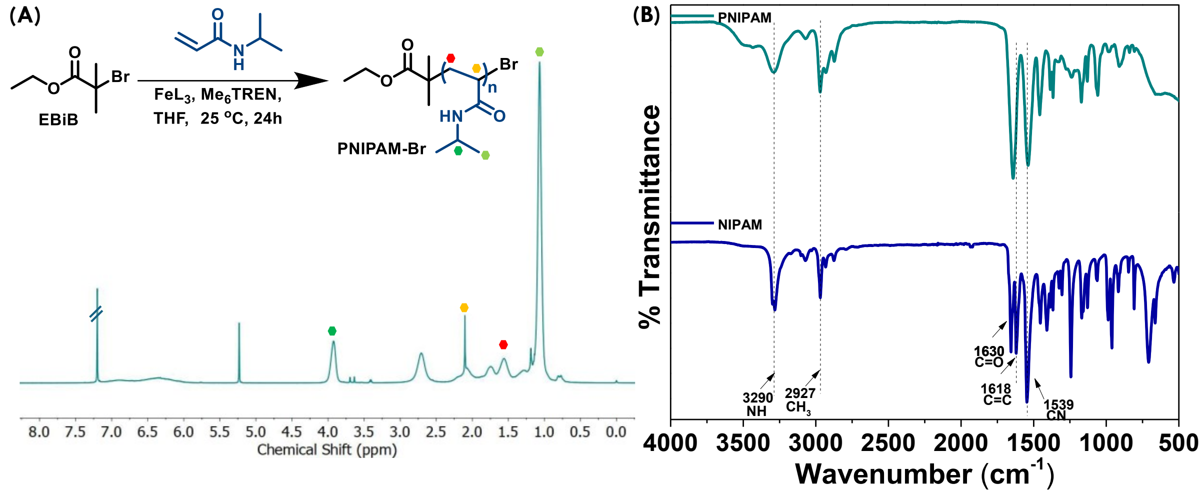

Figure S29. (A) ^{1}H NMR spectra of $PNIPAM_{50}$-Br (in $CDCl_3$). **(B)** ATR-IR spectra of $PNIPAM_{50}$-Br (P2, Table S11).

^{1}H NMR (600 MHz, $CDCl_3$, δ ppm, Table S12, Figure S31(A)): ^{1}H NMR (400 MHz, $CDCl_3$, δ ppm, Table S12, Figure S31(A)): 1.0-1.2 (–CH(C$\underline{H}_3$) of NIPAM repeat; 1.4-2.2 (–CH-C$\underline{H}_2$ of the NIPAM repeat unit); 2.7-3.4 (–CH-CH_2 of the NIPAM repeat unit); 3.6-4.0 (–CH(CH_3) of NIPAM repeat unit adjacent to CONH); 5.3-5.7 (–CONH of NIPAM repeat unit)[S17] [S17b]. IR spectrum of PNIPAM clearly showed the main characteristic peaks of the polymer at 1453, 1536, 1635, 2927 and 3290 cm^{-1} which are attributed to -CH_3 (bend.), C-N (stretch.), C=O (stretch.), CH_3 and NH group.

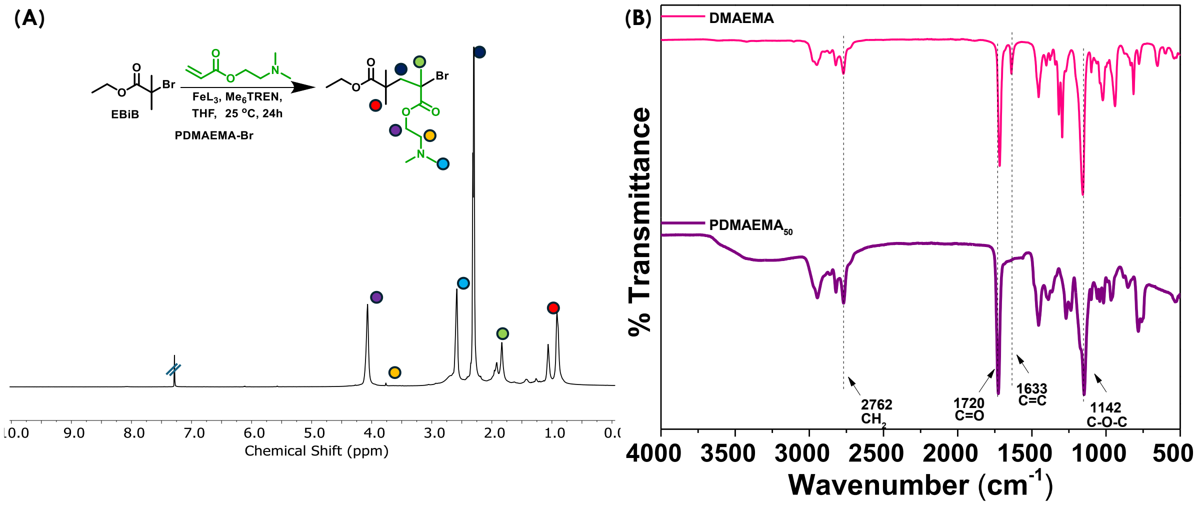

Figure S30. (A) ^{1}H NMR spectra of PDMAEMA$_{50}$-Br (in $CDCl_3$). **(B)** ATR-IR spectra of PDMAEMA$_{50}$-Br (P2, Table S13).

^{1}H NMR depicts signals at 0.7–1.1 ppm (–$CH_2C(C\underline{H}_3)$ of DMAEMA), 1.5–2.1 ppm ($CH_2C(CH_3)$ of DMAEMA), 2.1–2.4 ppm (–$N(C\underline{H}_3)_2$ of DMAEMA), 2.4–2.7 ppm (–OCH_2CH_2– of DMAEMA) and 3.9–4.2 ppm (–$OC\underline{H}_2CH_2$– of DMAEMA [S18]. The IR peaks (Figure S32(B) at 2762, 1720, 1142, 1061 cm^{-1} corresponds to -CH_3, -C=O, -C-O-C and -C-N groups respectively.

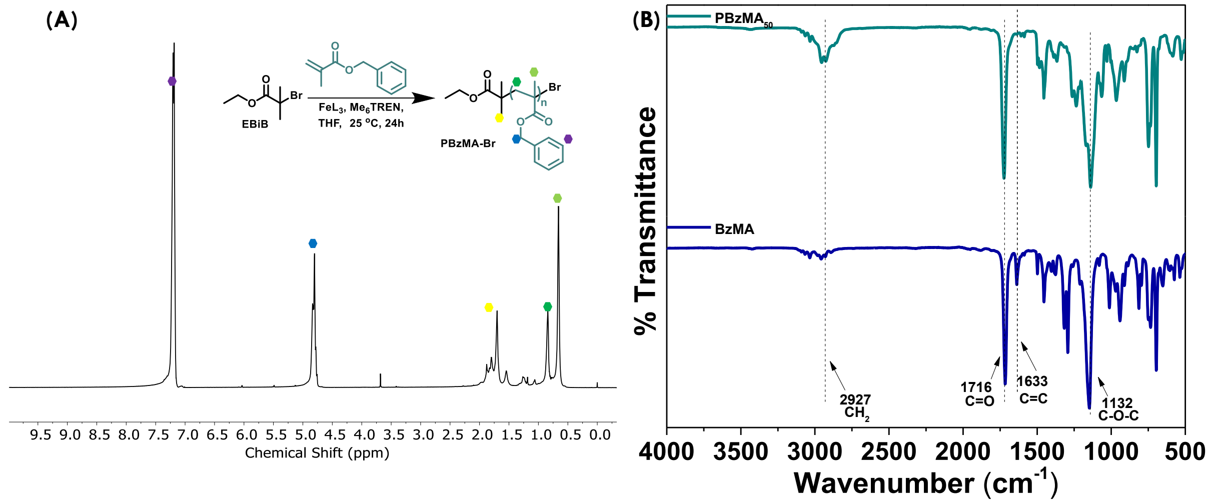

Figure S31. (A) ^{1}H NMR spectra of PBzMA$_{50}$-Br (in $CDCl_3$). **(B)** ATR-IR spectra of PBzMA$_{50}$-Br (P3, Table S12).

^{1}H NMR spectra of PBzMA$_{50}$-Br exhibited the typical signals of PBzMA at 7.336-7.246 (H–Ar), 5.090-4.971 (H_2C–Ar), 1.927 (CH_3), 0.840 (CH_2), 0.681 (CH_3).[S19] [S19b] The ATR-IR spectra depict clear peaks at 2927, 1716, and 1132, confirming the CH_3, C=O, and C-O-C bonds in the PBzMA.

A.3.4 Procedure of Complex **1-mediated** OMRP of BzMA. Complex **1**-mediated RDRP of BzMA (P2, Table S8), a glass vial (20 mL) charged with Complex **1** (7 mg, 0.123 mmol), EBiB (18 µL, 0.123 mmol), Me$_6$TREN (33 µL, 0.123 mmol), Anhydrous THF(875 mg, 3.99 mmol), and degassed BzMA (2 mL, 12.31 mmol) is sealed with a silicone rubber septum inside a glove box. The reaction mixture was then stirred for 24 hrs. At the end of the reaction, the crude product was dissolved in THF, the excess solvent was evaporated by rotary evaporator, and the polymer was isolated by precipitation into prechilled methanol (×3 S4 times). Finally, the product was dried under vacuum (10^{-3} bar, 60 °C) for 12 h and characterized by NMR spectroscopy and SEC. ^{1}H NMR spectra of PBzMA$_{50}$-Br exhibited the typical signals of PBzMA at 7.336-7.246 (H–Ar), 5.090-4.971 (H_2C–Ar), 1.927 (CH_3); 0.840 (CH_2), 0.681 (CH_3). A similar procedure was carried out with complex **2.**

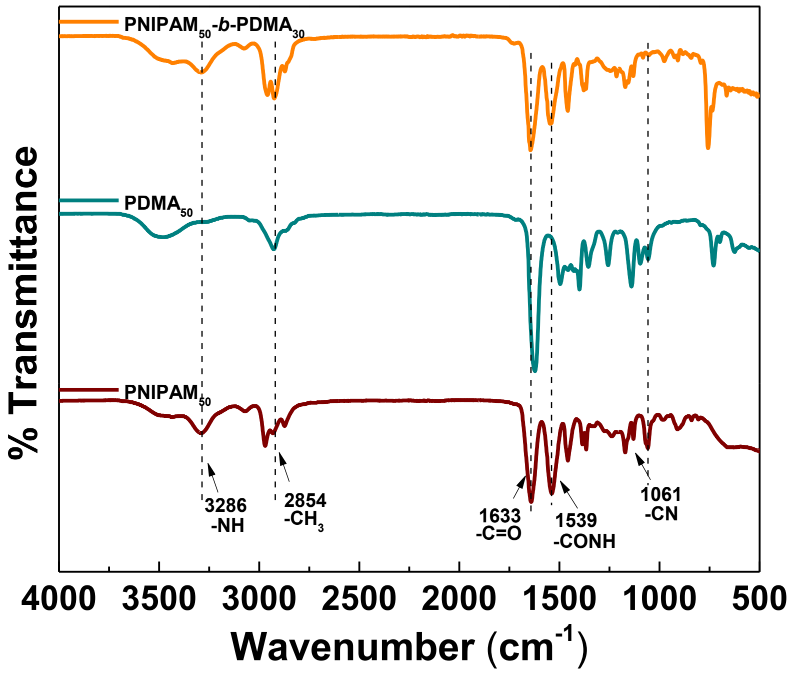

Figure S32. ATR-IR spectra of the block copolymer of PNIPAM$_{50}$-*b*-PDMA$_{30}$-Br.

The ATR-IR spectrum of PNIPAM$_{50}$-*b*-PDMA$_{30}$ exhibited the main characteristic peaks of the PNIPAM and PDMA 1061, 1539, 1633, 2854, and 3286 cm^{-1}, attributed to -CN, -CONH, -CO, -CH$_3$, and -NH, respectively.

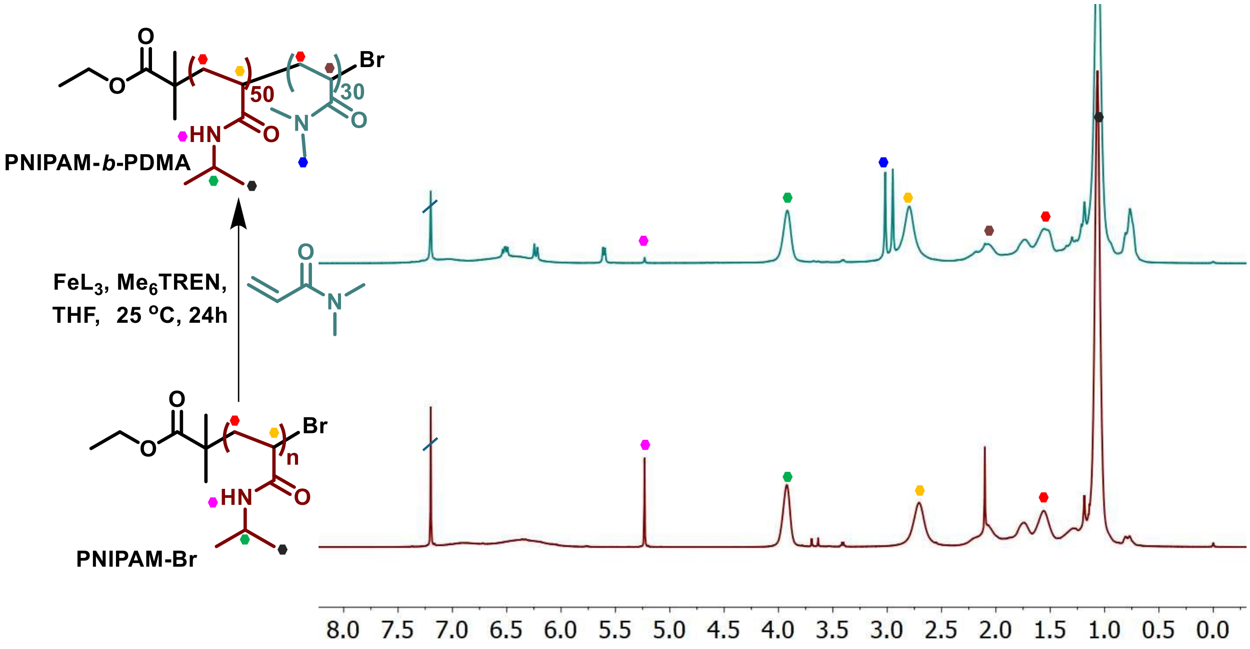

Figure S33. ^{1}H NMR spectra of PNIPAM$_{50}$-*b*-PDMA$_{30}$-Br.

^{1}H NMR spectra of the PNIPAM$_{50}$-*b*-PDMA$_{30}$ diblock copolymer (**P2, Table S13**) shows the distinguishing signals of the PNIPAM, first block and PDMA second block at 1.0-1.2 ppm (–CH(C$\underline{H}_3$) of NIPAM repeat, 1.4-2.2 ppm (–CH-C$\underline{H}_2$ of the NIPAM repeat unit and (–CH$_2$C(C$\underline{H}_3$) of DMA), 1.5–2.1 ppm (-CCH$_2$ connecting DMA chains), 2.1–2.5 ppm (-CH of DMA chains), 2.6 ppm (–N(C$\underline{H}_3$)$_2$ of DMA), 2.7-3.4 ppm (–CH-CH$_2$ of the NIPAM repeat unit), 3.6-4.0 ppm (–CH(CH$_3$) of NIPAM repeat unit adjacent to -CONH), 5.3-5.7 ppm (–CONH of NIPAM)[S17b]

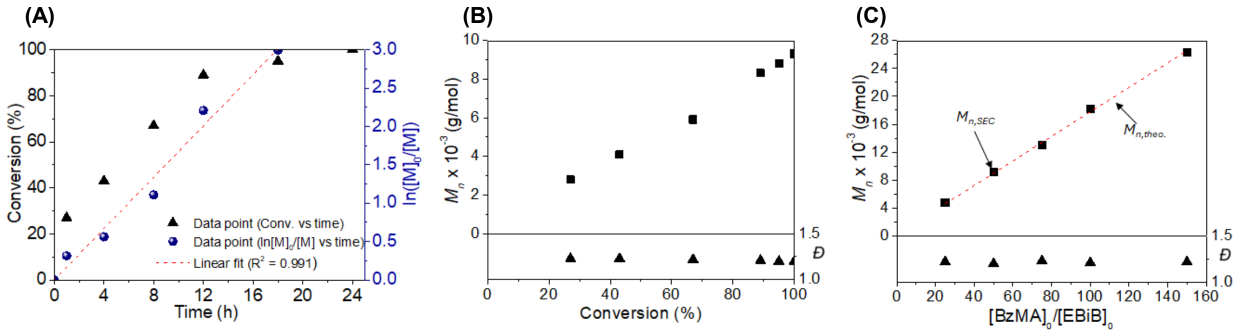

Figure S34. (A) kinetic plot ln([M]$_0$/[M]) vs time for the PBzMA$_{50}$. (B)Plots of M_n and $Đ_s$ vs conversion for the OMRP of BzMA mediated by complex **1** at 25 °C in THF. (C) plot for M_n and $Đ_s$ vs monomer conversion.

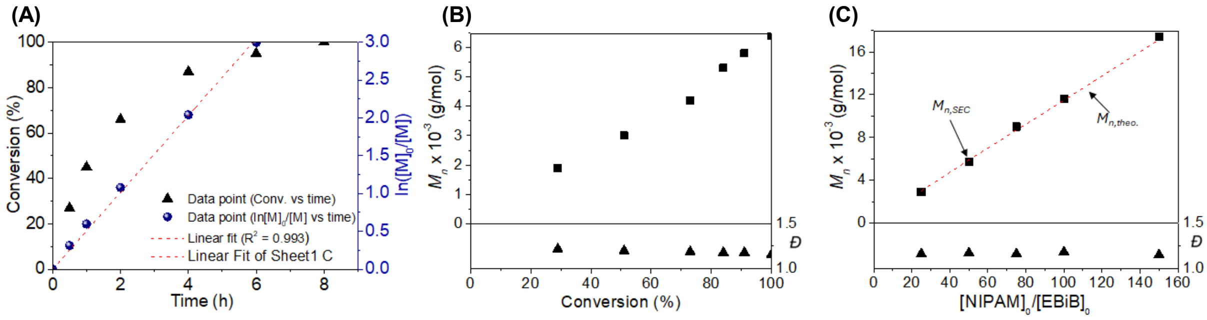

Figure S35. (A) kinetic plot ln([M]$_0$/[M]) vs time for the PNIPAM$_{50}$. (B)Plots of M_n and $Đ_s$ vs conversion for the OMRP of NIPAM mediated by complex **1** at 25 °C in THF. (C) plot for M_n and $Đ_s$ vs monomer conversion.

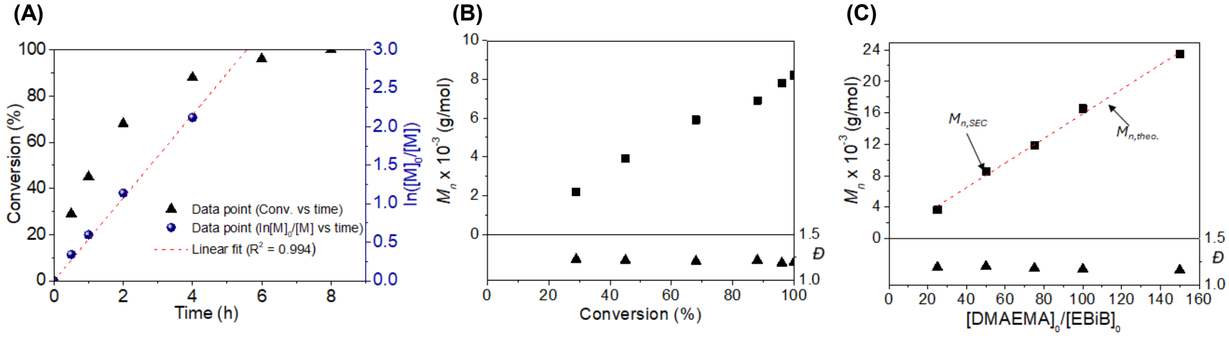

Figure S36. (A) kinetic plot ln([M]$_0$/[M]) vs time for the PDMAEMA$_{50}$. (B)Plots of M_n and $Đ_s$ vs conversion for the OMRP of DMAEMA mediated by complex **1** at 25 °C in THF. (C) plot for M_n and $Đ_s$ vs monomer conversion.

12. Mössbauer Measurements

Mössbauer spectrum was recorded with a ^{57}Co source in a Rh matrix using an alternating constant acceleration *Wissel* Mössbauer spectrometer operated in the transmission mode and equipped with a *Janis* closed-cycle helium cryostat. Isomer shift is given relative to iron metal at ambient temperature.

Simulation of the experimental data was performed with the *mf2.SL* program using Lorentzian line doublets: E. Bill, Max-Planck Institute for Chemical Energy Conversion, Mülheim/Ruhr, Germany.

13. EPR and DFT studies of complex 2

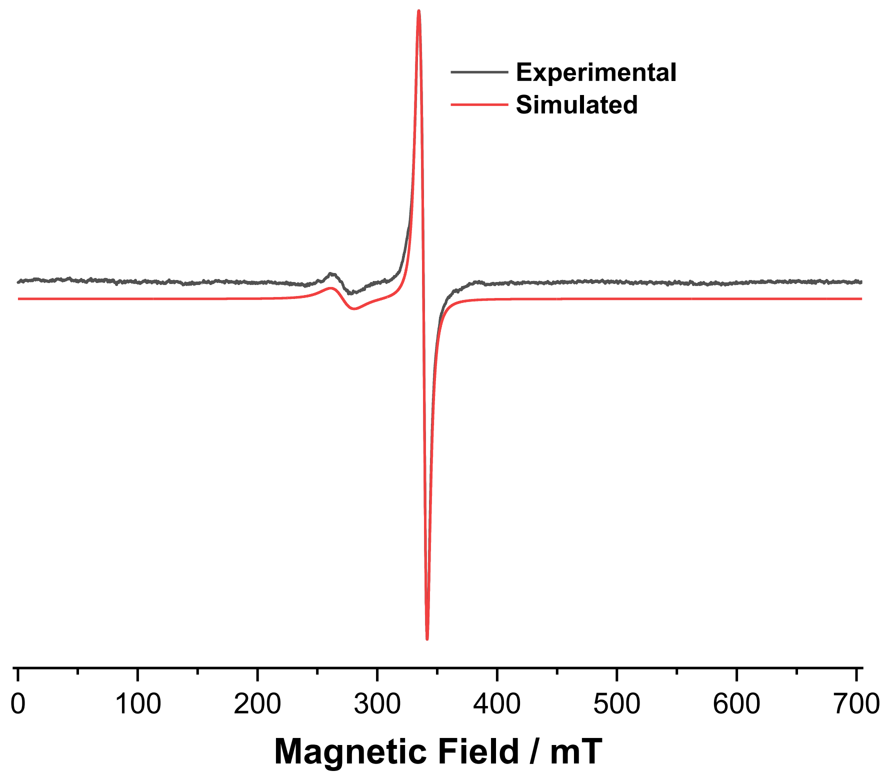

Figure S37. X-band EPR spectrum (black) of the complex [Fe(III)(SS-NHC=Se)$_3$] (**2**) at room temperature in solid state. Red and black lines represent the simulated and the experimental spectra of the **2** using the EasySpin program. [$\mathbf{g_{\perp}}$ **(Radical unpaired e$^-$)** = 1.98405, $\mathbf{g_{\parallel}}$ **(Radical unpaired e$^-$)** = 2.01898, LWPP (Gaussian broadening – Radical e$^-$ signal) = 0.0298646 mT, LWPP (Lorentzian broadening – Radical e$^-$ signal) = 4.75362 mT, $\mathbf{g_{iso}}$ **(Metal unpaired e$^-$)** = 2.49105, LWPP (Gaussian broadening – Metal e$^-$ signal) = 9.61341 mT, LWPP (Lorentzian broadening – Metal e$^-$ signal) = 14.9917 mT, X-band experimental frequency = 9.446327 GHz].

$$g_{iso(radical\ unpaired\ e^-)} = \sqrt{\frac{2g_{\perp}{}^2 + g_{\parallel}{}^2}{3}} = \sqrt{\frac{2 \times 1.98405^2 + 2.01898^2}{3}} = 1.995761262$$

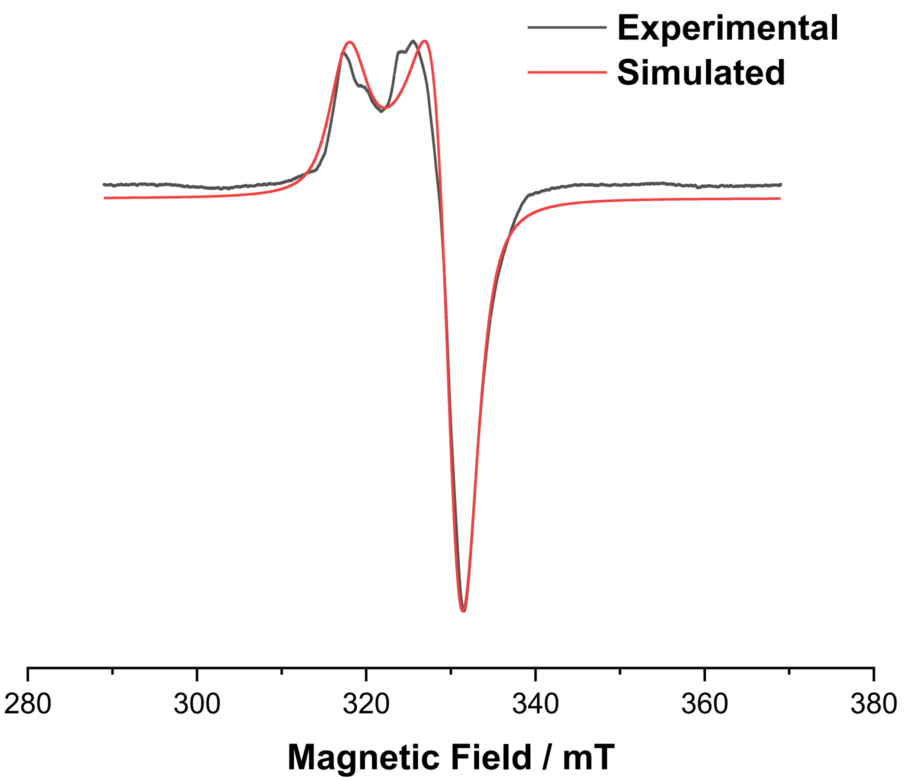

Figure S38. X-band EPR spectrum (black) of the [Fe(III)(SS-NHC=Se)$_3$] (**2**) at liquid nitrogen temperature in solid state. Red and black lines represent the simulated and the experimental spectra of the **2** using the EasySpin program. [**g**$_\perp$ **(Radical unpaired e$^-$)** = 1.98514, **g**$_\parallel$ **(Radical unpaired e$^-$)** = 2.0638, LWPP (Gaussian broadening – Radical e$^-$ signal) = 2.49987 mT, LWPP (Lorentzian broadening – Radical e$^-$ signal) = 1.87853 mT, X-band experimental frequency = 9.179467 GHz].

$$g_{iso(radical\ unpaired\ e^-)} = \sqrt{\frac{2{g_\perp}^2 + {g_\parallel}^2}{3}} = \sqrt{\frac{2 \times 1.98514^2 + 2.0638^2}{3}} = 2.011701774$$

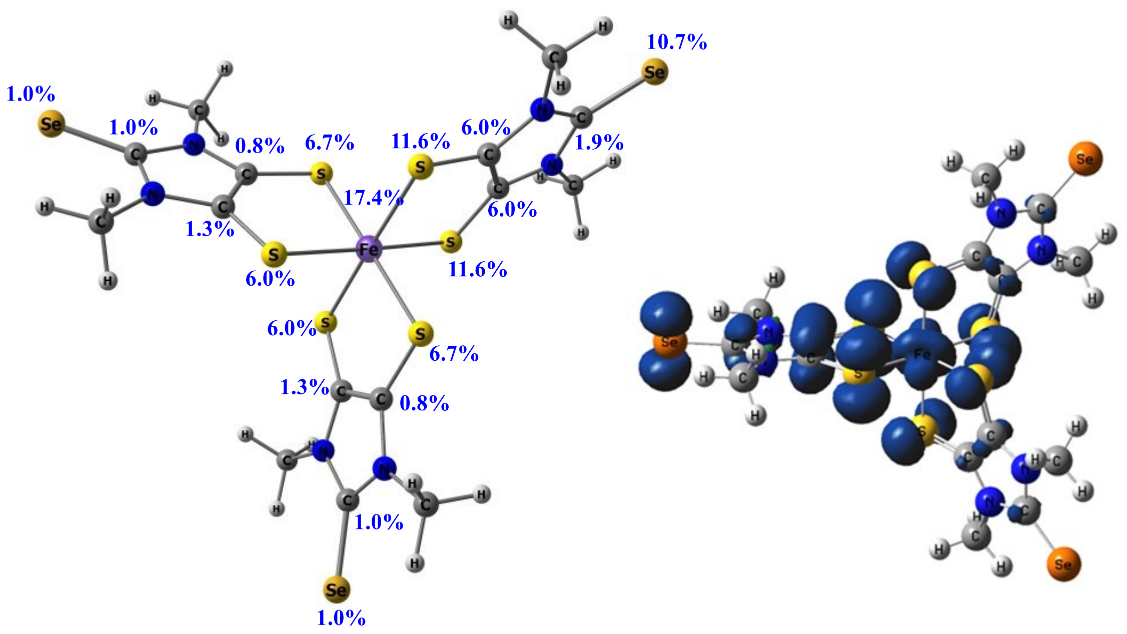

Figure S39. The Spin density distribution (in %, left) and Spin density plot (right) for complex **2′** in triplet electronic state as calculated at B3LYP-D3(BJ)/def2TZVP level of theory. The alpha spin density is shown in blue colour. The percentage is calculated with respect to the total alpha spin density value. Dip is replaced in complex **2** by methyl in **2′**.

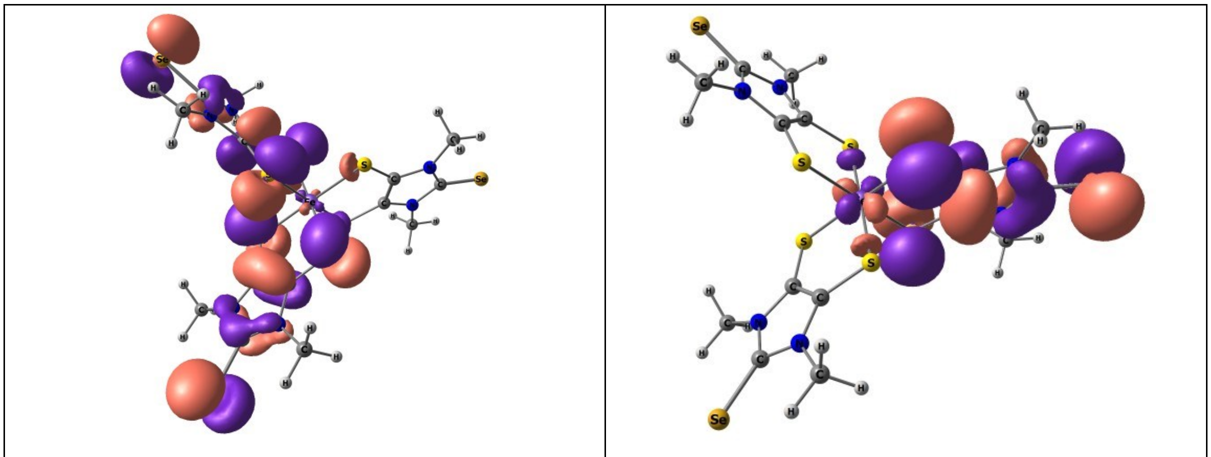

SOMO-1 (ε = -5.12 eV)	SOMO (ε = -4.74 eV)

Figure S40. The molecular orbitals for complex **2′** in triplet electronic state are calculated at B3LYP-D3(BJ)/def2TZVP level of theory. The SOMO-1 and SOMO represent the two half-filled orbitals. Dip is replaced in complex **2** by methyl in **2′.**

Complex **2′** (Triplet)

E = -11769.4467189 hf

26	0.218957000	0.000005000	0.000001000
16	1.864527000	-0.716013000	-1.519408000
16	-1.404174000	0.767635000	1.470189000
16	1.864524000	0.716029000	1.519407000
16	0.204299000	-1.997822000	1.191027000
16	0.204281000	1.997827000	-1.191026000
16	-1.404170000	-0.767640000	-1.470185000
7	4.559991000	0.470766000	0.989680000
7	4.559993000	-0.470731000	-0.989685000
7	-2.838754000	3.064630000	0.942077000
7	-1.785415000	3.870573000	-0.805281000
7	-1.785382000	-3.870585000	0.805281000
7	-2.838729000	-3.064648000	-0.942075000
6	5.395418000	0.000018000	-0.000002000
6	3.244743000	-0.301059000	-0.633962000
6	3.244742000	0.301086000	0.633959000
6	-1.826299000	2.185211000	0.653142000
6	-2.833048000	4.119992000	0.055021000
6	-1.149714000	2.702096000	-0.467595000
6	-1.149691000	-2.702102000	0.467597000
6	-2.833013000	-4.120013000	-0.055021000
6	-1.826282000	-2.185221000	-0.653139000
6	-1.382935000	4.717751000	-1.912197000
1	-1.331227000	4.119759000	-2.821251000
1	-2.119670000	5.508340000	-2.017797000
1	-0.401202000	5.147031000	-1.711893000

6	-3.768435000	2.892954000	2.042546000
1	-4.513215000	3.680701000	1.981182000
1	-4.241330000	1.914564000	1.965130000
1	-3.234445000	2.956350000	2.990642000
6	-3.768413000	-2.892979000	-2.042544000
1	-4.513182000	-3.680736000	-1.981184000
1	-4.241320000	-1.914596000	-1.965123000
1	-3.234421000	-2.956364000	-2.990640000
6	-1.382893000	-4.717761000	1.912195000
1	-1.331181000	-4.119768000	2.821248000
1	-2.119625000	-5.508352000	2.017800000
1	-0.401160000	-5.147040000	1.711886000
6	4.987187000	-1.065789000	-2.241709000
1	4.595738000	-2.080059000	-2.316092000
1	4.607003000	-0.475711000	-3.075299000
1	6.072914000	-1.079451000	-2.254723000
6	4.987183000	1.065821000	2.241707000
1	4.595650000	2.080055000	2.316133000
1	4.607081000	0.475687000	3.075296000
1	6.072909000	1.079571000	2.254682000
34	-3.967046000	-5.536404000	-0.025199000
34	7.211669000	0.000019000	0.000000000
34	-3.967095000	5.536372000	0.025197000

14. Raman Spectrum:

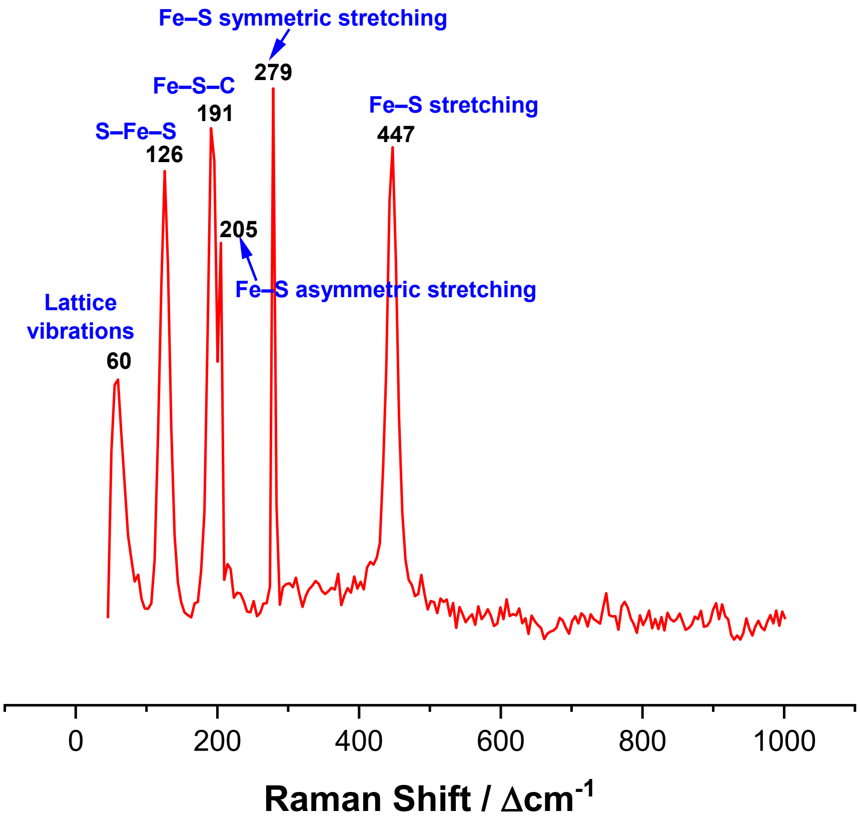

Figure S41. Solid state Raman spectrum of [Fe(III)(SS-NHC=S)$_3$] **(1)** measured in alpha300 R Raman Microscope using 532 nm laser.

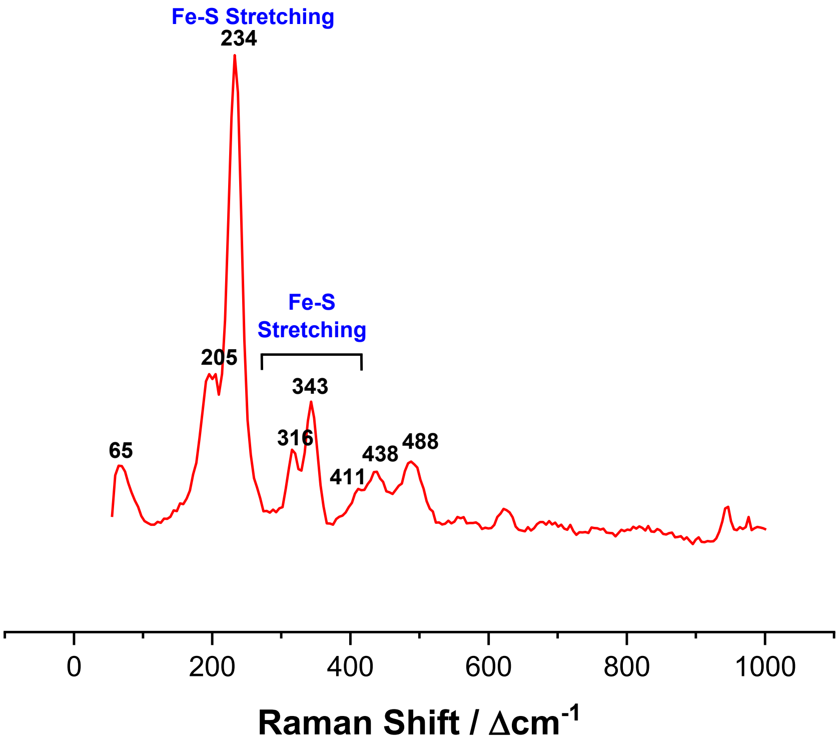

Figure S42. Solid state Raman spectrum of [Fe(III)(SS-NHC=Se)$_3$] **(2)** measured in alpha300 R Raman Microscope using 532 nm laser.

15. References:

S1. A. D. Becke, *J. Chem. Phys.* **1993**, *98*, 1372-1377.

S2. P. J. Stephens, F. J. Devlin, C. F. Chabalowski, M. J. Frisch, *J. Phys. Chem.* **1994**, *98*, 11623-11627.

S3. C. Lee, W. Yang, R. G. Parr, *Phys. Rev. B.* **1988**, *37*, 785-789.

S4. S. Grimme, J. Antony, S. Ehrlich, H. Krieg, *J. Chem. Phys*. **2010**, *132*, 154104-154124.

S5. S. Grimme, S. Ehrlich, L. Goerigk, *J. Comput. Chem.* **2011**, *32*, 1456-1465.

S6. F. Weigend, R. Ahlrichs, *Phys. Chem. Chem. Phys.* **2005**, *7*, 3297-3305.

S7. Gaussian 16, Revision A.03, M. J. Frisch, G. W. Trucks, H. B. Schlegel, G. E. Scuseria, M. A. Robb, J. R. Cheeseman, G. Scalmani, V. Barone, G. A. Petersson, H. Nakatsuji, X. Li, M. Caricato, A. V. Marenich, J. Bloino, B. G. Janesko, R. Gomperts, B. Mennucci, H. P. Hratchian, J. V. Ortiz, A. F. Izmaylov, J. L. Sonnenberg, D. Williams-Young, F. Ding, F. Lipparini, F. Egidi, J. Goings, B. Peng, A. Petrone, T. Henderson, D. Ranasinghe, V. G. Zakrzewski, J. Gao, N. Rega, G. Zheng, W. Liang, M. Hada, M. Ehara, K. Toyota, R. Fukuda, J. Hasegawa, M. Ishida, T. Nakajima, Y. Honda, O. Kitao, H. Nakai, T. Vreven, K. Throssell, J. A. Montgomery, Jr., J. E. Peralta, F. Ogliaro, M. J. Bearpark, J. J. Heyd, E. N. Brothers, K. N. Kudin, V. N. Staroverov, T. A. Keith, R. Kobayashi, J. Normand, K. Raghavachari, A. P. Rendell, J. C. Burant, S. S. Iyengar, J. Tomasi, M. Cossi, J. M. Millam, M. Klene, C. Adamo, R. Cammi, J. W. Ochterski, R. L. Martin, K. Morokuma, O. Farkas, J. B. Foresman, and D. J. Fox, Gaussian, Inc., Wallingford CT, **2016**.

S8. E. D. Glendening, C. R. Landis, F. Weinhold, *J. Comput. Chem.* **2013**, *34***,** 1429-1437.

S9. A. E. Reed, L. A. Curtiss, F. Weinhold, *Chem. Rev.* **1988**, *88*, 899-926.

S10. T. Ziegler, A. Rauk, *Theor. Chim. Acta* **1977**, *46*, 1–10.

S11. M. Mitoraj, A. Michalak, *Organometallics* **2007**, *26*, 6576 – 6580.

S12. M. Mitoraj, A. Michalak, *J. Mol. Model.* **2008**, *14*, 681-687.

S13. *ADF2020, SCM, Theoretical Chemistry*; Vrije Universiteit: 782 Amsterdam, The Netherlands http://www.scm.com

S14. A. Michalak, M. Mitoraj, T. Ziegler, *J. Phys. Chem. A.* **2008**, *112*, 1933-1939.

S15. A. Michalak, M. Mitoraj, T. Ziegler, *J. Chem. Theory Comput*. **2009**, *5*, 962-975.

S16. R. Tamaki, K. Naka, Y. Chujo, *J. Polym.* **1998**, *30*, 60-65.

S17. (a) X. Gao, Y. Cao, X. Song, Z. Zhang, C. Xiao, C. He, X. Chen, *J. Mater. Chem. B* **2013**, *1*, 5578-5587. (b) S. A. Mohammad, S. Dolui, D. Kumar, M. M. Alam, S. Banerjee, *Macromol. Rapid Commun.* **2021**, *42*, 2100096.

S18. D. Kumar, B. Sahu, S. Dolui, S. S. Rajput, M. M. Alam, S. Banerjee, *Eur. Polym. J.* **2023**, *199*, 112443.

S19. (a) T. G. Oberti, M. M. Schiavoni, M. S. Cortizo, *Radiat. Phys. Chem.* **2008**, *77*, 597-604. (b) Y. T. Cheng, Q. Xia, H. Liu, M. B. Solomon, E. R. Brisson, L. D. Blackman, C. D. Ling, M. Müllner, *ACS Appl. Mater. Interfaces.* **2023**, *15*, 12261-12272.
